# Accelerating the Hit-To-Lead Optimization of a SARS-CoV-2 Mpro Inhibitor Series by Combining High-Throughput Medicinal Chemistry and Computational Simulations

**DOI:** 10.1021/acs.jmedchem.4c02941

**Published:** 2025-04-05

**Authors:** Julien Hazemann, Thierry Kimmerlin, Aengus Mac Sweeney, Geoffroy Bourquin, Roland Lange, Daniel Ritz, Sylvia Richard-Bildstein, Sylvain Regeon, Paul Czodrowski

**Affiliations:** † Drug Discovery Chemistry, Idorsia Pharmaceuticals Limited, Hegenheimermattweg 91, 4123 Allschwil, Switzerland; ‡ Drug Discovery Biology, Idorsia Pharmaceuticals Limited, Hegenheimermattweg 91, 4123 Allschwil, Switzerland; § Chemistry Department, Johannes Gutenberg University, Duesbergweg 10-14, 55128 Mainz, Germany

## Abstract

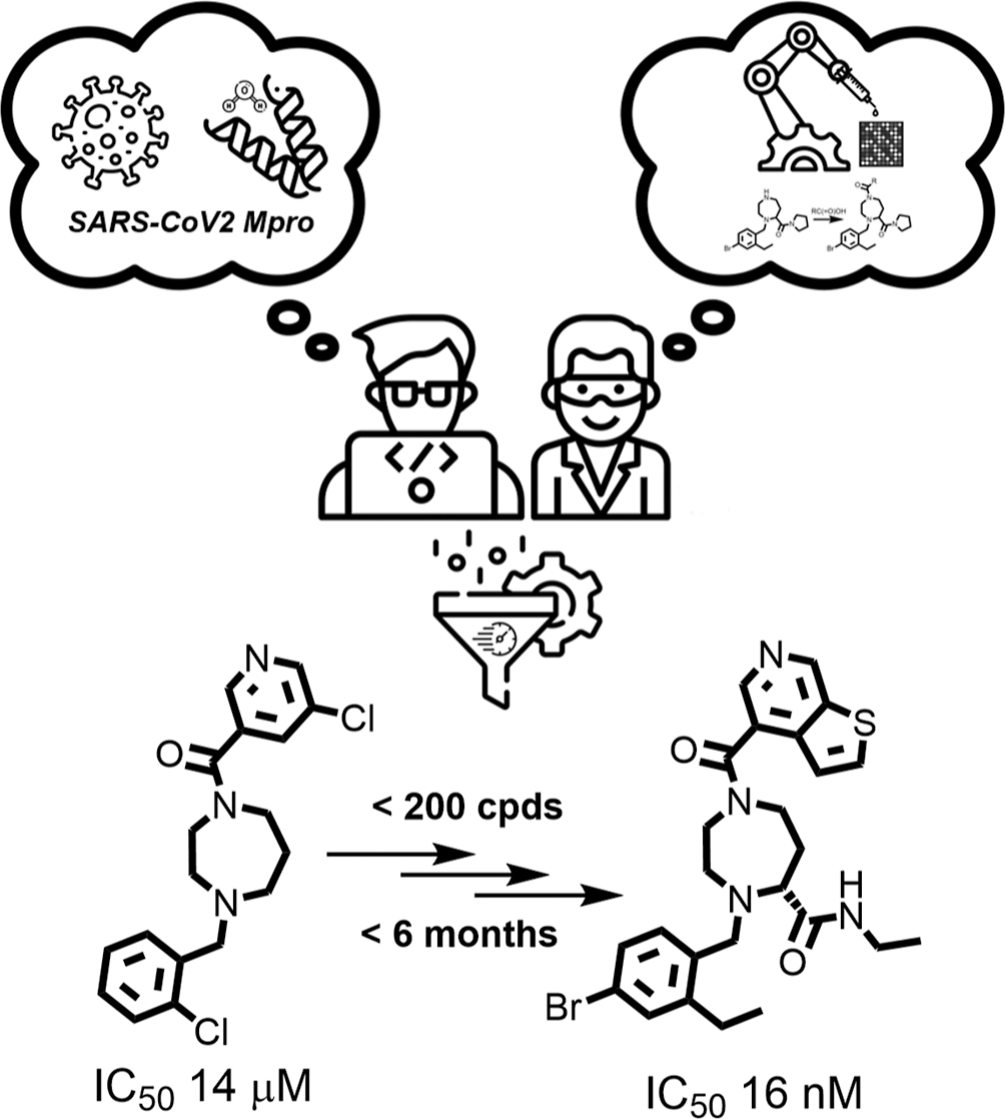

In this study, we performed the hit-to-lead optimization of a SARS-CoV-2 Mpro diazepane hit (identified by computational methods in a previous work) by combining computational simulations with high-throughput medicinal chemistry (HTMC). Leveraging the 3D structural information of Mpro, we refined the original hit by targeting the S1 and S2 binding pockets of the protein. Additionally, we identified a novel exit vector pointing toward the S1′ pocket, which significantly enhanced the binding affinity. This strategy enabled us to transform, rapidly with a limited number of compounds synthesized, a 14 μM hit into a potent 16 nM lead compound, for which key pharmacological properties were subsequently evaluated. This result demonstrated that combining computational technologies such as machine learning, molecular docking, and molecular dynamics simulation with HTMC can efficiently accelerate hit identification and subsequent lead generation.

## Introduction

The outbreak of the coronavirus disease in 2019 has increased the pressure on the pharma industry to shorten the identification and the development time of new drugs to cure life-threatening diseases.

A possible approach is drug repurposing,^2^ i.e., using a previously approved drug on a new target and in a new indication. Molnupiravir^3^ (Lagevrio, MSD) and Paxlovid^4^(Pfizer) are two examples of repurposed antiviral drugs approved by the FDA. Molnupiravir, originally developed as treatment of influenza viruses and encephalitic alphaviruses, is a prodrug of *N*^4^-hydroxycytidine that targets the RNA-dependent RNA polymerase enzyme essential for the genome replication of RNA viruses. Paxlovid is a combination of nirmatrelvir, a covalent inhibitor of Mpro, the 3CL main protease of SARS-CoV-2, and ritonavir, an inhibitor of CYP450-3A4 leading to the enhanced efficacy of nirmatrelvir by reducing drug metabolism.

Traditionally in the pharmaceutical industry high-throughput screening is the technology of choice for the identification of a new chemical starting point. Over the years, this technology has proven to be successful^5^ but only a small part of the available chemical space—a few million compounds from the 10^60^ molecules of the estimated drug-like chemical space^6^—can be explored physically and the process remains costly and time consuming. DNA-encoded libraries (DEL) have recently emerged as a powerful complementary screening platform.^5,7^ In a DEL, each compound in the library is linked to a unique DNA tag allowing hit identification via next-generation sequencing. The DEL technology enables the simultaneous synthesis, processing, and selection of millions to billions of compounds in just one vial. Over the past decade, DELs have gained widespread use in drug discovery and demonstrated potential in academic research.^8^ However, challenges and limitations exist in DEL. Some of these limitations include the incompatibility of some building blocks or reactions and the potential effect of the oligonucleotide on binding affinity.^9^ More recently, computational methods such as artificial intelligence (AI) and machine learning (ML) have gained a lot of interest also in drug discovery.^10^ The applicability of the ML technology for hit finding has been illustrated by the identification of new inhibitors of the human arylamine *N*-acetyltransferase (ANAT) enzyme, a target previously considered as “undruggable”.^11^ Also, ML has been used to virtually screen large DEL libraries at a fraction of the cost of a traditional DEL screen.^12^

The technologies mentioned above have the power to accelerate the identification of new bioactive starting points, but it remains essential to optimize and streamline all the processes involved in the transformation of an initial hit structure into a drug candidate.

In this regard high-throughput medicinal chemistry (HTMC) is a very important tool for medicinal chemist.^13^ HTMC consists of performing multiple reactions in parallel to generate small to medium size compound libraries with high speed and high efficiency. HTMC can cover a wide scope of reaction types including standard transformations but also new protocols such as photo-redox chemistry giving access to a large diversity of structures. This is a fully automated process including synthesis, purification QC, and reformatting. The main purpose is to shorten turnaround time of the design, make, test, and analysis cycle.

**Figure 1:**
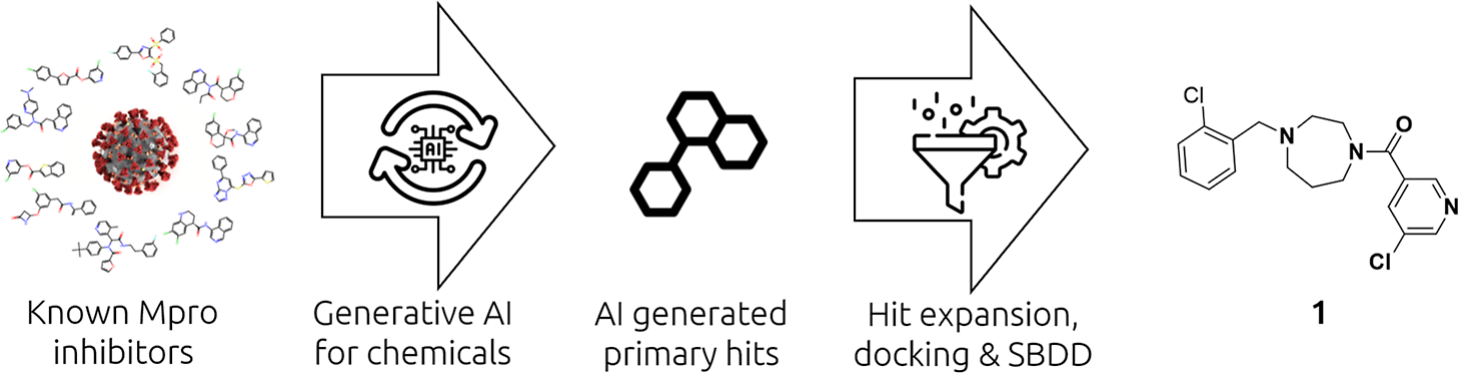
Previously published workflow^1^ used for the identification of a new class of Mpro inhibitor using generative AI for molecules, hit expansion, docking and SBDD. Compound **1** was selected for the hit-to-lead campaign.

The starting point of our work was discovered from a previous study through the identification of new inhibitors for the Mpro enzyme by combining deep reinforcement learning for de novo drug design with 3D pharmacophore/shape-based alignment and matching privileged fragments. A deep generative model (DGM) was retrained using Mpro inhibitors from the Covid Moonshot project^14^ and the ChEMBL database.^15^ The DGM was optimized via reinforcement learning to prioritize privileged fragment identification and 3D alignment. AI-generated molecules led to primary hits, which were expanded through similarity searches in commercial and corporate collections to find new analogs. A novel hit was identified and validated through structure-based drug design (SBDD), leading to the synthesis of a promising compound (compound **1**; please note that in our previous publication, compound **1** was referred to as compound **11**) from which the X-ray crystal structure was obtained.^1^ The workflow leading to the identification of compound **1** is represented in Figure 1.

In this publication, we report the hit-to-lead campaign performed around compound **1**, using an integrated workflow that combines SBDD and HTMC, resulting in the identification of a new Mpro inhibitor lead series. The chemical structure of this newly identified diazepane series is very similar to the recently published piperazine series.^16,17^ However, a comparison of inhibitory activity, binding mode, and druglike properties provides insights into both similarities and, moreover, differences that help assess the potential for advancing the diazepane series into lead optimization.^16,17^

## Results

### Crystal Structure of Compound **1**

**Figure 2:**
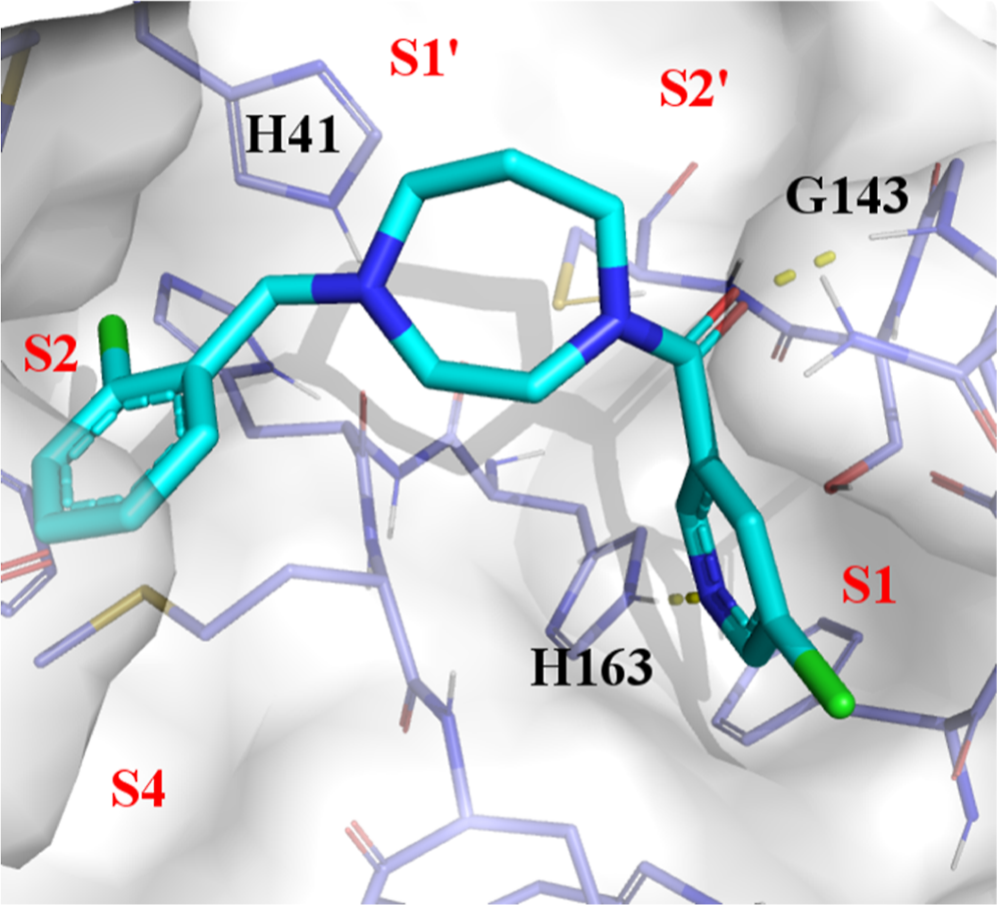
Structure of compound **1** (PDB code: 8r14) in complex with Mpro. The *m*-chloropyridine moiety sits in the S1 pocket making a direct H-bond interaction between the N of the pyridine ring and H163. The *m*-chlorophenyl ring is filling the hydrophobic S2 pocket.

The X-ray structure of Mpro in complex with compound **1** (Figure 2) has been obtained from our previous work. The binding of compound **1** within the active site of Mpro is characterized by the following features: the *m*-chloropyridine sits in the S1 pocket and makes a key H-bond interaction with H163. In addition, the amide carbonyl interacts with G143. In the S2 pocket, the *m*-chlorophenyl sits in a hydrophobic environment making a face-to-edge aryl interaction with H41.

### WaterMap Analysis of Compound **1**

In standard docking applications, a critical variable that often remains inadequately accounted for, yet can significantly influence structure–activity relationship (SAR) trends and the design of new compounds, is the energy and spatial distribution of water molecules within the binding site.^18^ To address this, we performed a WaterMap (Schrödinger) analysis to evaluate the water network and identify high-energy water molecules that may be suitable for displacement. WaterMap is a molecular dynamics (MD)-based computational approach that leverages statistical mechanics to characterize the thermodynamic properties (entropy, enthalpy, and free energy) of water molecules residing within the binding site.^19^ This method facilitates the assessment of solvent contributions to ligand binding affinity and offers valuable insights for lead optimization.

**Figure 3:**
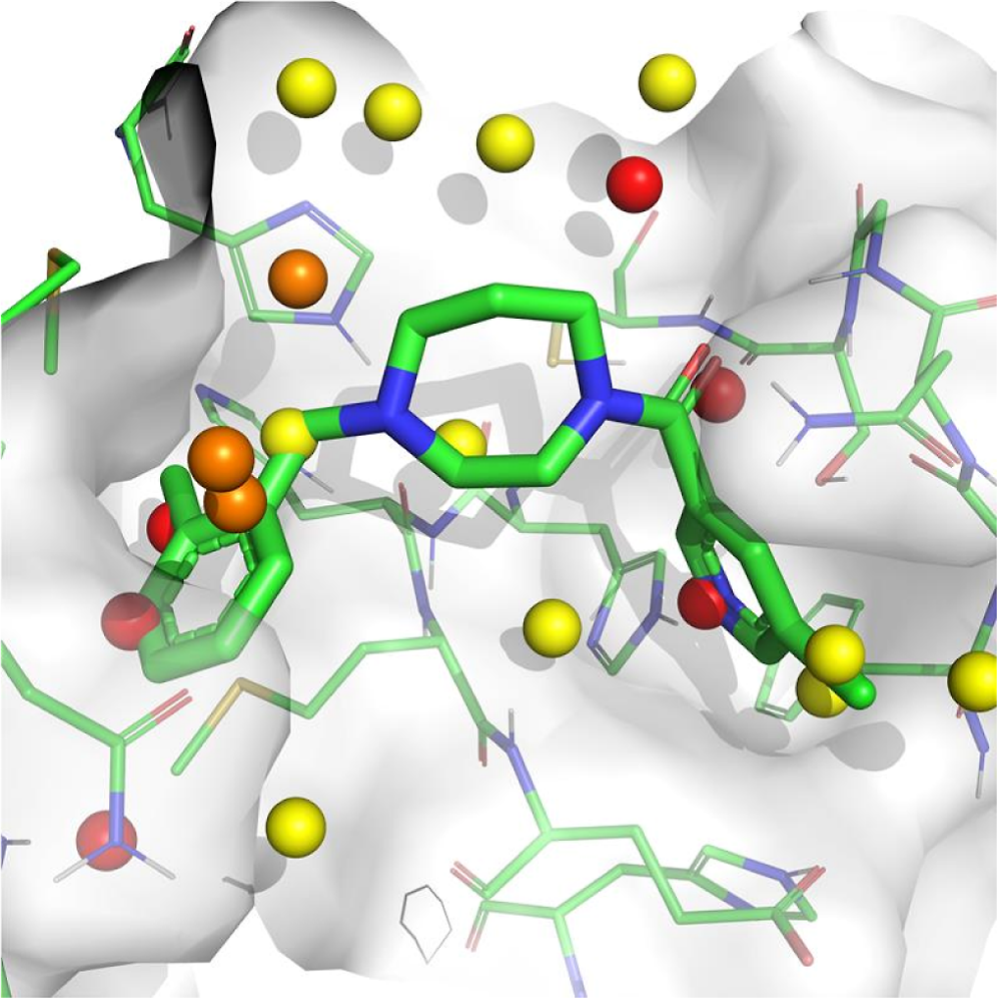
WaterMap analysis of Mpro in complex with compound **1** with color-coded unstable water molecules represented as spheres. Yellow spheres: 1 kcal/mol < Δ*G* > 2 kcal/mol, orange spheres: 2 kcal/mol < Δ*G* > 3 kcal/mol and red spheres: Δ*G* > 3 kcal/mol.

The WaterMap analysis identified unstable water molecules in the S1, S2, S4, and S2′ pockets, as depicted in Figure 3. We therefore hypothesized that combining docking together with WM/MM, in which the WaterMap displacement energy is combined with energy components from MM-GBSA, would lead to better outcomes.

### Optimization of the Binding of Compound **1** into the S2 Pocket

**Figure 4:**
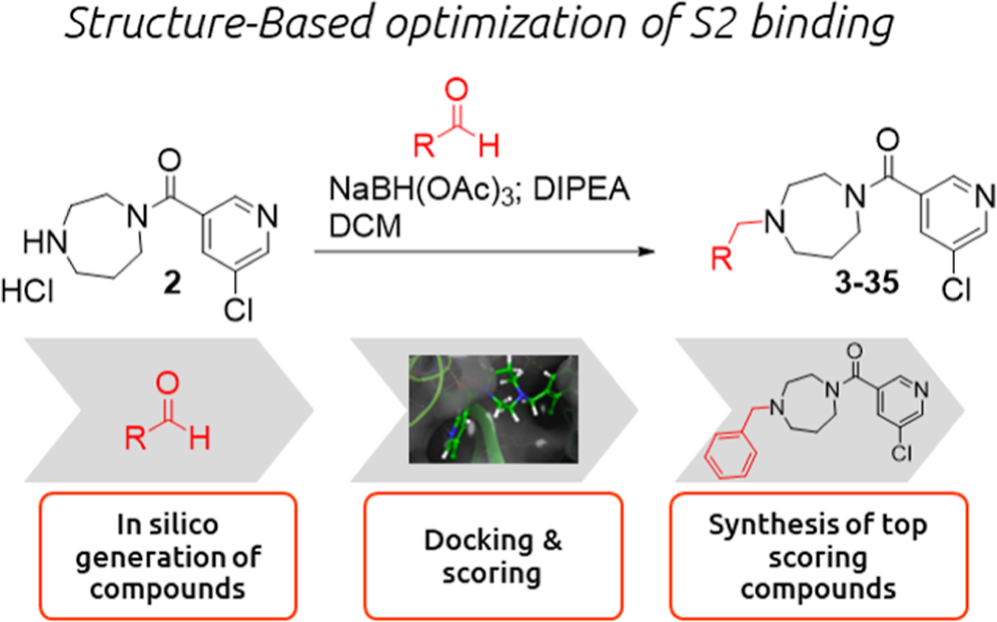
Optimization of the binding into the S2 pocket was achieved by using the following workflow: in silico generation of a virtual library based on a reductive amination reaction using in-house building blocks.; docking of the virtual compounds into the active site of the enzyme; synthesis of the best scoring compounds and assessment of the inhibitory activity in a enzymatic FRET assay.

With the goal of rapidly optimizing the potency of the initial hit structure **1** and establishing a SAR for the binding into the S2 pocket, we combined structure-based design with parallel synthesis. To do so, we enumerated a virtual library of 1600 molecules based on a reductive amination reaction using a set of our in-house stock of aldehydes (Figure 4). The enumerated molecules were docked into the previously obtained X-ray structure using the following constraints: template docking (compound **1** MCS) and H-bond H163 and G143. After visual inspection (human experience rating), 330 molecules were pre-selected. A consensus score (visual inspection score, docking score, and WM/MM) was calculated. Compounds with a score >0.5 were selected. In total, 100 molecules were prioritized for synthesis by HTMC and the inhibitory activities of the synthesized compounds on the Mpro enzyme were assessed in a fluorescence resonance energy transfer (FRET)-based enzymatic assay.

This initial set of compounds led to the identification of compound **19** and **28** displaying more than 10-fold increase in the inhibitory activity as compared to original compound **1** with an IC_50_ in the FRET assay of 770 and 900 nM, respectively (see compound **1**, **19**, and **28** in Figure 6). Also, a clear SAR could be established. A phenyl ring with an ortho substituent is preferred over an alkyl and cycloalkyl moiety. Regarding the ortho substituent, the following order of potency was observed: Cl > CF3 > CN > Me > OMe. The phenyl ring can be replaced by a pyridine ring, but the position of the N is key (compare compound **1**, **8**, and **9** in Figure 6). Interestingly, compound **14** containing a 2-CN, 4-Cl-disubstituted phenyl ring is much more potent than the corresponding 4-Cl derivative **13** and, more potent than the corresponding 2-CN derivative **12**. The same observation is made with the 2,4-dichloro derivative **18** which is much more potent than the corresponding 2-Cl derivative **1** or 4-Cl derivative **13**. It appears that at the para position of the phenyl ring different type of substituent such as halogen, *O*-cycloalkyl, or nitrile are tolerated. However, increasing size and/or polarity of the substituent decreases the potency (see compound **20**, **21**, **22**, and **24**). A similar effect is observed at the ortho position of the phenyl ring; ethyl (compound **28**) is slightly better than methyl (compound **27**) while isobutyl (compound **30**) is too big and the more polar *O*-isopropyl (compound **31**) is even worse. All these observations are in agreement with the hydrophobic nature of the S2 pocket. Adding a third substituent at position 2′ is unfavorable (compound **34**), and the same negative effect is observed when adding a Me at the benzylic position (compare compound **1** and **35**).

**Figure 5:**
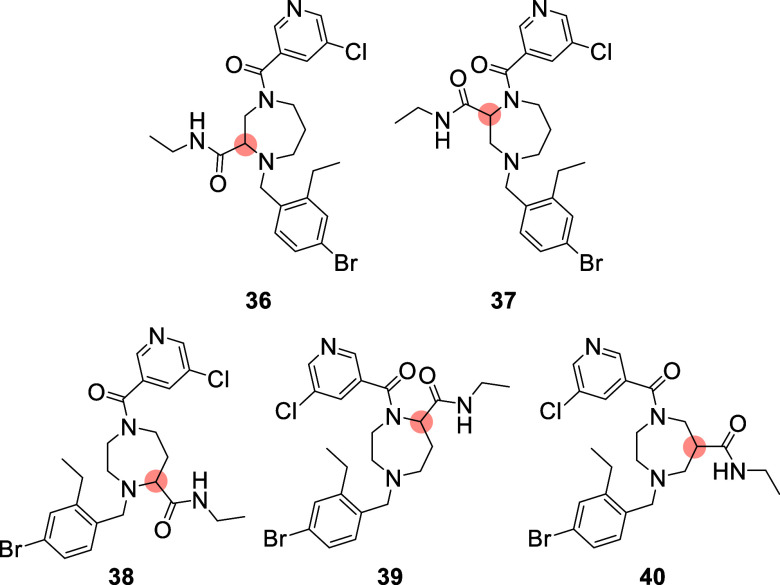
Structure of the five isomeric compounds bearing a new amide moiety as third exit vector on the diazepane ring.

**Figure 6:**
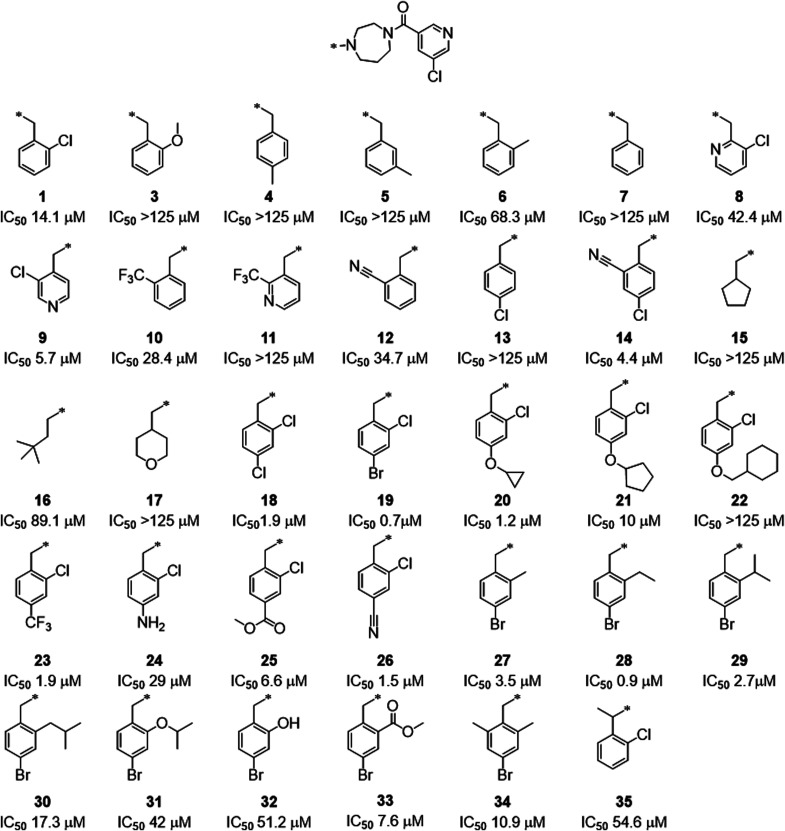
Chemical structures and inhibitory activities of compounds **1** to **35**.

### Introduction of a Third Exit Vector on the Diazepane Scaffold

Analysis of the X-ray structure of compound **1** bound into the active site of Mpro (Figures 2 and 3) indicates clearly that the S1′, S2′, and S4 pockets are not addressed by compound **1**. We hypothesized that adding an additional exit vector on the diazepane ring targeting one of these pockets would increase the inhibitory activity.

As shown in Figure 5, five positions are possible for the introduction of a third exit vector on the diazepane ring. For strategic reasons, we decided to include an amide functionality as this would allow later the rapid generation of analogs via amide coupling.

Prior to the synthesis, the ability of the five isomers (**36**; **37**; **38**; **39**; **40**) to bind into the active site of the enzyme was evaluated by computational methods. The molecules were docked into the binding pocket using the following procedure:

- Docking grid: hybrid complex made of Protein Data Bank (PDB) 7L13 for the protein and compound **1** for the ligand (the complex 7L13 was selected for the large 3D occupancy of its ligand to address the high plasticity of Mpro)
- Template docking (compound **1** MCS)
- H-bond constraints: H163 and G143
- Glide SP docking followed by XP docking.
- WM/MM Δ-*G* bind was calculated with the Schrödinger suite.

Compounds with a produced docking pose and for which the WM/MM Δ-*G* bind values were below −32 kcal/mol (compound **37**; **38**; **39**, and **40**) were considered further. Compound **36** was excluded as no docking pose was produced. To further assess an ideal exit vector, a 100 ns MD simulation was performed for compound **37**; **38**; **39**; and **40**.

**Figure 7:**
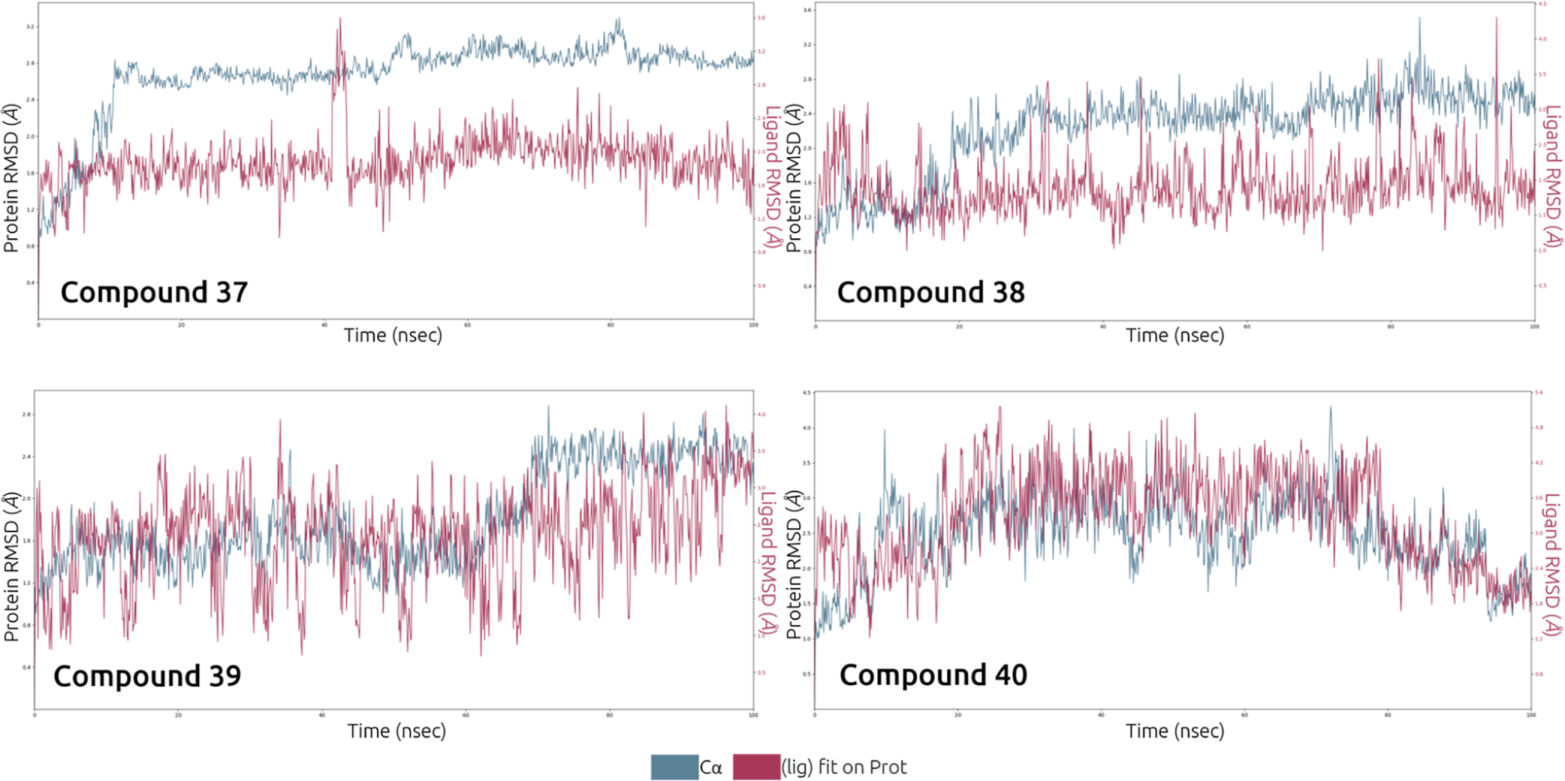
Protein–ligand RMSD throughout the MD simulation time. Protein RMSD in red and ligand RMSD in blue.

The root-mean-square deviation (RMSD) was used to quantify the average displacement of the protein or ligand over time in MD simulations, relative to the first frame. The RMSD values for the protein across different exit-vector positions were compared. As shown in Figure 7, compounds **37** and **38** prompted a rapid conformational change in the protein, after which the RMSD stabilized for the remainder of the simulation. In contrast, compounds **39** and **40** exhibited less stable RMSD behavior over the course of the 100 ns MD simulation. Based on these observations, we hypothesized that the most favorable exit vectors are associated with compounds **37** and **38**.

**Scheme 1:**
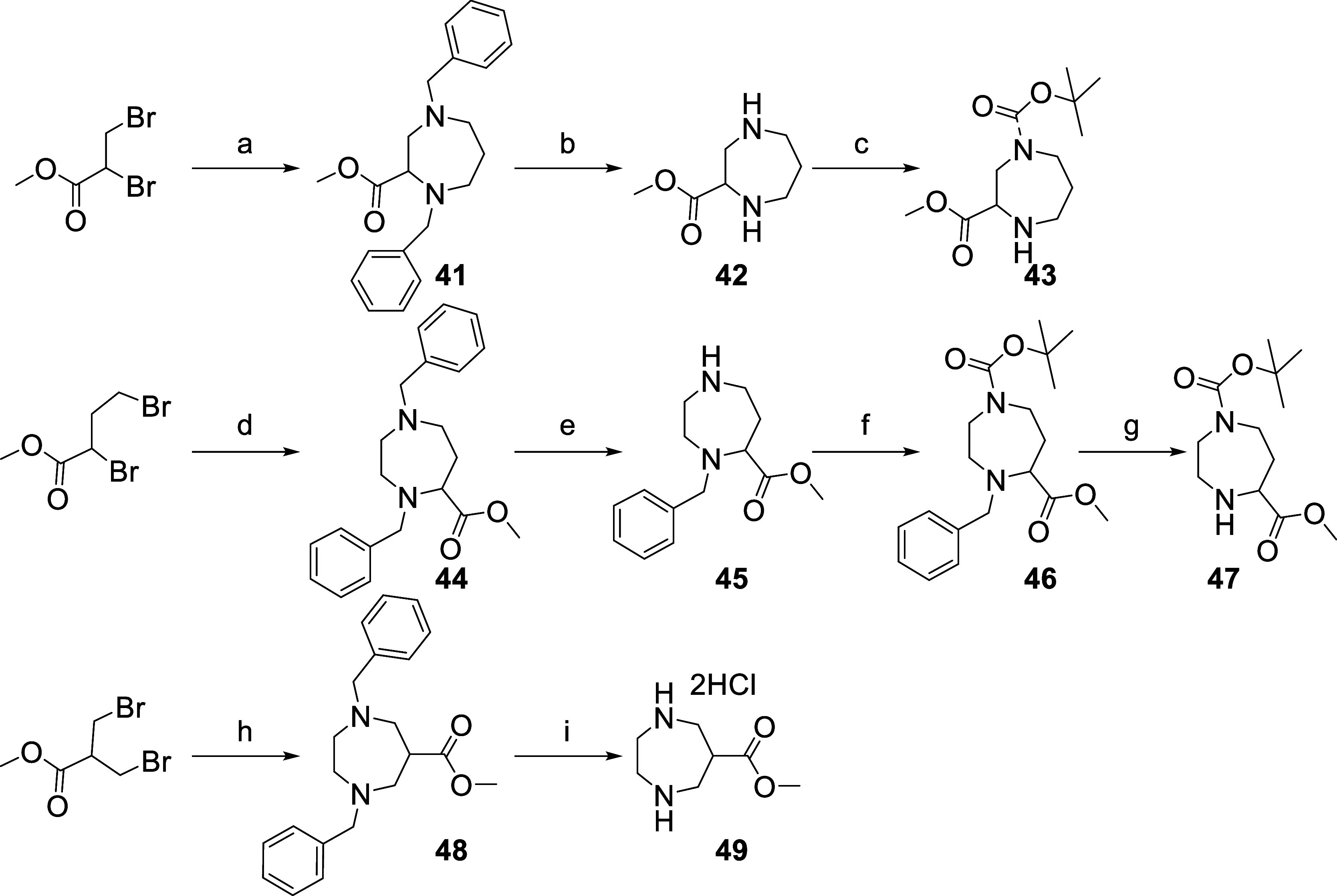
Preparation of the Three Different Diazepane Scaffolds Bearing a Protected Carboxylic Acid Functionality Reagents and conditions: (a) *N*-N-dibenzylpropane-1,3-diamine, K_3_PO_4_; MeCN, 55 °C, 16 h; (b) H_2_ (1 bar), Pd/C, MeOH, rt, 1 h 30; (c) Boc_2_O, NEt_3_, DCM, RT, 1 h 30; (d) *N*-*N*-dibenzylethane-1,2-diamine, K_3_PO_4_, MeCN, 55 °C, 16 h; (e) (i) ACE-Cl, DCE, 85 °C, 1 h; (ii) MeOH, 65 °C, 1 h; (f) Boc_2_O, NEt_3_, DCM, rt, 16 h; (g) H_2_ (1 bar), Pd/C, MeOH, RT, 2 h; (h) *N*-*N*-dibenzylethane-1,2-diamine, K_3_PO_4_, MeCN, 55 °C, 16 h; (i) H_2_ (1 bar), Pd/C, MeOH, RT, 3 h 30.

To confirm this hypothesis, we embarked on the synthesis of the five isomers. The required diazepane scaffolds **43**, **47**, and **49** were prepared via a cyclization reaction between a dibromo derivative and a benzyl protected bis amine to give intermediate **41**, **44**, and **48**, as described in Scheme 1. The two benzyl protecting groups present in compound **41** were then removed by hydrogenation, and the resulting diamine **42** was selectively Boc protected by treatment with Boc_2_O and NEt_3_ in DCM to give scaffold **43**. The Boc-protected scaffold **47** was obtained by selective mono debenzylation of compound **44** using 1-chloroethylchloroformate followed by Boc protection and removal of the second benzyl group. Scaffold **49** was obtained by complete debenzylation of compound **48**.

**Scheme 2:**
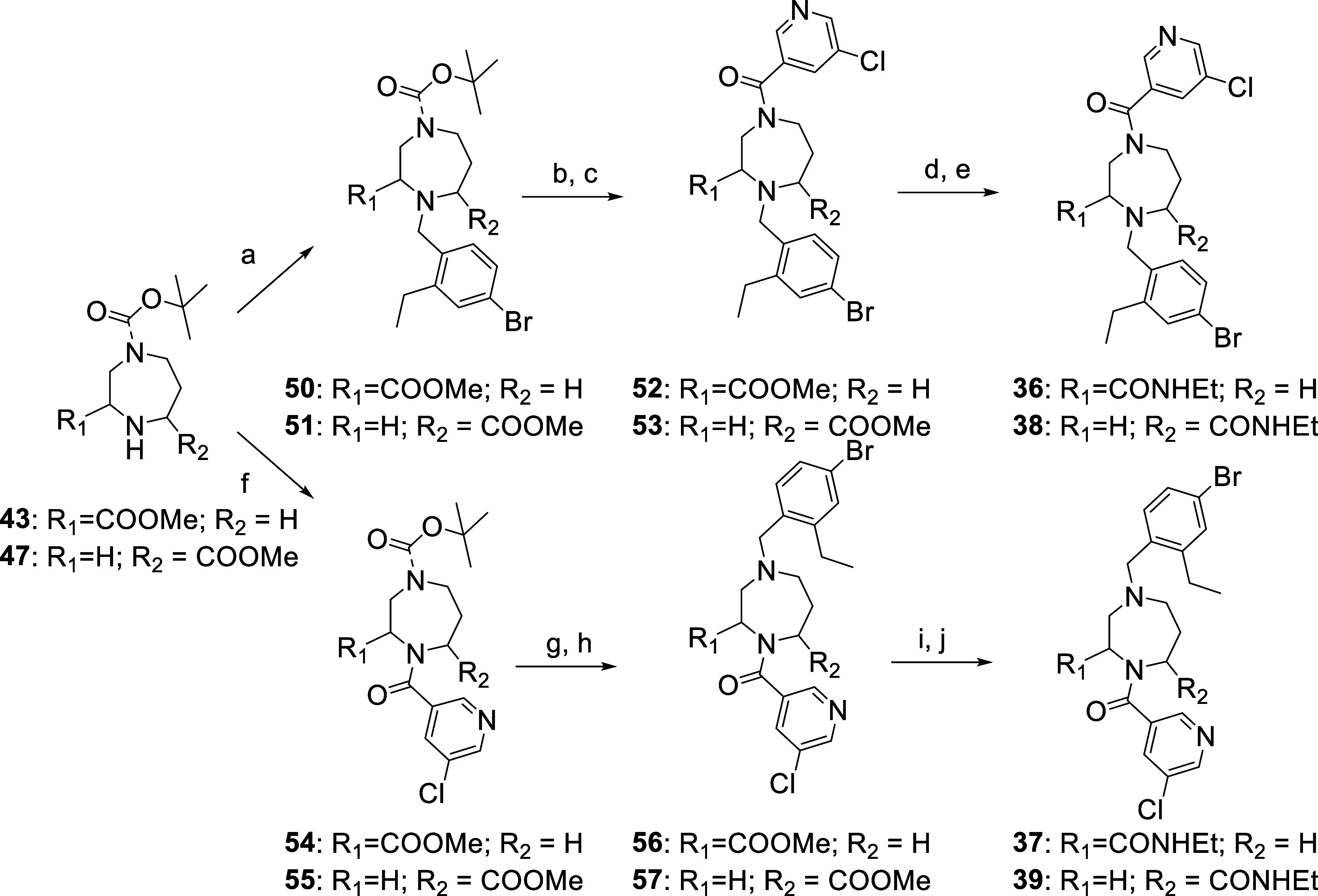
Synthesis of the Tri-Substituted Diazepane Analogues **36**, **37**, **38** and **39** Reagents and conditions: (a) 4-bromo-2-ethylbenzaldehyde, NaBH(OAc)_3_, DIPEA, DCM; (b) HCl (4 M in dioxane), DCM; (c) 5-chloronicotinic acid, HATU, DIPEA, DMF; (d) LiOH·H_2_O, THF/H_2_O; (e) ethylamine, HATU, DIPEA, DMF; (f) 5-chloronicotinic acid, HATU, DIPEA, DMF; (g) HCl (4 M in dioxane), DCM; (h) 4-bromo-2-ethylbenzaldehyde, NaBH(OAc)_3_, DIPEA DCM; (i) LiOH·H_2_O, THF/H_2_O; (j) ethylamine, HATU, DIPEA, DMF.

**Scheme 3:**
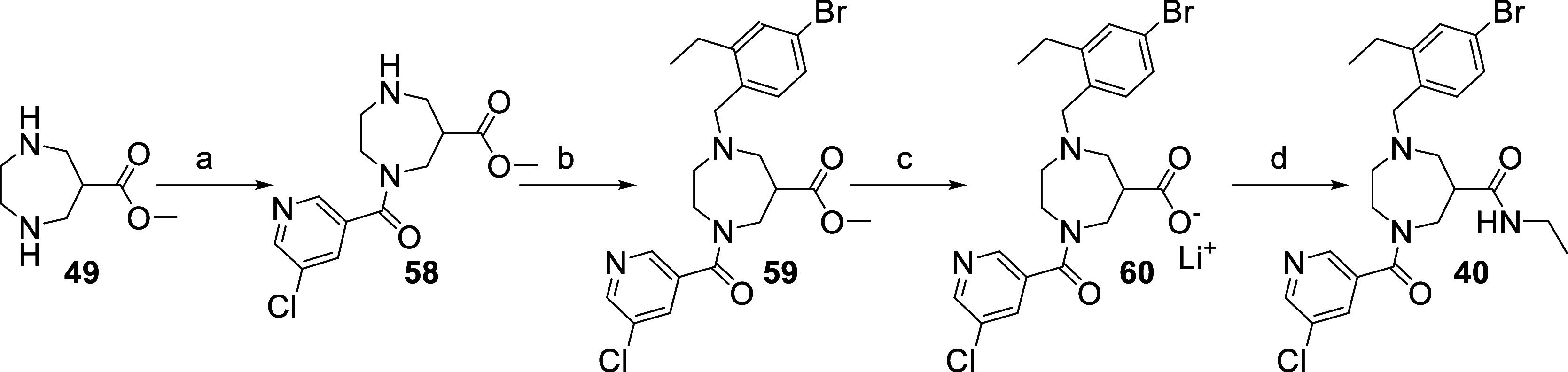
Synthesis of the Tri-Substituted Diazepane Analogue **40** Reagents and conditions: (a) 5-chloronicotinic acid, HATU, DIPEA, DMF; (b) 4-bromo-2-ethylbenzaldehyde, NaBH(OAc)_3_, DIPEA, DCM; (c) LiOH·H_2_O, THF/H_2_O; (d) ethylamine, HATU, DIPEA, DMF.

Having the three scaffolds in hand, we initiated the synthesis of target compounds (Schemes 2 and 3). Compound **36** was prepared starting from scaffold **43**, by first performing a reductive amination with 4-bromo-2-ethylbenzaldehyde in the presence of sodium triacetoxyborohydride to give compound **50**. The Boc group was then removed by treatment with HCl (4 M in dioxane), and the resulting amine was coupled with 5-chloronicotinic acid using HATU as a coupling reagent and DIPEA as a base. The methyl ester was then removed by saponification with LiOH, and treatment with ethyl amine in the presence of HATU leads to compound **36** (Scheme 2). The synthesis of compound **37** started from the same scaffold **43** but this time first the amide coupling with 5-chloronicotic acid was performed to give compound **54** followed by Boc deprotection and reductive amination with 4-bromo-2-ethylbenzaldehyde to give compound **56**. Saponification and amide coupling lead then to the target compound **37** (Scheme 2).

Compounds **38** and **39** (Scheme 2) were synthesized using the same succession of reaction, respectively, but starting from scaffold **47**.

Starting from scaffold **49**, compound **40** (Scheme 3) was prepared by first performing a amide coupling to introduce the chloronicotic acid moiety followed by a reductive amination with 4-bromo-2-ethylbenzaldehyde, then saponification of the methyl ester and finally formation of the ethylamide via amide coupling.

The five tri-substituted diazepanes **36**, **37**, **38**, **39**, and **40** were tested for the inhibitory activity against Mpro using the previously describe FRET assay (Table 1). The results are in perfect agreement with the MD simulation results. Compound **36** for which no docking pose could be identified has a very poor inhibitory activity. Conversely, compounds **37** and **38**, projected as best isomers during the MD simulation, are also the most potent inhibitors identified in this series with an IC_50_ of 660 and 280 nM, respectively.

**Table 1:** Inhibitory Activity of the Tri-Substituted Diazepane Analogues

compound	**36**	**37**	**38**	**39**	**40**
IC_50_ [μM]	8.8	0.66	0.28	2.8	1.9

compound	**37a**	**37b**	**38a**	**38b**	
IC_50_ [μM]	0.33	57	0.16	46	

**Figure 8:**
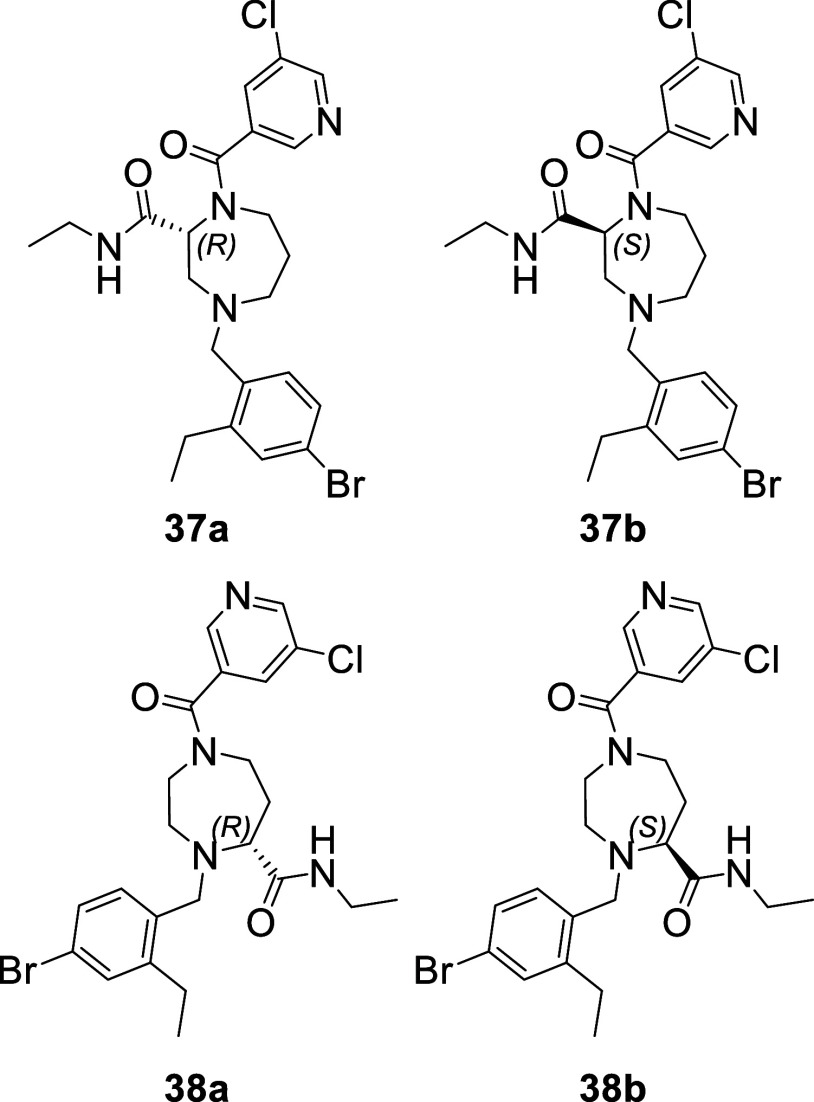
Structure of compounds **37a**, **37b** and **38a** and **38b** the enantiomers of compound **37** and **38** respectively.

**Figure 9:**
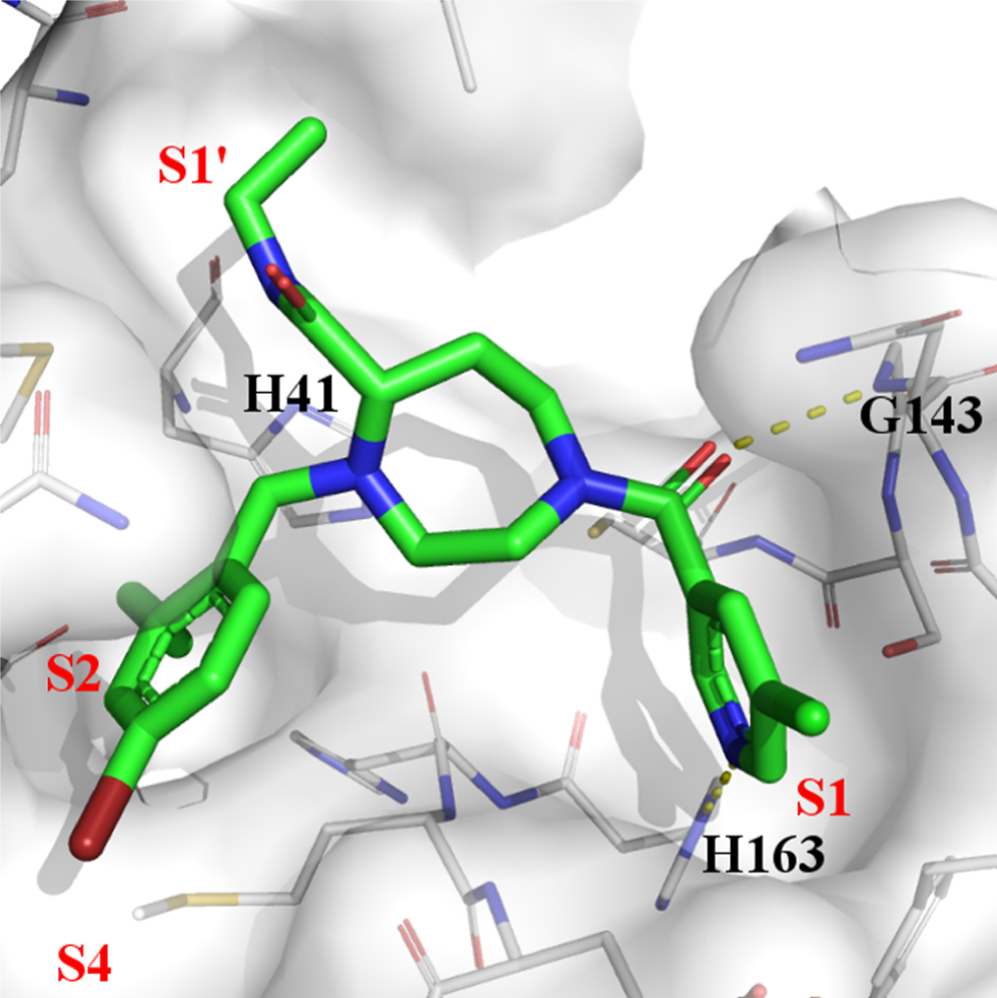
Binding mode of compound **38a** (PDB code: 9HAJ) in complex with Mpro. The stereochemistry at the chiral center is defined by the X-ray structure as (R).

Except compound **40**, all these new analogues contain a chiral center and were initially measured as racemic mixtures. Therefore, to assess the impact of the chiral center on the inhibitory activity, the pure enantiomers of compounds **37** (Figure 8, compounds **37a** and **37b**) and **38** (Figure 8, compounds **38a** and **38b**) were obtained by chiral separation and their inhibitory activity was assessed in the enzymatic FRET assay (Table 1). Clearly in both cases, one enantiomer is much more potent than the other. For both compounds (**37** and **38**), the (R) configuration of the chiral center was determined by X-ray crystallography as the most potent enantiomer bound into the active site of Mpro (Figures 9 and S1). The S configuration did not fit into the electron density map obtained by the crystallographic experiment.

The X-ray structure of compound **38a** bound into the active site of Mpro (Figure 9) confirmed our hypothesis. The newly introduced ethylcarboxamide side chain fits nicely in the S1′ pocket. The ethyl is mainly involved in van der Waals interaction while the amide moiety might be involved in water-mediated interactions. The *m*-chloropyridine moiety sits in the S1 pocket making a direct H-bond interaction between the N of the pyridine ring and H163. The 2-ethyl-3-bromophenyl is filling the hydrophobic S2 pocket while the bromine atom points toward S4. The resulting improvement of the inhibitory activity is in agreement with the MD simulation described above.

**Scheme 4:**
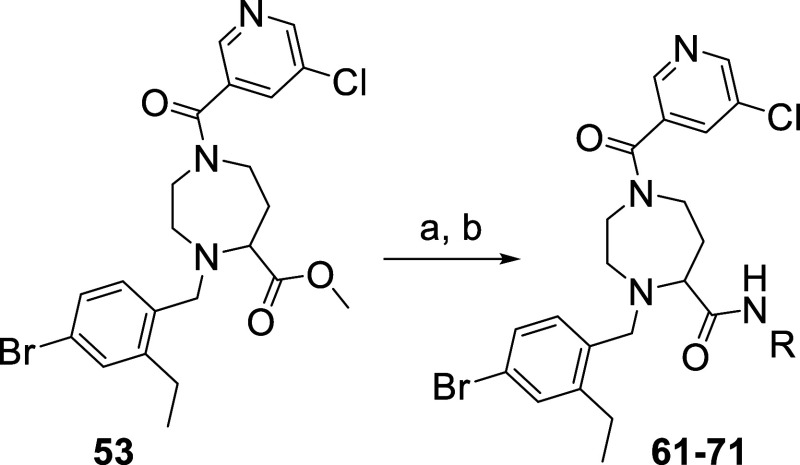
Synthesis of a Small Library of Amides at the Newly Introduced Exit Vector Reagents and conditions: (a) LiOH·H_2_O, THF/H_2_O; (b) RNH_2_, HATU, DIPEA, DMF.

Starting from compound **53**, we probed the S1′ pocket by synthesizing a diverse set of amides at the newly introduced exit vector. As shown in Scheme 4, we used the same conditions for ester hydrolysis and amide coupling as previously described for compound **38**. The newly synthesized compounds contain linear (**62**), and cyclic (**61**, **63**) alkyl amide, phenyl (**65**), and benzyl (**64**) amides side chain as well as primary (**61**, **62**, **63**, **64**, **65**) and secondary amides (**66**, **67**, **68**, **69**, **70**, **71**). The inhibitory activity of this set of amides is reported in Table 2. The data indicate that the S1′ pocket can best accommodate small residues such as ethyl **38**, cyclopropylmethyl **61** isopropyl **62**, and pyrrolidine **68** moiety. Increasing size is less favorable (see compound **63**, **64**, **65**). It appears also that methylation of the amide nitrogen leads to a slight decrease in inhibitory activity (compare compound **38** and **66** as well as **63** and **70**).

**Table 2:**
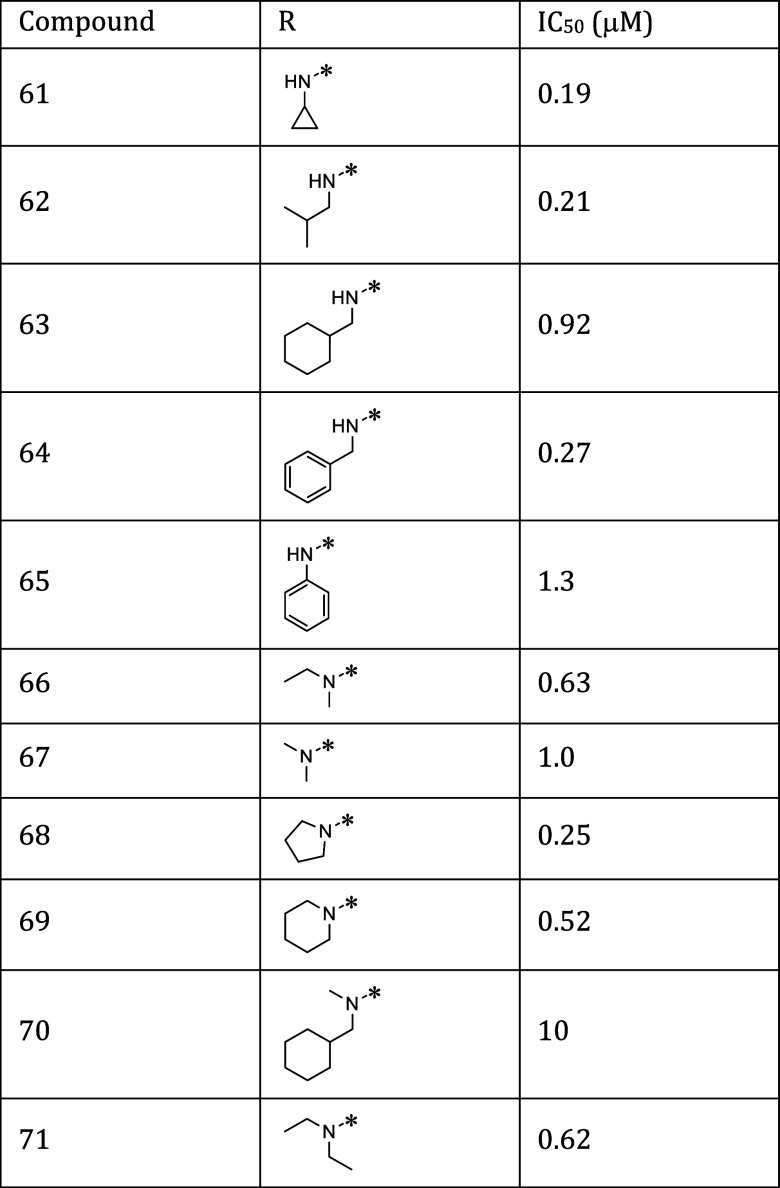
Inhibitory Activity of a Diverse Set of Amides at the Newly Introduced Exit Vector

### Optimization of Interactions in the S1 Pocket

As shown in Figures 2 and 9, the 3-chloropyridine moiety present in all inhibitors described so far in this study inserts deeply into the S1 pocket making hydrophobic interaction and a key H bond with His163. These combined interactions are essential for the inhibitory activity, and we therefore used this information to optimize the binding in the S1 pocket.

**Figure 10:**
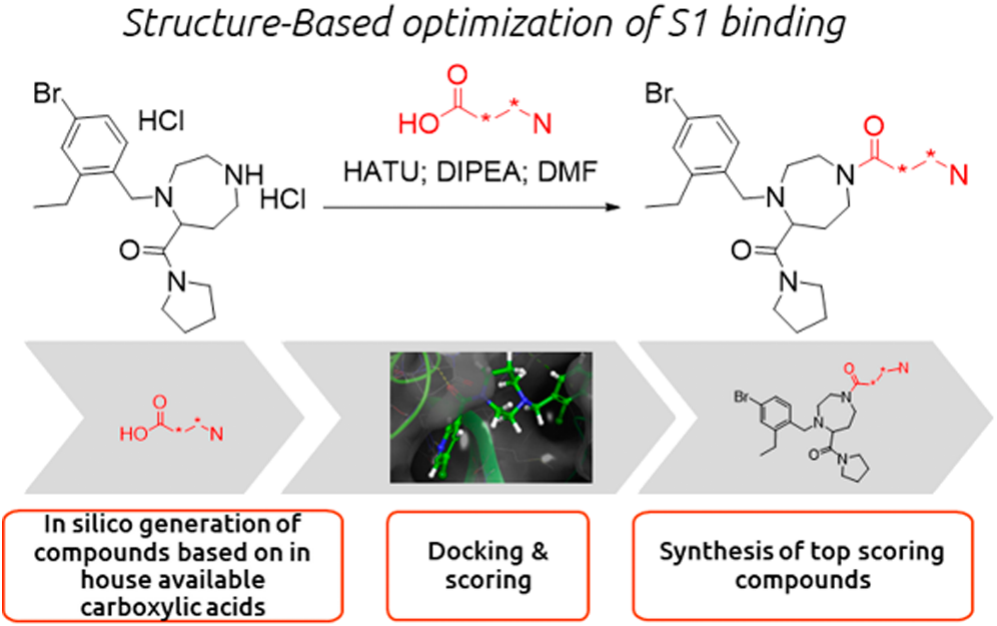
Optimization of the binding into the S1 pocket was achieved by using the following workflow: in silico generation of a virtual library based of amide coupling reaction using in-house building blocks; docking of the virtual compounds into the active site of the enzyme; consensus scoring; synthesis of the best scoring compounds and assessment of the inhibitory activity in a enzymatic FRET assay.

As for the optimization in the S2 pocket, we combined structure-based design with parallel synthesis. To do so we enumerated a virtual library of 330 molecules based on amide coupling reaction using a selected set of our in-house stock of carboxylic acid containing a nitrogen acceptor at a distance of 3 atoms from the amide carbonyl (Figure 10). All enumerated compounds contained the 2-ethyl-3-bromobenzyl moiety and the pyrrolidine amide at the newly introduced exit vector. These compounds were docked into the Mpro–compound **38a** complex. After visual inspection, 213 docking poses were retained, and WM/MM binding free energy and the acceptor strength (as described in the Experimental Section) of the nitrogen in position 33 were calculated. Compounds for which the WM/MM Δ-*G* bind <−32 kcal/mol and the acceptor strength >0.77 were selected.

**Scheme 5:**
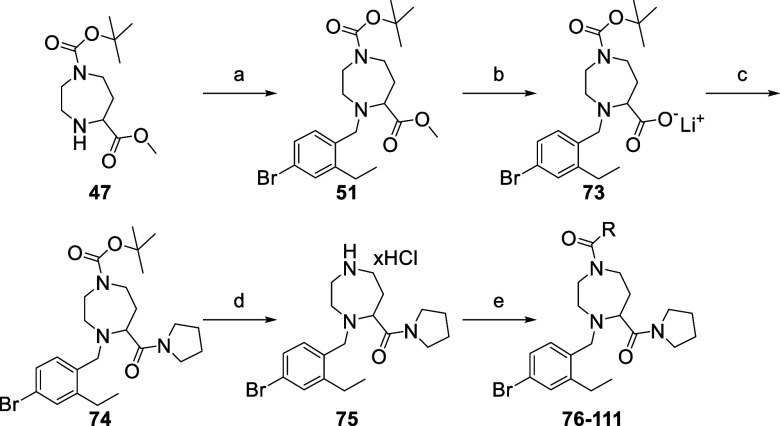
Synthesis of an Amide Library for the Exploration of the S1 Pocket Reagents and conditions: (a) 4-bromo-2-ethylbenzaldehyde, NaBH(OAc)_3_, DIPEA, DCM; (b) LiOH·H_2_O, THF/H_2_O; (c) pyrrolidine, HATU, DIPEA, DMF; (d) HCl 4 M in dioxane; (e) carboxylic acid, HATU, DIPEA, DMF.

In total, 36 molecules were prioritized for synthesis by HTMC. The synthesis of the required scaffold **75** and the subsequent library (**76–111**) is described in Scheme 5. The synthesis started with the introduction of the 4-bromo-2-ethylbenzyl moiety on scaffold **47** via a reductive amination. After saponification of the methyl ester, the pyrrolidine-carboxamide was formed using pyrrolidine, DIPEA, and HATU as coupling reagent. Finally, the Boc protecting group was removed using HCl (4 M in dioxane) and the resulting scaffold was engaged in the amide library synthesis with the **36** selected carboxylic acids using the same coupling condition as previously.

The inhibitory activities of the newly synthesized compounds were measured in the FRET assay (see Figure 13). Out of the 36 compounds selected by computational methods, 33 displayed a measurable inhibitory activity. The highest gain in activity was obtained by introducing a bicyclic system such as the thienopyridine **105** and the azaindole **106** having an IC_50_ of 0.04 and 0.06 μM, respectively. Other bicyclic systems are tolerated, such as the isoquinoline or naphtiridine ring, but the position of the second N in the naphtiridine ring system plays an important role as shown by compound **107**, **108**, and **109**. In addition, the 5–5 ring system present in compound **100** is slightly better than the 6–6 ring system present in compound **101**.

**Figure 11:**
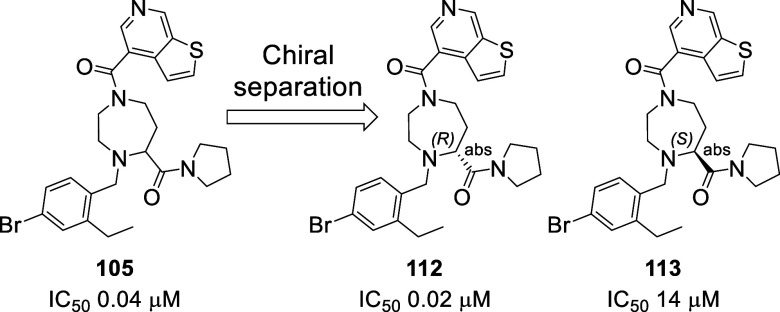
Chemical structures and inhibitory activity of compounds **112**, **113**.

**Figure 12:**
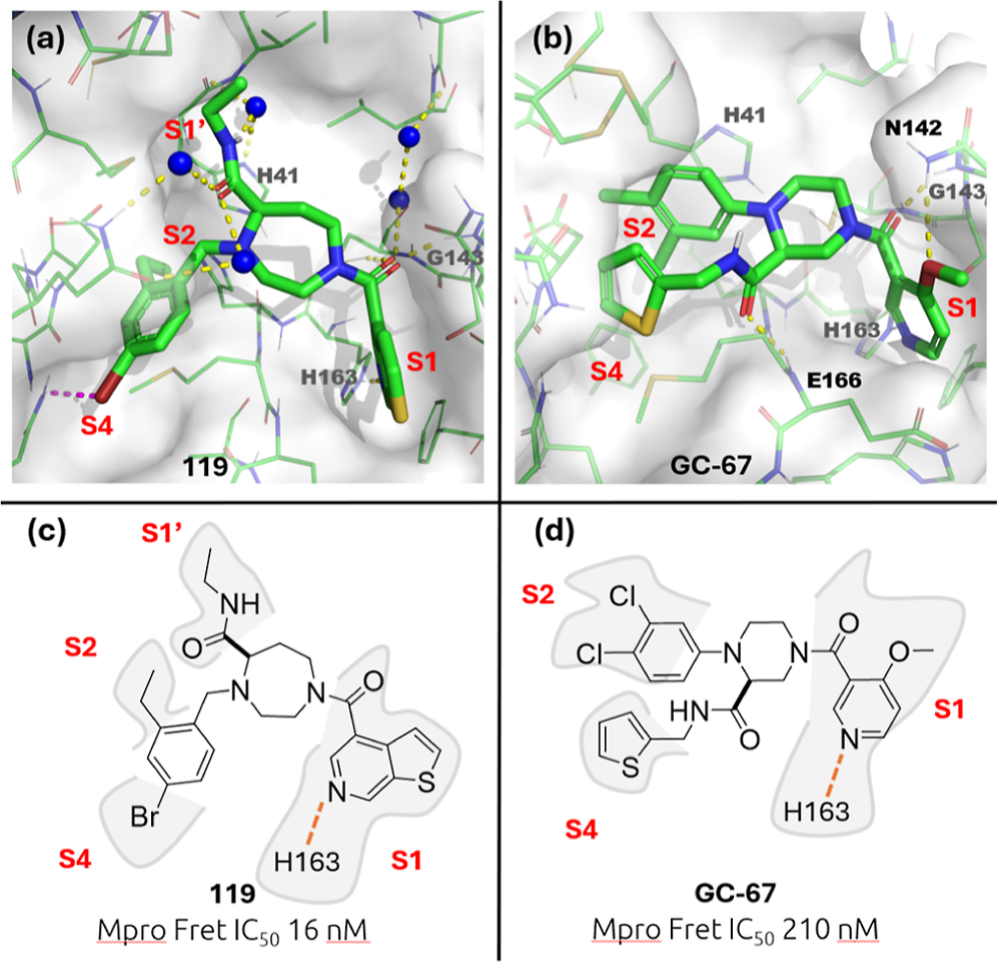
Binding mode of compound **119** (PDB code: 9HAK) and **GC-67** (PDB code: 8Q71) in complex with Mpro and 2D representation with enzymatic activities. (a) View of compound **119** in the binding pocket. Blue spheres represent water molecules, and dashed lines represent the interactions. (b) View of **GC-67** in the binding pocket. Dashed lines represent the interactions. (c) 2D view of compound **119** with respective location of each moiety in the active site and its enzymatic activity. (d) 2D view of **GC-67** with respective location of each moiety in the active site and its enzymatic activity.

**Figure 13:**
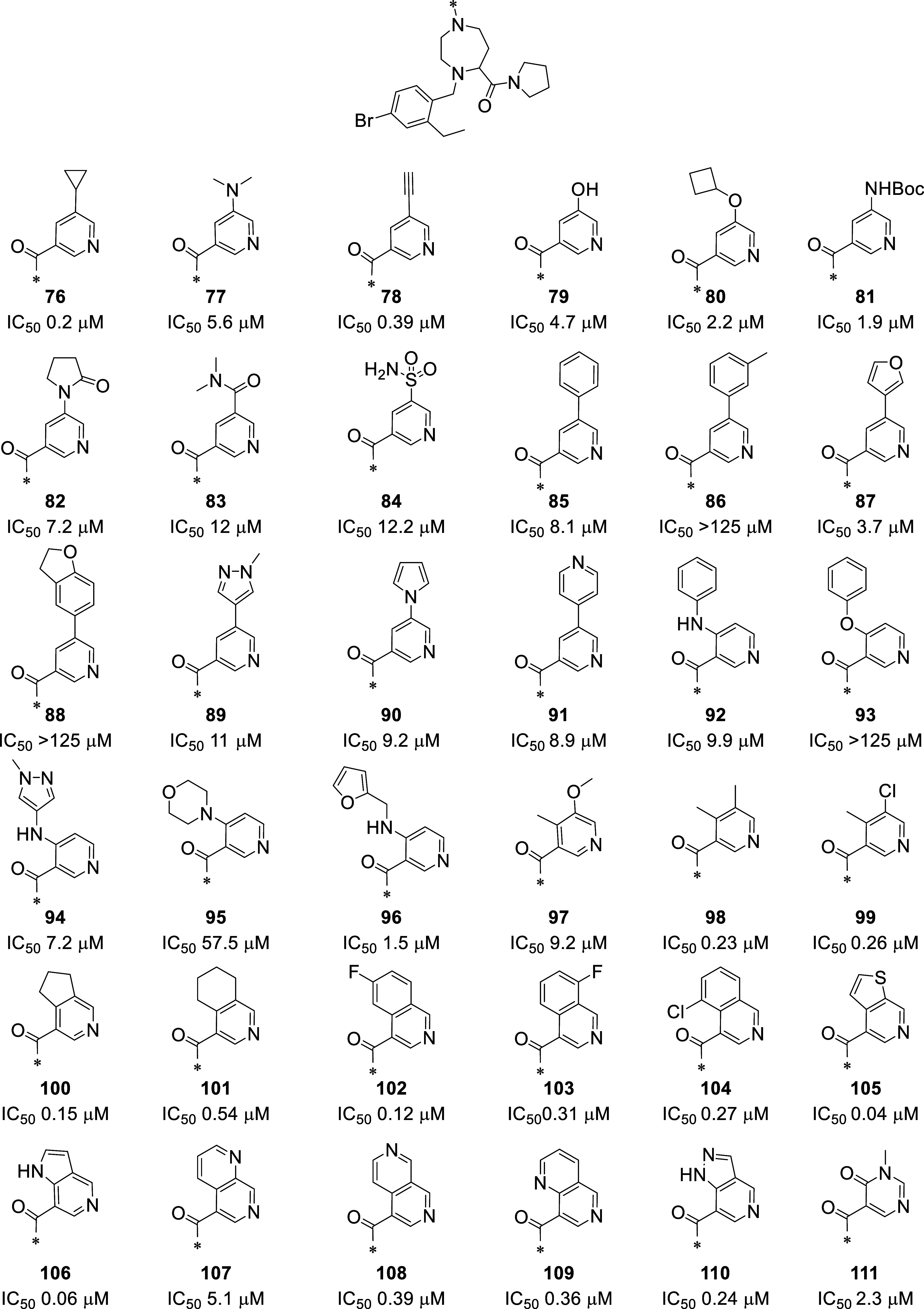
Chemical structures and inhibitory activity of compounds **76–111**.

The establishment of the SAR through the combination of HTMC and SBDD was straightforward and allowed for the rapid identification of potent compounds. However, we also identified limitations and areas for improvement. In terms of SAR, for example, it is clear that synthesizing only a limited number of compounds resulted in a restricted coverage of the possible chemical space. A solution to address this issue would be to use a predefined set of a limited number of highly diverse building blocks to broadly explore via library synthesis each exit vector.

Regarding the structure-based design of the compounds, it became clear that docking predictions alone can be misleading as compounds can end up inactive while producing reasonable docking poses. To improve the reliability of these predictions, additional computational approaches were incorporated. These included WaterMap analysis to assess the energy and spatial distribution of water molecules within the binding site and hydrogen bond strength calculations that revealed a highly potent moiety in the S1 pocket, or MD simulations, which guided the introduction of a new exit vector.

### Lead Series Characterization

To evaluate the lead like properties of this newly discovered diazepane series, we prepared four enantiomerically pure representative bearing the key SAR features identified so far and we assessed physicochemical properties such as log *D* and solubility. We also compared the selectivity of the compounds for Mpro versus the closely related cysteine protease cathepsin L. In addition, we determined early DMPK parameters such as metabolic stability and permeability.

**Scheme 6:**
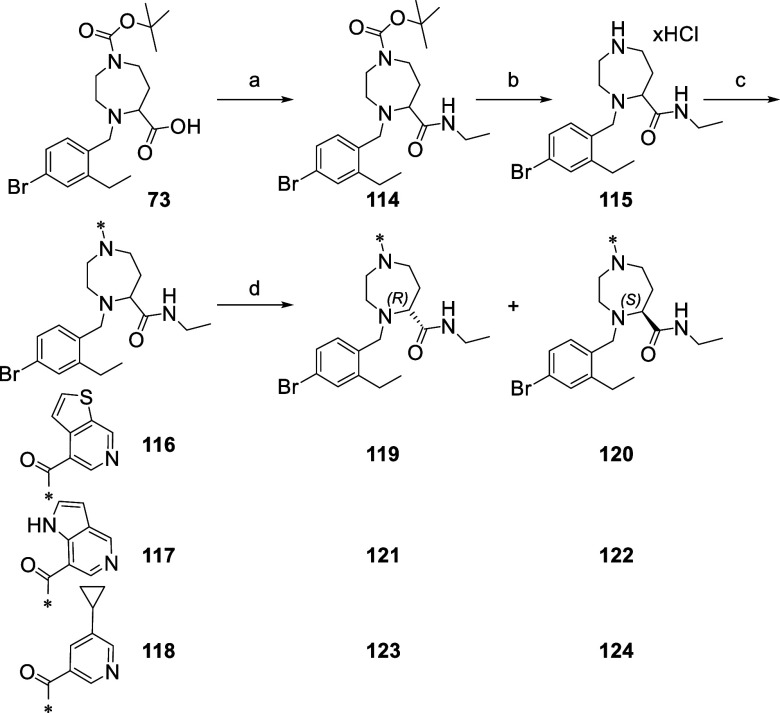
Synthesis of Compounds for Lead Characterization Reagents and conditions: (a) ethylamine, HATU, DIPEA, DMF; (b) HCl 4 M in dioxane LiOH·H_2_O, THF/H_2_O; (c) carboxylic acid, HATU, DIPEA, DMF; (d) chiral separation.

As shown in Figure 11, compound **112** was obtained by chiral separation of compound **105** synthesized in the previous library. Regarding compounds **119**, **121**, and **123**, they were obtained starting from scaffold **73**, as described in Scheme 6 using the previously established synthetic route.

The properties of the four enantiomerically pure compounds **112**, **119**, **121**, and **123** are summarized in Table 3. The inhibitory activities are ranging from 16 to 74 nM with a very high selectivity for Mpro versus cathepsin L. All the compounds have an acceptable solubility in FASSIF and at pH 6.4. Unsurprisingly, the metabolic stability in human microsome is rather low. This is probably due to the presence of the two amide functionality. This hypothesis is confirmed by the fact that modifying either amide has an impact on the metabolic stability. For instance, a switch from the ethyl amide present in compound **119** to the pyrrolidine amide in compound **112** increases the metabolic stability by a factor two. Similarly, compounds **119**, **121**, and **123** differ only by the nature of the heterocycle attached to the diazepane ring and as indicated by the value of the respective metabolic stability, the nature of the heterocycle impacts the metabolic stability. Regarding permeability, it appears that compounds **119**, **112**, and **121** are substrates of efflux transporters as indicated by the high efflux ratio in the MDR1 assay but also here the nature of the heterocycle attached to the diazepane ring can modulate this property (see compound **123**).

**Table 3:**
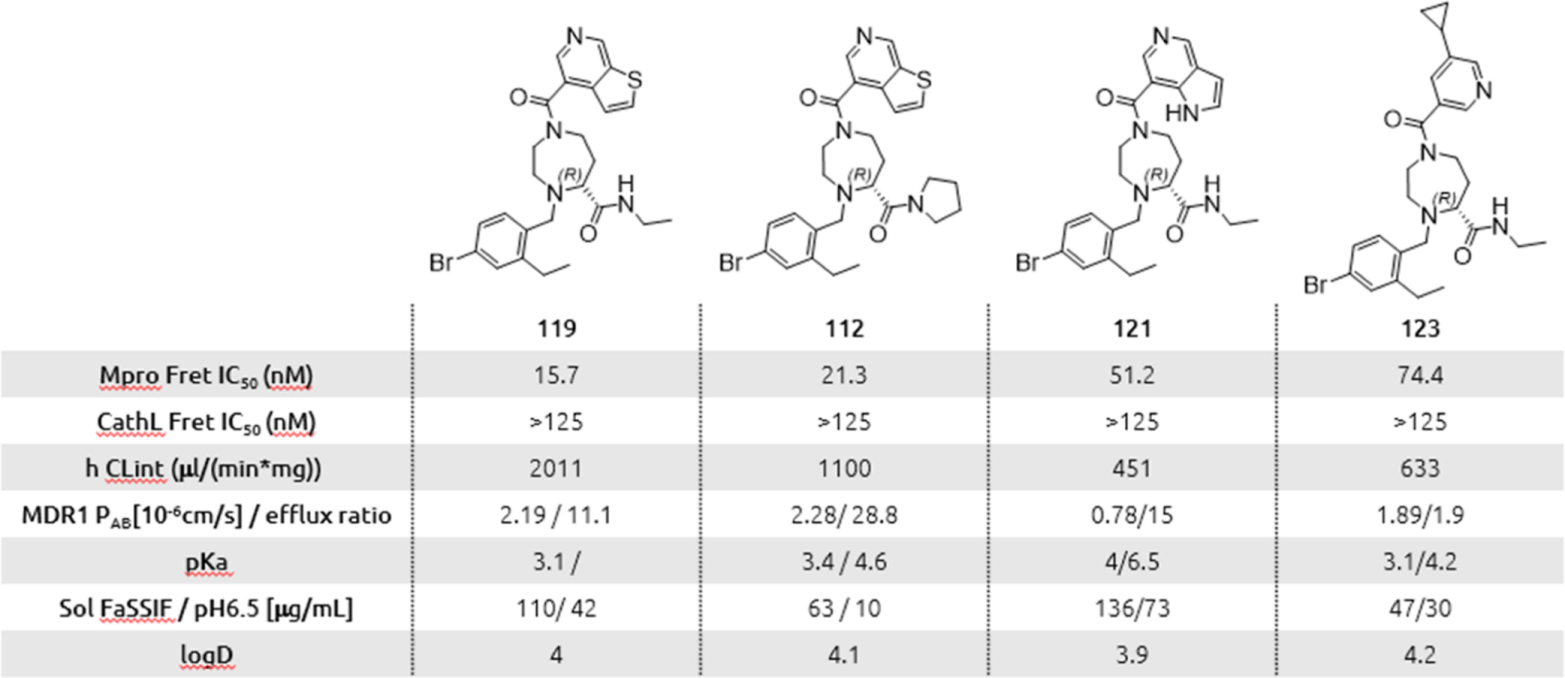
Properties of the 4 Enantiomerically Pure Compounds **112**, **119**, **121**, and **123**

The crystal structure of Mpro in complex with compound **119** (IC_50_: 16 nM) was obtained. As depicted in Figure 12a, we can observe an interesting network of water-mediated interactions. In particular, the ethylcarboxamide engages in four water-mediated interactions with three water molecules. The carbonyl group of the ethylcarboxamide forms two water-mediated hydrogen bonds with two water molecules and the side chain amide group of Gln189. Additionally, the NH group makes two water-mediated hydrogen bond interactions with one water molecule, the imidazole side chain, and the backbone carbonyl of His41.

The rich water-mediated interactions surrounding the ethyl-carboxamide, along with the presence of the ethyl moiety in the lipophilic S1′ pocket, likely account for the observed increase in binding affinity when introduced through a new exit vector. The ethyl group of 4-bromo-2-ethylbenzylamine occupies the hydrophobic S2 pocket, while the bromine atom is oriented toward the S4 pocket, forming a halogen bond with the Gln189 side chain. An unstable water molecule (Figure 3) is located precisely where the bromine atom (Figure 9) resides, suggesting that the displacement of this water molecule may have contributed to an increase in binding affinity. The thienopyridine carboxamide, identified during S1 pocket optimization, engages in a water-mediated interaction while also forming hydrogen bonds with Gly143 and Cys145 via its carbonyl group. Additionally, it establishes a hydrogen bond with His163, similar to what was observed with the 3-chloropyridine carboxamide. We hypothesize that the hydrogen bond strength is enhanced as the acceptor strength value increased from 0.78 in the 3-chloropyridine to 0.80 in the thienopyridine. Furthermore, the thienopyridine occupies the S1 pocket more effectively, leading to increased van der Waals interactions compared to the 3-chloropyridine. These combined factors likely contribute to the observed increase in binding affinity of our lead compound **119**.

### Comparison of the Newly Identified Diazepane Series (Compound **119** as a Reference) with the Previously Described Piperazine Series (Compound **GC-67** as a Reference)

The diazepane and piperazine series are very similar from a chemical structure point of view. As depicted in Figure 12c,d, both are built around a bis-nitrogen-containing cyclic scaffold, differing in size by just a single carbon atom. The two series also share one common feature on one exit vector namely an amide at one of the cyclic nitrogens containing a substituted 3-pyridine ring. The second nitrogen bears a benzylic substituent in the case of the diazepane series (Figure 12c), whereas in the piperazine series (Figure 12d), the second nitrogen is arylated. Furthermore, both scaffolds feature a third exit vector, which is an amide: an alkyl amide in the case of the diazepane and a thiophen-2-ylmethyl amide in the case of the piperazine.

This close chemical structural similarity is only partially translated to the binding mode within the active site of the enzyme. The 3-pyridine derived heterocycle (Figure 12a,b) is a key anchor point for both series via a hydrogen bond between the nitrogen of the pyridine and H163 present in the S1 pocket. For the diazepane (Figure 12a), the 2-ethyl-3-bromobenzyl substituent at the second nitrogen distributes the ethyl group into the S2 pocket and the bromine into S4, while in the case of the piperazine (Figure 12b), the dichlorophenyl group exclusively interacts with the S2 pocket via π–π stacking interactions involving the imidazole side chain of H41.

The most significant and unexpected divergence occurs at the third exit vector. In the diazepane series (Figure 12a), this moiety engages with the S1′ pocket, whereas in the piperazine series (Figure 12b), the thiophen-2-ylmethyl extends into the S4 pocket. This enhanced spatial distribution of the diazepane scaffold results in broader interactions across the S1, S1′, S2, and S4 pockets. As a consequence, compounds in the diazepane series demonstrate up to 10-fold higher inhibitory activity in enzymatic assays compared to the piperazine series. For example, compound **119** exhibits an IC_50_ of 16 nM, whereas the best piperazine derivative GC-78-HCl has an IC_50_ of 170 nM.

Regarding the physicochemical properties, a slight advantage goes to the diazepane series as the benzylic amine moiety may allow for better solubility compared to the *N*-dichlorophenyl moiety.

From an ADME perspective, the structural similarity of the two series translates into similar drug-like properties. Both series suffer from poor metabolic stability, which is partially due to the presence of two amides in both series, as well as the benzylic moiety in the diazepane series and the OMe substituent present in the piperazine compound **GC-67**. The variation in cellular permeability is another aspect that the two series have in common. In the case of the piperazine series, the initial lead compound suffered from limited cell permeability, which translated into poor antiviral activity. The absence of antiviral activity was addressed during lead optimization by decreasing the polarity of the compounds. For the diazepane series, our initial data (Table 3) suggest that some compounds in this series are PGP substrates, a property that would limit the cellular absorption of the compounds and, therefore, impact antiviral activity. However, as highlighted in Table 3, in the diazepane series properties such as metabolic stability, drug uptake, and efflux can be positively modulated by structural modifications.

In conclusion, despite the close structural similarity between the diazepane and piperazine series, the binding mode analysis, enabled by X-ray data, clearly distinguishes the two. The substitution pattern around the diazepane ring allows interactions with four binding pockets within the enzyme’s active site, whereas the piperazine series, with a similar three-exit-vector distribution, is limited to three pockets. This additional interaction results in significantly higher enzymatic inhibitory activity. In terms of drug-like properties, both series exhibit similar characteristics. However, the unexpected and advantageous binding behavior of the diazepane scaffold, combined with the potential to modulate its drug-like properties through structural modifications, provided the foundation for its selection as the lead series.

The results described above are the outcome of a close and effective collaboration between computational and medicinal chemistry. A pragmatic approach to molecular design, informed by computer simulations, was complemented by the skilled selection of compounds by a medicinal chemist. This synergy created a highly efficient workflow, capable of rapidly transforming a hit into a lead series, ready for lead optimization.

## Conclusions

Starting from a computationally identified hit based on a diazepane scaffold, we rapidly transformed this initial structure into a potent and selective lead series using state-of-the-art structure-based design and HTMC. By optimizing key areas of the binding site, including the S1, S2, S1′, and S4 pockets, and, by identifying a new exit vector on the diazepane core, we significantly enhanced the binding affinity of the diazepane series. Crystallographic analysis of compound **119** bound to Mpro provided a rational explanation for the increased binding affinity. Comparison of the binding mode of compound **119** with the binding mode of **GC-67**, a representative of a closely related Mpro inhibitor series built on a piperazine scaffold reveals an unexpected difference in favor of the diazepane ring leading to a 10-fold higher enzymatic inhibitory activity. Additionally, advanced characterization of four potent compounds (**119**, **112**, **121**, and **123**) are indicating that lead properties, such as human metabolic stability, drug uptake and efflux, can be modulated through targeted structural modifications. As a result, this new series of Mpro inhibitors is well-positioned to be selected as a lead candidate for entry into the lead optimization phase.

## Experimental Section

Purity of all final compounds is ≥95%.

Commercially available chemicals and solvents were used as received from the suppliers. All reactions were either performed in a single-neck round-bottom flask or in an 8.5 mL round-bottomed vial from Infochroma AG under an atmosphere of nitrogen or argon.

For the purification by flash chromatography, CombiFlash Rf+ was used. Pre-packed columns from the RediSep company were used. The following sizes were available: 4, 12, 24, 40, 80, and 120 g. Isolute was used to absorb the crude material which was then loaded on the column. Elution was performed with EtOAc, heptane, DCM, MeOH, or a mixture thereof.

Analytical LC–MS: the HPLC/MS analyses were performed on a Waters Acquity iClass instrument equipped with a Waters binary solvent manager, a Waters sample manager FL, a Waters column manager, and a Waters photodiode array detector connected to a Thermo Fisher MSQ Plus mass spectrometer. Data acquisition was done using the Dionex Chromeleon 6.8 software package.

Acidic condition: chromatographic separation was achieved on an Agilent Zorbax RRHD SB-AQ column (50 × 2.1 mm ID, 1.8 μm) at 40 °C with a flow rate of 0.8 mL/min. Mobile phases consisted of water/0.04% TFA (phase A) and acetonitrile (phase B). A linear gradient from 5 to 95% of phase B within 1.2 min, followed by isocratic elution with 95% of phase B for 0.7 min, was applied.

Basic condition: chromatographic separation was achieved on a Waters BEH C18 column (50 × 2.1 mm ID, 2.5 μm) at 40 °C with a flow rate of 0.8 mL/min. Mobile phases consisted of water/0.05% ammonia (phase A) and acetonitrile (phase B). A linear gradient from 5 to 95% of phase B within 1.2 min, followed by isocratic elution with 95% of phase B for 0.7 min, was applied.

Preparative purifications were performed on an Agilent HPLC system, equipped with a Gilson GX-281 autosampler, Agilent pump G7161B, Thermo Fisher ISQ-EM mass detector system, and an Agilent G7115A photodiode array detector, using a Waters Xbridge C18 (75 × 30 mm ID, 10 μm) or an Agilent Zorbax SB-AQ column (75 × 30 mm ID, 5 μm), with a linear gradient of water/0.5% formic acid (A) and MeCN (B) (acidic conditions) or water/0.5% ammonia (A) and MeCN (B) (basic conditions) at a flow rate of 75 mL/min. Detection UV/vis and/or MS. Retention time (*t*_r_) is given in min; molecular weight obtained from the mass spectrum is given in g/mol. Data acquisition was done using the Thermo Fisher Chromeleon 7.2 software package.

Purity of all final compounds was checked by an additional LC–MS analysis (LC-HRMS) using an Acquity UPLC system (Waters Corporation, Milford, MA) which include a binary solvent manager, a sample manager, PDA detector, and a column oven coupled to a q-TOF SYNAPT G2 mass spectrometer. Mobile phase A consisted of water with 0.05% formic acid while mobile phase B was acetonitrile with 0.045% formic acid. 0.2 μL of compound management DMSO stock solution was loaded onto a Acquity C18 CSH column form Waters (particle size: 1.7 μm; 2.1 mm i.d. × 50 mm, Waters Corporation, Milford, MA) at 60 °C. Initial gradient conditions were set at 2% mobile phase B, increasing to 95% at 1.5 min, held for 0.4 min, and then returned to the initial conditions. Flow: 1.0 mL/min. MS detection was carried out using a q-TOF SYNAPT G2 mass spectrometer (Waters Corporation, Milford, MA) operating in full scan, high-resolution positive mode, and ESI mode. The MS source capillary was maintained at 3 kV in positive ESI and the extractor at 5 V. The cone voltage was set at 25 V. The desolvation and source temperatures were established at 500 and 150 °C, respectively. The desolvation and cone gas were maintained at 800 and 20 L/h. The mass spectrometer operated using a scan time of 0.15 ms, and the mass range was 50 to 1200 Da centroid mode. Leucine enkephalin was used as Lock Spray at 2 ng/mL (556.2771 Da), scan time 0.2 s with interval of 10 s and average of 10 scans.

Chiral integrity was proven by HPLC (chiral stationary phase): hardware from UltiMate instrument series (Dionex): HPG-3200SD binary pump, WPS-3000 autosampler, TCC-3200 thermostated column compartment, DAD-3000 detector, SRD-3400 degasser, ValveMate 2 (Gilson) solvent valves. Column, solvent, flow, and retention time (*t*_r_) are indicated in each product description. Data acquisition was done using the Dionex Chromeleon 6.8 software package.

Chiral integrity was also proven by SFC: CO_2_ supply: Agilent G4301A; pump: Agilent G4302A; UV detector: Agilent G1315C. Column, solvent, flow, and retention time (*t*_r_) are indicated in each product description.

Chiral preparative HPLC chromatography were performed using a Gilson 215 autosampler, a Varian SD1 pump equipped, and a Dionex DAD-3000 UV detector. Column, solvent, flow, and retention time (*t*_r_) are indicated in each product description. Data acquisition was done using the Dionex Chromeleon 6.8 software package.

Chiral preparative SFC chromatography was performed using a Sepiatec SFC-660 system. Column, solvent, flow, and retention time (*t*_r_) are indicated in each product description.

^1^H and ^13^C NMR spectra were recorded at rt with a Bruker Avance II 400 (400 MHz for ^1^H, 100 MHz for ^13^C) or a Bruker Avance HD (500 MHz for ^1^H, 125 MHz for ^13^C). Chemical shifts (δ) are reported in parts per million (ppm) relative to the deuterated solvent as internal standard (δ H: CDCl_3_ 7.26 ppm, DMSO-*d*_6_ 2.50 ppm); multiplicities, s = singlet, d = doublet, t = triplet, q = quadruplet, m = multiplet, br = broad signal; coupling constants are given in Hz.

### Preparation of Final Products **1** to **35** (Tables 1 and 2)

(5-Chloropyridin-3-yl)(1,4-diazepan-1-yl)methanone hydrochloride (**2**).

#### General Procedure **D** (Amide Coupling)

To a solution of *tert*-butyl 1,4-diazepane-1-carboxylate (817 mg, 4 mmol, 1 equiv), 5-chloronicotinic acid (715 mg, 4.4 mmol, 1.1 equiv), and DIPEA (2.05 mL, 12.00 mmol, 3 equiv) in DMF (20 mL) was added HATU (1.73 g, 4.4 mmol, 1.1 equiv). The reaction mixture was stirred at rt and monitored by LC–MS. The mixture was directly purified by prep. HPLC under basic conditions and dried by Genevac overnight to give the Boc-protected (5-chloropyridin-3-yl)(1,4-diazepan-1-yl)methanone as an orange thick oil (1.34 g, 98%). LC–MS (basic): *t*_r_ = 0.82 min; [M + H]^+^ = 340.17. ^1^H NMR (400 MHz, DMSO): δ 8.73 (m, 1H), 8.52 (m, 1H), 7.97 (m, 1H), 3.71–3.38 (m, 8H), 1.80–1.53 (m, 2H), 1.42–1.29 (m, 9H).

Boc-protected (5-chloropyridin-3-yl)(1,4-diazepan-1-yl)methanone (1.57 g, 3.9 mmol, 1 equiv) was dissolved in MeOH (30 mL), and 4 M HCl solution in dioxane (5 mL, 20 mmol, 5 equiv) was added. The reaction mixture was stirred at rt for 1 h. The solvent was removed under reduced pressure, and the compound **2** was obtained as a white solid. (1.08 g, 100%). LC–MS (basic): *t*_r_ = 0.48 min; [M + H]^+^ = 240.13. ^1^H NMR (400 MHz, DMSO): δ HCl salt, 9.20–9.16 (br s, 2H), 8.73 (s, 1H), 8.64 (s, 1H), 8.11 (s, 1H), 3.72 (m, 1H), 3.61 (m, 1H), 3.45–3.38 (m, 2H), 3.32–3.16 (m, 4H), 1.91–2.09 (m, 2H).

#### General Procedure **A1** (Reductive Amination)

To a solution of **2** (0.10 mmol, 1 equiv) and DIPEA (0.3 mmol, 3 equiv) in DCM (1 mL) were added the aldehyde (0.12 mmol, 1.2 equiv) and NaBH(OAc)_3_ (0.25 mmol, 2.5 equiv). The reaction mixture was stirred at rt overnight. After an LC–MS control, sat. aq. NaHCO_3_ was added, and the product was extracted with DCM. The org. layer was filtered through a phase separator cartridge, and the solvent was removed under reduced pressure. The residue was purified by prep HPLC under basic conditions.

#### General Procedure **A2** (Reductive Amination)

To a solution of **2** (0.08 mmol, 1 equiv) and DIPEA (0.32 mmol, 4 equiv) in DMF (0.8 mL) were added the aldehyde (0.12 mmol, 1.5 equiv) and NaBH(OAc)_3_ (0.14 mmol, 1.7 equiv). The reaction mixture was stirred at rt overnight. After an LC–MS control, the mixture was diluted with H_2_O (0.2 mL) and was directly purified by prep. HPLC under basic conditions.

#### (4-(2-Chlorobenzyl)-1,4-diazepan-1-yl)(5-chloropyridin-3-yl)methanone (**1**)

Prepared according to general procedure **A1** (25 mg, 70%) from 2-chlorobenzaldehyde. LC–MS (basic): *t*_r_ = 1.02 min; [M + H]^+^ = 364.10. ^1^H NMR (400 MHz, DMSO): δ 8.69 (dd, *J*_1_ = 2.3 Hz, *J*_2_ = 5.6 Hz, 1H), 8.56 (dd, *J*_1_ = 1.7 Hz, *J*_2_ = 7.6 Hz, 1H), 8.00 (dt, *J*_1_ = 2.1 Hz, *J*_2_ = 14.7 Hz, 1H), 7.54–7.24 (m, 4H), 3.72–3.64 (m, 4H), 3.41–3.38 (m, 2H), 2.78 (m, 1H), 2.72–2.65 (m, 2H), 2.62 (m, 1H), 1.84 (m, 1H), 1.72 (m, 1H).

#### (5-Chloropyridin-3-yl)(4-(2-methoxybenzyl)-1,4-diazepan-1-yl)methanone (**3**)

Prepared according to general procedure **A1** (25 mg, 70%) from 2-methoxybenzaldehyde. LC–MS (basic): *t*_r_ = 0.90 min; [M + H]^+^ = 360.13. ^1^H NMR (400 MHz, DMSO): δ 8.69 (t, *J* = 2.6 Hz, 1H), 8.55 (dd, *J*_1_ = 4.0 Hz, *J*_2_ = 1.6 Hz, 1H), 8.00 (dt, *J*_1_ = 11.4 Hz, *J*_2_ = 2.0 Hz, 1H), 7.33 (m, 1H), 7.22 (m, 1H), 6.98–6.87 (m, 2H), 3.75 (d, *J* = 14.8 Hz, 2H), 3.68–3.57 (m, 5H), 3.39–3.36 (m, 2H), 2.73 (m, 1H), 2.62–2.67 (m, 2H), 2.57 (m, 1H), 1.83 (m, 1H), 1.72 (m, 1H).

#### (5-Chloropyridin-3-yl)(4-(4-methylbenzyl)-1,4-diazepan-1-yl)methanone (**4**)

Prepared according to general procedure **A1** (25 mg, 71%) from *p*-tolualdehyde. LC–MS (basic): *t*_r_ = 0.98 min; [M + H]^+^ = 344.19. ^1^H NMR (400 MHz, DMSO): δ 8.69 (t, *J* = 2.7 Hz, 1H), 8.55 (dd, *J*_1_ = 1.7 Hz, *J*_2_ = 3.5 Hz, 1H), 8.00 (dt, *J*_1_ = 2.0 Hz, *J*_2_ = 9.4 Hz, 1H), 7.22 (m, 1H), 7.11–7.17 (m, 2H), 7.09 (m, 1H), 3.65–3.53 (m, 4H), 3.39–3.33 (m, 2H), 2.69 (m, 1H), 2.62 (m, 1H), 2.53–2.58 (m, 2H), 2.26 (d, *J* = 10.8 Hz, 3H), 1.81 (m, 1H), 1.69 (m, 1H).

#### (5-Chloropyridin-3-yl)(4-(3-methylbenzyl)-1,4-diazepan-1-yl)methanone (**5**)

Prepared according to general procedure **A1** (25 mg, 71%) from *m*-tolualdehyde. LC–MS (basic): *t*_r_ = 0.98 min; [M + H]^+^ = 344.19. ^1^H NMR (400 MHz, DMSO): δ 8.69 (t, *J* = 2.6 Hz, 1H), 8.56 (t, *J* = 2.2 Hz, 1H), 8.01 (dt, *J*_1_ = 2.2 Hz, *J*_2_ = 5.0 Hz, 1H), 7.23–7.01 (m, 4H), 3.65–3.54 (m, 4H), 3.39–3.34 (m, 2H), 2.71 (m, 1H), 2.64 (m, 1H), 2.53–2.60 (m, 2H), 2.27 (d, *J* = 19.8 Hz, 3H), 1.83 (m, 1H), 1.70 (m, 1H).

#### (5-Chloropyridin-3-yl)(4-(2-methylbenzyl)-1,4-diazepan-1-yl)methanone (**6**)

Prepared according to general procedure **A1** (24 mg, 71%) from *o*-tolualdehyde. LC–MS (basic): *t*_r_ = 1.03 min; [M + H]^+^ = 344.19. ^1^H NMR (400 MHz, DMSO): δ 8.69 (dd, *J*_1_ = 2.4 Hz, *J*_2_ = 7.4 Hz, 1H), 8.54 (dd, *J*_1_ = 1.7 Hz, *J*_2_ = 13.1 Hz, 1H), 7.99 (dt, *J*_1_ = 2.1 Hz, *J*_2_ = 21.5 Hz, 1H), 7.26–7.08 (m, 4H), 3.66–3.53 (m, 4H), 3.40–3.35 (m, 2H), 2.73 (m, 1H), 2.66–2.55 (m, 3H), 2.31 (d, *J* = 18.1 Hz, 3H), 1.81 (m, 1H), 1.69 (m, 1H).

#### (4-Benzyl-1,4-diazepan-1-yl)(5-chloropyridin-3-yl)methanone (**7**)

Prepared according to general procedure **A1** (24 mg, 73%) from benzaldehyde. LC–MS (basic): *t*_r_ = 0.90 min; [M + H]^+^ = 330.16. ^1^H NMR (400 MHz, DMSO): δ 8.69 (dd, *J*_1_ = 2.4 Hz, *J*_2_ = 4.1 Hz, 1H), 8.55 (dd, *J*_1_ = 4.4 Hz, *J*_2_ = 1.9 Hz, 1H), 8.01 (m, 1H), 7.34–7.20 (m, 5H), 3.66–3.56 (m, 4H), 3.40–3.33 (m, 2H), 2.72 (m, 1H), 2.65 (m, 1H), 2.61–2.53 (m, 2H), 1.83 (m, 1H), 1.71 (m, 1H).

#### (4-((3-Chloropyridin-2-yl)methyl)-1,4-diazepan-1-yl)(5-chloropyridin-3-yl)methanone (**8**)

Prepared according to general procedure **A2** (25 mg, 86%) from 3-chloro-2-formylpyridine. LC–MS (basic): *t*_r_ = 0.76 min; [M + H]^+^ = 365.19. ^1^H NMR (400 MHz, DMSO): δ 8.68 (dd, *J*_1_ = 2.4 Hz, *J*_2_ = 5.1 Hz, 1H), 8.53 (dd, *J*_1_ = 1.7 Hz, *J*_2_ = 9.6 Hz, 1H), 8.45 (ddd, *J*_1_ = 1.3 Hz, *J*_2_ = 4.7 Hz, *J*_3_ = 25.6 Hz, 1H), 7.98 (dt, *J*_1_ = 2.1 Hz, *J*_2_ = 17.8 Hz, 1H), 7.90 (ddd, *J*_1_ = 1.2 Hz, *J*_2_ = 8.0 Hz, *J*_3_ = 12.0 Hz, 1H), 7.35 (m, 1H), 3.83 (d, *J* = 19.9 Hz, 2H), 3.64–3.62 (m, 2H), 3.38–3.34 (m, 2H), 2.85 (m, 1H), 2.78 (m, 1H), 2.74–2.70 (m, 2H), 1.81 (m, 1H), 1.67 (m, 1H).

#### (5-Chloropyridin-3-yl)(4-((3-chloropyridin-4-yl)methyl)-1,4-diazepan-1-yl)methanone (**9**)

Prepared according to general procedure **A2** (25 mg, 86%) from 3-chloroisonicotinaldehyde. LC–MS (basic): *t*_r_ = 0.77 min; [M + H]^+^ = 365.20. ^1^H NMR (400 MHz, DMSO): δ 8.69 (dd, *J*_1_ = 2.4 Hz, *J*_2_ = 5.3 Hz, 1H), 8.58–8.53 (m, 2H), 8.48 (dd, *J*_1_ = 4.9 Hz, *J*_2_ = 18.2 Hz, 1H), 8.02 (dt, *J*_1_ = 2.1 Hz, *J*_2_ = 9.4 Hz, 1H), 7.54 (dd, *J*_1_ = 4.8 Hz, *J*_2_ = 31.0 Hz, 1H), 3.75 (d, *J* = 19.4 Hz, 2H), 3.70–3.65 (m, 2H), 3.42–3.39 (m, 2H), 2.80 (m, 1H), 2.73–2.70 (m, 2H), 2.64 (m, 1H), 1.86 (m, 1H), 1.75 (m, 1H).

#### (5-Chloropyridin-3-yl)(4-(2-(trifluoromethyl)benzyl)-1,4-diazepan-1-yl)methanone (**10**)

Prepared according to general procedure **A1** (28 mg, 69%) from 2-(trifluoromethyl)benzaldehyde. LC–MS (basic): *t*_r_ = 1.09 min; [M + H]^+^ = 398.13. ^1^H NMR (400 MHz, DMSO): δ 8.69 (dd, *J*_1_ = 2.4 Hz, *J*_2_ = 5.9 Hz, 1H), 8.56 (dd, *J*_1_ = 1.7 Hz, *J*_2_ = 6.5 Hz, 1H), 8.01 (dt, *J*_1_ = 2.1 Hz, *J*_2_ = 14.8 Hz, 1H), 7.58–7.87 (m, 3H), 7.46 (m, 1H), 3.76 (d, *J* = 14.9 Hz, 2H), 3.68–3.65 (m, 2H), 3.42–3.38 (m, 2H), 2.75 (m, 1H), 2.67–2.64 (m, 2H), 2.58 (m, 1H), 1.83 (m, 1H), 1.73 (m, 1H).

#### (5-Chloropyridin-3-yl)(4-((2-(trifluoromethyl)pyridin-3-yl)methyl)-1,4-diazepan-1-yl)methanone (**11**)

Prepared according to general procedure **A1** (24 mg, 79%) from 2-(trifluoromethyl)nicotinaldehyde. LC–MS (basic): *t*_r_ = 0.88 min; [M + H]^+^ = 399.33. ^1^H NMR (400 MHz, DMSO): δ 8.69 (dd, *J*_1_ = 2.4 Hz, *J*_2_ = 6.1 Hz, 1H), 8.60 (m, 1H), 8.56 (dd, *J*_1_ = 1.7 Hz, *J*_2_ = 7.1 Hz, 1H), 8.20 (m, 1H), 8.01 (dt, *J*_1_ = 2.1 Hz, *J*_2_ = 14.2 Hz, 1H), 7.71(ddd, *J*_1_ = 4.6 Hz, *J*_2_ = 8.0 Hz, *J*_3_ = 17.8 Hz, 1H), 3.80 (d, *J* = 16.6 Hz, 2H), 3.68–3.65 (m, 2H), 3.41–3.38 (m, 2H), 2.76 (m, 1H), 2.68–2.64 (m, 2H), 2.59 (m, 1H), 1.83 (m, 1H), 1.72 (m, 1H).

#### 2-((4-(5-Chloronicotinoyl)-1,4-diazepan-1-yl)methyl)benzonitrile (**12**)

Prepared according to general procedure **A1** (25 mg, 70%) from 2-cyanobenzaldehyde. LC–MS (basic): *t*_r_ = 0.87 min; [M + H]^+^ = 355.13. ^1^H NMR (400 MHz, DMSO): δ 8.69 (dd, *J*_1_ = 2.4 Hz, *J*_2_ = 8.1 Hz, 1H), 8.56 (dd, *J*_1_ = 1.7 Hz, *J*_2_ = 10.7 Hz, 1H), 8.01 (dt, *J*_1_ = 2.1 Hz, *J*_2_ = 17.6 Hz, 1H), 7.80 (ddd, *J*_1_ = 0.8 Hz, *J*_2_ = 7.7 Hz, *J*_3_ = 13.2 Hz, 1H), 7.71–7.52 (m, 2H), 7.46 (m, 1H), 3.78 (d, *J* = 18.1 Hz, 2H), 3.67–3.64 (m, 2H), 3.40–3.37 (m, 2H), 2.77 (m, 1H), 2.70–2.65 (m, 2H), 2.63 (m, 1H), 1.83 (m, 1H), 1.72 (m, 1H).

#### (4-(4-Chlorobenzyl)-1,4-diazepan-1-yl)(5-chloropyridin-3-yl)methanone (**13**)

Prepared according to general procedure **A2** (24 mg, 81%) from 4-chlorobenzaldehyde. LC–MS (basic): *t*_r_ = 0.99 min; [M + H]^+^ = 364.19. ^1^H NMR (400 MHz, DMSO): δ 8.68 (dd, *J*_1_ = 2.4 Hz, *J*_2_ = 4.4 Hz, 1H), 8.54 (dd, *J*_1_ = 1.7 Hz, *J*_2_ = 4.7 Hz, 1H), 7.99 (dt, *J*_1_ = 2.0 Hz, *J*_2_ = 10.2 Hz, 1H), 7.40–7.28 (m, 4H), 3.66–3.53 (m, 4H), 3.39–3.34 (m, 2H), 2.71 (m, 1H), 2.63 (m, 1H), 2.59–2.53 (m, 2H), 1.82 (m, 1H), 1.71 (m, 1H).

#### 5-Chloro-2-((4-(5-chloronicotinoyl)-1,4-diazepan-1-yl)methyl)benzonitrile (**14**)

Prepared according to general procedure **A1** (27 mg, 68%) from 5-chloro-2-formylbenzonitrile. LC–MS (basic): *t*_r_ = 0.98 min; [M + H]^+^ = 389.08. ^1^H NMR (400 MHz, DMSO): δ 8.69 (dd, *J*_1_ = 2.4 Hz, *J*_2_ = 7.7 Hz, 1H), 8.56 (dd, *J*_1_ = 1.7 Hz, *J*_2_ = 10.1 Hz, 1H), 8.06–7.93 (m, 2H), 7.73 (ddd, *J*_1_ = 2.2 Hz, *J*_2_ = 8.4 Hz, *J*_3_ = 16.9 Hz, 1H), 7.59 (dd, *J*_1_ = 8.4 Hz, *J*_2_ = 26.4 Hz, 1H), 3.76 (d, *J* = 18.5 Hz, 2H), 3.68–3.61 (m, 2H), 3.40–3.37 (m, 2H), 2.76 (m, 1H), 2.70–2.65 (m, 2H), 2.62 (m, 1H), 1.82 (m, 1H), 1.71 (m, 1H).

#### (5-Chloropyridin-3-yl)(4-(cyclopentylmethyl)-1,4-diazepan-1-yl)methanone (**15**)

Prepared according to general procedure **A2** (20 mg, 79%) from cyclopentanecarboxaldehyde. LC–MS (basic): *t*_r_ = 0.99 min; [M + H]^+^ = 322.02. ^1^H NMR (400 MHz, DMSO): δ 8.69 (d, *J* = 2.4 Hz, 1H), 8.53 (t, *J* = 1.5 Hz, 1H), 7.98 (dt, *J*_1_ = 8.9 Hz, *J*_2_ = 2.1 Hz, 1H), 3.63–3.53 (m, 2H), 3.36–3.33 (m, 2H), 2.73 (m, 1H), 2.66–2.57 (m, 3H), 2.34 (d, *J* = 7.4 Hz, 1H), 2.29 (d, *J* = 7.5 Hz, 1H), 1.98 (m, 1H), 1.81–1.77 (m, 1H), 1.70–1.42 (m, 7H), 1.23–1.09 (m, 2H).

#### (5-Chloropyridin-3-yl)(4-(3,3-dimethylbutyl)-1,4-diazepan-1-yl)methanone (**16**)

Prepared according to general procedure **A2** (21 mg, 80%) from 3,3-dimethylbutyraldehyde. LC–MS (basic): *t*_r_ = 0.95 min; [M + H]^+^ = 324.28. ^1^H NMR (400 MHz, DMSO): δ 8.69 (d, *J* = 2.4 Hz, 1H), 8.54 (d, *J* = 1.6 Hz, 1H), 7.99 (dt, *J*_1_ = 2.1 Hz, *J*_2_ = 7.1 Hz, 1H), 3.63–3.52 (m, 2H), 3.37–3.33 (m, 2H), 2.70 (m, 1H), 2.63–2.59 (m, 2H), 2.55 (m, 1H), 2.46–2.36 (m, 2H), 1.79 (m, 1H), 1.69 (m, 1H), 1.34 (m, 1H), 1.27 (m, 1H), 0.85 (d, *J* = 17.7 Hz, 9H).

#### (5-Chloropyridin-3-yl)(4-((tetrahydro-2*H*-pyran-4-yl)methyl)-1,4-diazepan-1-yl)methanone (**17**)

Prepared according to general procedure **A2** (19 mg, 71%) from tetrahydro-pyran-4-carbaldehyde. LC–MS (basic): *t*_r_ = 0.69 min; [M + H]^+^ = 337.94. ^1^H NMR (400 MHz, DMSO): δ 8.69 (d, *J* = 2.4 Hz, 1H), 8.53 (t, *J* = 1.9 Hz, 1H), 7.98 (dt, *J*_1_ = 2.1 Hz, *J*_2_ = 9.3 Hz, 1H), 3.82 (m, 1H), 3.66–3.50 (m, 4H), 3.37–3.20 (m, 3H), 2.72 (m, 1H), 2.65–2.55 (m, 3H), 2.30 (m, 1H), 2.25 (m, 1H), 1.80 (m, 1H), 1.72–1.52 (m, 4H), 1.15–1.02 (m, 2H).

#### (5-Chloropyridin-3-yl)(4-(2,4-dichlorobenzyl)-1,4-diazepan-1-yl)methanone (**18**)

Prepared according to general procedure **A2** (26 mg, 83%) from 2,4-dichlorobenzaldehyde. LC–MS (basic): *t*_r_ = 1.13 min; [M + H]^+^ = 398.15. ^1^H NMR (400 MHz, DMSO): δ 8.69 (dd, *J*_1_ = 2.4 Hz, *J*_2_ = 5.5 Hz, 1H), 8.55 (dd, *J*_1_ = 1.7 Hz, *J*_2_ = 7.5 Hz, 1H), 7.99 (dt, *J*_1_ = 2.1 Hz, *J*_2_ = 17.8 Hz, 1H), 7.58–7.47 (m, 2H), 7.40 (ddd, *J*_1_ = 2.1 Hz, *J*_2_ = 8.4 Hz, *J*_3_ = 18.9 Hz, 1H), 3.73–3.58 (m, 4H), 3.43–3.34 (m, 2H), 2.77 (m, 1H), 2.65–2.70 (m, 2H), 2.61 (m, 1H), 1.84 (m, 1H), 1.72 (m, 1H).

#### (4-(4-Bromo-2-chlorobenzyl)-1,4-diazepan-1-yl)(5-chloropyridin-3-yl)methanone (**19**)

Prepared according to general procedure **A2** (31 mg, 89%) from 4-bromo-2-chlorobenzaldehyde. LC–MS (basic): *t*_r_ = 1.15 min; [M + H]^+^ = 442.10. ^1^H NMR (400 MHz, DMSO): δ 8.69 (dd, *J*_1_ = 5.4 Hz, *J*_2_ = 2.3 Hz, 1H), 8.55 (dd, *J*_1_ = 7.1 Hz, *J*_2_ = 1.6 Hz, 1H), 7.99 (dt, *J*_1_ = 18.1 Hz, *J*_2_ = 2.0 Hz, 1H), 7.67 (dd, *J*_1_ = 13.3 Hz, *J*_2_ = 1.8 Hz, 1H), 7.58–7.39 (m, 2H), 3.68–3.63 (m, 4H), 3.40–3.37 (m, 2H), 2.77 (m, 1H), 2.70–2.65 (m, 2H), 2.61 (m, 1H), 1.83 (m, 1H), 1.72 (m, 1H).

#### (4-(2-Chloro-4-cyclopropoxybenzyl)-1,4-diazepan-1-yl)(5-chloropyridin-3-yl)methanone (**20**)

Prepared according to general procedure **A1** from **2** (1.5 g, 4.51 mmol, 1 equiv) and 2-chloro-4-hydroxybenzaldehyde (793 mg, 4.96 mmol, 1.1 equiv) to give the (4-(2-chloro-4-hydroxybenzyl)-1,4-diazepan-1-yl)(5-chloropyridin-3-yl)methanone as a yellow solid (1.3 g, 76%). LC–MS (acidic): *t*_r_ = 0.58 min; [M + H]^+^ = 380.08. ^1^H NMR (400 MHz, DMSO): δ 9.77 (d, *J* = 8.9 Hz, 1H), 8.70 (dd, *J*_1_ = 2.3 Hz, *J*_2_ = 4.9 Hz, 1H), 8.56 (dd, *J*_1_ = 1.5 Hz, *J*_2_ = 8.4 Hz, 1H), 8.01 (dt, *J*_1_ = 2.0 Hz, *J*_2_ = 19.0 Hz, 1H), 7.25 (dd, *J*_1_ = 8.4 Hz, *J*_2_ = 28.5 Hz, 1H), 6.79 (dd, *J*_1_ = 2.3 Hz, *J*_2_ = 12.6 Hz, 1H), 6.71 (ddd, *J*_1_ = 2.3 Hz, *J*_2_ = 8.4 Hz, *J*_3_ = 18.6 Hz, 1H), 3.68–3.62 (m, 2H), 3.57 (d, *J* = 17.3 Hz, 2H), 3.40–3.37 (m, 2H), 2.74 (m, 1H), 2.68–2.60 (m, 2H), 2.58 (m, 1H), 1.83 (m, 1H), 1.71 (m, 1H).

To a solution of the previous solid (42 mg, 0.1 mmol, 1 equiv) in toluene (0.9 mL) and H_2_O (0.3 mL) were added potassium cyclopropyltrifluoroborate (44 mg, 0.3 mmol, 3 equiv), Cu(OAc)_2_ (98%, 2 mg, 0.01 mmol, 0.1 equiv), 1,10-phenanthroline (2 mg, 0.01 mmol, 0.01 equiv), and K_2_CO_3_ (28 mg, 0.2 mmol, 2 equiv). The reaction was stirred under an O_2_ atmosphere at 70 °C overnight. After an LC–MS control, the mixture was cooled to rt, diluted with brine, and extracted with EtOAc. The org. layer was dried over MgSO_4_, and the solvent was removed under reduced pressure. The crude was purified by prep. HPLC under basic conditions to afford the compound **20** as a yellow oil. (3 mg, 7%). LC–MS (basic): *t*_r_ = 1.11 min; [M + H]^+^ = 420.05.

#### (4-(2-Chloro-4-(cyclopentyloxy)benzyl)-1,4-diazepan-1-yl)(5-chloropyridin-3-yl)methanone (**21**)

NaH (60% in mineral oil, 99 mg, 2.6 mmol, 2.6 equiv) was added portionwise, at 0 °C, to a solution of cyclopentanol (0.22 mL, 2.4 mmol, 2.4 equiv) in DMF (5 mL). The suspension was stirred at rt for 30 min, then a solution of 2-chloro-4-fluorobenzoic acid (176 mg, 1 mmol, 1 equiv) in DMF (5 mL) was added. The reaction mixture was stirred at 75 °C for 5 h, then at rt overnight. After an LC–MS control, the mixture was partitioned between H_2_O and EtOAc. Layers were separated, and the organic one was discarded. The pH was adjusted to 4 with aq. 2 M HCl solution, and the product was extracted with EtOAc. The combined org. layers were dried over MgSO_4_, and the solvent was removed under reduced pressure to afford 2-chloro-4-(cyclopentyloxy)benzoic acid as a beige solid. LC–MS (basic): *t*_r_ = 0.43 min; [M + H]^+^ = 240.93.

To a solution of the previous solid (279 mg, 1 mmol, 1 equiv) in THF (1.5 mL) cooled to 0 °C was added dropwise a solution of 1 M BH_3_·THF complex (1.5 mL, 1.5 mmol, 1.5 equiv). The reaction mixture was stirred overnight while warming up to rt. H_2_O was carefully added, then the product was extracted with EtOAc. The combined org. layers were dried over MgSO_4_, and the solvent was removed under reduced pressure. The crude was purified by FC (hept—50% EtOAc gradient) to afford the (2-chloro-4-(cyclopentyloxy)phenyl)methanol as a colorless oil (0.16 g, 70%). ^1^H NMR (400 MHz, DMSO): δ 7.40 (d, *J* = 8.5 Hz, 1H), 6.93–6.88 (m, 2H), 5.22 (t, *J* = 5.6 Hz, 1H), 4.83 (m, 1H), 4.48 (d, *J* = 5.6 Hz, 2H), 1.93–1.87 (m, 2H), 1.72–1.65 (m, 4H), 1.62–1.55 (m, 2H).

A solution of the previous oil (159 mg, 0.58 mmol, 1 equiv) in DCM (2.5 mL) was added dropwise at 0 °C to a suspension of pyridinium chlorochromate (193 mg, 0.88 mmol, 1.5 equiv) in DCM (2.5 mL). The mixture was stirred at rt for 3 h. The solvent was removed under reduced pressure, and the residue was purified by FC (100% DCM) to afford the 2-chloro-4-(cyclopentyloxy)benzaldehyde as a colorless oil (0.19 g, quant.). LC–MS (basic): *t*_r_ = 1.18 min; [M + H]^+^ = 225.00. ^1^H NMR (500 MHz, DMSO): δ 10.19 (d, *J* = 0.7 Hz, 1H), 7.82 (d, *J* = 8.7 Hz, 1H), 7.13 (d, *J* = 2.4 Hz, 1H), 7.06 (ddd, *J*_1_ = 0.6 Hz, *J*_2_ = 2.4 Hz, *J*_3_ = 8.7 Hz, 1H), 5.01 (m, 1H), 2.01–1.93 (m, 2H), 1.75–1.67 (m, 4H), 1.64–1.58 (m, 2H).

2-Chloro-4-(cyclopentyloxy)benzaldehyde was reacted following general procedure **A1** to afford the compound **21** (19 mg, 43%). LC–MS (basic): *t*_r_ = 1.23 min; [M + H]^+^ = 448.16. ^1^H NMR (400 MHz, DMSO): δ 8.67 (dd, *J*_1_ = 2.4 Hz, *J*_2_ = 6.8 Hz, 1H), 8.53 (dd, *J*_1_ = 1.5 Hz, *J*_2_ = 9.7 Hz, 1H), 7.96 (dt, *J*_1_ = 1.9 Hz, *J*_2_ = 20.1 Hz, 1H), 7.34 (dd, *J*_1_ = 8.6 Hz, *J*_2_ = 30.4 Hz, 1H), 6.91 (dd, *J*_1_ = 2.4 Hz, *J*_2_ = 14.4 Hz, 1H), 6.84 (ddd, *J*_1_ = 2.5 Hz, *J*_2_ = 8.6 Hz, *J*_3_ = 21.0 Hz, 1H), 4.79 (m, 1H), 3.72–3.50 (m, 4H), 3.38–3.36 (m, 2H), 2.75 (m, 1H), 2.68–2.59 (m, 3H), 1.93–1.79 (m, 3H), 1.73–1.52 (m, 7H).

#### (4-(2-Chloro-4-(cyclohexylmethoxy)benzyl)-1,4-diazepan-1-yl)(5-chloropyridin-3-yl)methanone (**22**)

(Bromomethyl)cyclohexane (0.03 mL, 0.2 mmol, 2 equiv) was added to a suspension of (4-(2-chloro-4-hydroxybenzyl)-1,4-diazepan-1-yl)(5-chloropyridin-3-yl)methanone (precursor of compound **20**) (42 mg, 0.1 mmol, 1 equiv) and K_2_CO_3_ (42 mg, 0.3 mmol, 3 equiv) in DMF (1 mL). The mixture was stirred at 100 °C overnight. After an LC–MS control, the mixture was cooled to rt and was purified by prep. HPLC under basic conditions to afford the compound **22** as a white solid. LC–MS (basic): *t*_r_ = 1.43 min; [M + H]^+^ = 476.10. ^1^H NMR (400 MHz, DMSO): δ 8.70 (dd, *J*_1_ = 2.2 Hz, *J*_2_ = 5.8 Hz, 1H), 8.56 (dd, *J*_1_ = 1.6 Hz, *J*_2_ = 7.5 Hz, 1H), 8.00 (m, 1H), 7.35 (dd, *J*_1_ = 8.7 Hz, *J*_2_ = 28.5 Hz, 1H), 6.97 (dd, *J*_1_ = 2.5 Hz, *J*_2_ = 12.7 Hz, 1H), 6.90 (m, 1H), 3.77 (dd, *J*_1_ = 6.4 Hz, *J*_2_ = 9.0 Hz, 2H), 3.69–3.55 (m, 4H), 3.44–3.34 (m, 2H) 2.74 (m, 1H), 2.68–2.62 (m, 2H), 2.59 (m, 1H), 1.62–1.85 (m, 8H), 1.16–1.27 (m, 3H), 0.99–1.06 (m, 2H).

#### (4-(2-Chloro-4-(trifluoromethyl)benzyl)-1,4-diazepan-1-yl)(5-chloropyridin-3-yl)methanone (**23**)

Prepared according to general procedure **A2** (30 mg, 86%) from 2-chloro-4-(trifluoromethyl)benzaldehyde. LC–MS (basic): *t*_r_ = 1.14 min; [M + H]^+^ = 432.16. ^1^H NMR (400 MHz, DMSO): δ 8.69 (dd, *J*_1_ = 2.4 Hz, *J*_2_ = 7.4 Hz, 1H), 8.56 (dd, *J*_1_ = 1.7 Hz, *J*_2_ = 6.8 Hz, 1H), 8.00 (dt, *J*_1_ = 2.1 Hz, *J*_2_ = 14.3 Hz, 1H), 7.86–7.66 (m, 3H), 3.77 (d, *J* = 19.2 Hz, 2H), 3.69–3.65 (m, 2H), 3.42–3.38 (m, 2H), 2.80 (m, 1H), 2.73–2.69 (m, 2H), 2.64 (m, 1H), 1.86 (m, 1H), 1.74 (m, 1H).

#### (4-(4-Amino-2-chlorobenzyl)-1,4-diazepan-1-yl)(5-chloropyridin-3-yl)methanone (**24**)

Prepared according to general procedure **A2** (20 mg, 67%) from 4-amino-2-chlorobenzaldehyde. LC–MS (basic): *t*_r_ = 0.79 min; [M + H]^+^ = 379.20. ^1^H NMR (400 MHz, DMSO): δ 8.68 (dd, *J*_1_ = 2.4 Hz, *J*_2_ = 4.6 Hz, 1H), 8.54 (dd, *J*_1_ = 1.7 Hz, *J*_2_ = 7.6 Hz, 1H), 7.98 (dt, *J*_1_ = 2.0 Hz, *J*_2_ = 19.3 Hz, 1H), 7.06 (dd, *J*_1_ = 8.2 Hz, *J*_2_ = 29.4 Hz, 1H), 6.58 (dd, *J*_1_ = 2.2 Hz, *J*_2_ = 12.9 Hz, 1H), 6.48 (ddd, *J*_1_ = 2.1 Hz, *J*_2_ = 8.2 Hz, *J*_3_ = 18.7 Hz, 1H), 5.32–5.24 (m, 2H), 3.66–3.52 (m, 4H), 3.39–3.32 (m, 2H), 2.71 (m, 1H), 2.65–2.59 (m, 2H), 2.56 (m, 1H), 1.80 (m, 1H), 1.69 (m, 1H).

#### Methyl 3-Chloro-4-((4-(5-chloronicotinoyl)-1,4-diazepan-1-yl)methyl)benzoate (**25**)

Prepared according to general procedure **A2** (57 mg, 84%) from methyl 3-chloro-4-formylbenzoate. LC–MS (basic): *t*_r_ = 1.01 min; [M + H]^+^ = 422.19. ^1^H NMR (400 MHz, DMSO): δ 8.69 (dd, *J*_1_ = 2.3 Hz, *J*_2_ = 6.8 Hz, 1H), 8.56 (dd, *J*_1_ = 1.7 Hz, *J*_2_ = 6.4 Hz, 1H), 8.00 (dt, *J*_1_ = 2.0 Hz, *J*_2_ = 15.8 Hz, 1H), 7.94–7.84 (m, 2H), 7.67 (dd, *J*_1_ = 8.4 Hz, *J*_2_ = 31.5 Hz, 1H), 3.85 (d, *J* = 4.8 Hz, 3H), 3.76 (d, *J* = 18.8 Hz, 2H), 3.69–3.65 (m, 2H), 3.44–3.35 (m, 2H), 2.80 (m, 1H), 2.68–2.73 (m, 2H), 2.64 (m, 1H), 1.85 (m, 1H), 1.73 (m, 1H).

#### 3-Chloro-4-((4-(5-chloronicotinoyl)-1,4-diazepan-1-yl)methyl)benzonitrile (**26**)

Prepared according to general procedure **A2** (25 mg, 80%) from 3-chloro-4-formylbenzonitrile. LC–MS (basic): *t*_r_ = 0.95 min; [M + H]^+^ = 389.18. ^1^H NMR (400 MHz, DMSO): δ 8.69 (dd, *J*_1_ = 2.4 Hz, *J*_2_ = 5.9 Hz, 1H), 8.56 (dd, *J*_1_ = 1.7 Hz, *J*_2_ = 7.6 Hz, 1H), 8.04–7.95 (m, 2H), 7.80 (ddd, *J*_1_ = 1.6 Hz, *J*_2_ = 8.0 Hz, *J*_3_ = 17.8 Hz, 1H), 7.71 (m, 1H), 3.77 (d, *J* = 19.3 Hz, 2H), 3.69–3.65 (m, 2H), 3.41–3.38 (m, 2H), 2.79 (m, 1H), 2.70 (q, *J* = 5.0 Hz, 2H), 2.63 (m, 1H), 1.85 (m, 1H), 1.73 (m, 1H).

#### (4-(4-Bromo-2-methylbenzyl)-1,4-diazepan-1-yl)(5-chloropyridin-3-yl)methanone (**27**)

Prepared according to general procedure **A2** (27 mg, 78%) from 4-bromo-2-methylbenzaldehyde. LC–MS (basic): *t*_r_ = 1.14 min; [M + H]^+^ = 422.15. ^1^H NMR (400 MHz, DMSO): δ 8.69 (dd, *J*_1_ = 2.3 Hz, *J*_2_ = 7.2 Hz, 1H), 8.54 (dd, *J*_1_ = 1.7 Hz, *J*_2_ = 11.8 Hz, 1H), 7.97 (dt, *J*_1_ = 2.0 Hz, *J*_2_ = 27.7 Hz, 1H), 7.40–7.24 (m, 2H), 7.18 (dd, *J*_1_ = 8.1 Hz, *J*_2_ = 30.7 Hz, 1H), 3.60–3.67 (m, 4H), 3.31–3.41 (m, 2H), 2.71 (m, 1H), 2.63 (m, 1H), 2.59–2.53 (m, 2H), 2.30 (d, *J* = 17.3 Hz, 3H), 1.81 (m, 1H), 1.69 (m, 1H).

#### (4-(4-Bromo-2-ethylbenzyl)-1,4-diazepan-1-yl)(5-chloropyridin-3-yl)methanone (**28**)

Prepared according to general procedure **A1** (20 mg, 62%) from 4-bromo-2-ethylbenzaldehyde. LC–MS (basic): *t*_r_ = 1.22 min; [M + H]^+^ = 436.33. ^1^H NMR (400 MHz, DMSO): δ 8.69 (dd, *J*_1_ = 2.3 Hz, *J*_2_ = 6.1 Hz, 1H), 8.55 (dd, *J*_1_ = 1.5 Hz, *J*_2_ = 9.7 Hz, 1H), 7.98 (dt, *J*_1_ = 2.0 Hz, *J*_2_ = 22.3 Hz, 1H), 7.42–7.15 (m, 3H), 3.65–3.54 (m, 4H), 3.42–3.32 (m, 2H), 2.77–2.54 (m, 6H), 1.81 (m, 1H), 1.71 (m, 1H), 1.14 (dt, *J*_1_ = 7.5 Hz, *J*_2_ = 16.4 Hz, 3H).

#### (4-(4-Bromo-2-isopropylbenzyl)-1,4-diazepan-1-yl)(5-chloropyridin-3-yl)methanone (**29**)

To a solution of 4-bromo-2-isopropylbenzoic acid (243 mg, 1 mmol, 1 equiv) in THF (1.5 mL) cooled to 0 °C was added dropwise a solution of 1 M BH_3_·THF complex in THF (2.3 mL, 2.3 mmol, 2.3 equiv). The reaction mixture was stirred for 4 h 30 at rt. H_2_O was carefully added, then the product was extracted with EtOAc. The combined org. layers were dried over MgSO_4_, and the solvent was removed under reduced pressure. The crude was purified by FC (hept—50% EtOAc gradient) to afford the (4-bromo-2-isopropylphenyl)methanol as a white solid (0.25 g, quant.). ^1^H NMR (400 MHz, DMSO): δ 7.41 (d, *J* = 1.7 Hz, 1H), 7.37–7.29 (m, 2H), 5.17 (t, *J* = 5.2 Hz, 1H), 4.52 (d, *J* = 5.1 Hz, 2H), 3.14 (m, 1H), 1.17 (d, *J* = 6.8 Hz, 6H).

A solution of the previous solid (238 mg, 1 mmol, 1 equiv) in DCM (5 mL) was added dropwise at 0 °C to a suspension of pyridinium chlorochromate (330 mg, 1.5 mmol, 1.5 equiv) in DCM (5 mL). The mixture was stirred at rt for 5 h. The solvent was removed under reduced pressure, and the residue was purified by FC (hept—20% EtOAc gradient) to afford the 4-bromo-2-isopropylbenzaldehyde as a colorless oil (0.15 g, 65%).

4-Bromo-2-isopropylbenzaldehyde was reacted following general procedure **A1** to afford the compound **29** (14 mg, 32%) from. LC–MS (basic): *t*_r_ = 1.23 min; [M + H]^+^ = 449.87. ^1^H NMR (400 MHz, DMSO): δ 8.69 (dd, *J*_1_ = 2.3 Hz, *J*_2_ = 5.4 Hz, 1H), 8.54 (dd, *J*_1_ = 1.6 Hz, *J*_2_ = 8.9 Hz, 1H), 7.97 (dt, *J*_1_ = 2.0 Hz, *J*_2_ = 23.0 Hz, 1H), 7.42 (dd, *J*_1_ = 2.0 Hz, *J*_2_ = 12.5 Hz, 1H), 7.29 (m, 1H), 7.18 (m, 1H), 3.68–3.49 (m, 4H), 3.22–3.41 (m, 3H), 2.70 (m, 1H), 2.62–2.57 (m, 2H), 2.52–2.55 (m, 1H), 1.79 (m, 1H), 1.68 (m, 1H), 1.16 (dd, *J*_1_ = 6.8 Hz, *J*_2_ = 18.4 Hz, 6H).

#### (4-(4-Bromo-2-isobutylbenzyl)-1,4-diazepan-1-yl)(5-chloropyridin-3-yl)methanone (**30**)

A 2 M isobutylmagnesium bromide solution in Et_2_O (2.5 mL, 5.0 mmol, 5 equiv) was added dropwise and at 0 °C to a solution of 4-bromo-2-fluorobenzoic acid (223 mg, 1 mmol, 1 equiv) in THF (2 mL). The reaction mixture was stirred at rt overnight. The mixture was cooled to 0 °C, and aq. 2 M HCl solution (3.5 mL, 7 mmol, 7 equiv) was added dropwise. The product was extracted with EtOAc, and the combined org. layers were dried over MgSO_4_. The solvent was removed under reduced pressure to afford the 4-bromo-2-isobutylbenzoic acid as a light yellow solid (0.36 g, quant.). LC–MS (basic): *t*_r_ = 0.48 min; [M – H]^−^ = 255.15. ^1^H NMR (400 MHz, DMSO): δ 13.05 (m, 1H), 7.70 (d, *J* = 8.8 Hz, 1H), 7.52–7.49 (m, 2H), 2.82 (d, *J* = 7.1 Hz, 2H), 1.80 (m, 1H), 0.84 (d, *J* = 6.5 Hz, 6H).

To a solution of the previous solid (294 mg, 1 mmol, 1 equiv) in THF (1.5 mL) cooled to 0 °C was added dropwise a solution of 1 M BH_3_·THF complex in THF (2 mL, 2 mmol, 2 equiv). The reaction mixture was stirred at rt for 2 h. H_2_O was carefully added, then the product was extracted with EtOAc. The combined org. layers were dried over MgSO_4_, and the solvent was removed under reduced pressure. The crude was purified by FC (hept—20% EtOAc gradient) to afford the (4-bromo-2-isobutylphenyl)methanol as a colorless oil (0.09 g, 39%). ^1^H NMR (400 MHz, DMSO): δ 7.40–7.32 (m, 2H), 7.29 (s, 1H), 5.18 (t, *J* = 5.6 Hz, 1H), 4.48 (d, *J* = 5.3 Hz, 2H), 2.45 (d, *J* = 7.1 Hz, 2H), 1.80 (m, 1H), 0.87 (d, *J* = 6.5 Hz, 6H).

A solution of the previous oil (94 mg, 0.33 mmol, 1 equiv) in DCM (2 mL) was added dropwise at 0 °C to a suspension of pyridinium chlorochromate (110 mg, 0.5 mmol, 1.5 equiv) in DCM (2 mL). The mixture was stirred at rt for 5 h. The solvent was removed under reduced pressure, and the residue was purified by FC (100% DCM) to afford the 4-bromo-2-isobutylbenzaldehyde as a colorless oil (0.08 g, quant.). ^1^H NMR (400 MHz, DMSO): δ 10.23 (s, 1H), 7.76 (m, 1H), 7.66 (dd, *J*_1_ = 1.9 Hz, *J*_2_ = 8.3 Hz, 1H), 7.59 (d, *J* = 1.8 Hz, 1H), 2.90 (d, *J* = 7.2 Hz, 2H), 1.78 (m, 1H), 0.88 (d, *J* = 6.6 Hz, 6H).

4-Bromo-2-isobutylbenzaldehyde was reacted following general procedure **A1** to afford the compound **30** (13 mg, 28%) from. LC–MS (basic): *t*_r_ = 1.30 min; [M + H]^+^ = 464.18. ^1^H NMR (400 MHz, DMSO): δ 8.69 (dd, *J*_1_ = 5.9 Hz, *J*_2_ = 2.4 Hz, 1H), 8.55 (dd, *J*_1_ = 6.8 Hz, *J*_2_ = 1.5 Hz, 1H), 7.98 (dt, *J*_1_ = 20.8 Hz, *J*_2_ = 2.0 Hz, 1H), 7.36–7.21 (m, 3H), 3.71–3.53 (m, 4H), 3.43–3.30 (m, 2H), 2.71 (m, 1H), 2.58–2.62 (m, 2H), 2.48–2.56 (overlapped m, 3H), 1.88–1.77 (m, 2H), 1.69 (m, 1H), 0.86 (dd, *J*_1_ = 16.2 Hz, *J*_2_ = 6.6 Hz, 6H).

#### (4-(4-Bromo-2-isopropoxybenzyl)-1,4-diazepan-1-yl)(5-chloropyridin-3-yl)methanone (**31**)

Prepared according to general procedure **A1** (29 mg, 63%) from 4-bromo-2-isopropoxybenzaldehyde. LC–MS (basic): *t*_r_ = 1.15 min; [M + H]^+^ = 465.94. ^1^H NMR (400 MHz, DMSO): δ 8.69 (t, *J* = 2.6 Hz, 1H), 8.54 (dd, *J*_1_ = 1.6 Hz, *J*_2_ = 5.8 Hz, 1H), 7.98 (dt, *J*_1_ = 2.0 Hz, *J*_2_ = 16.8 Hz, 1H), 7.27 (dd, *J*_1_ = 8.1 Hz, *J*_2_ = 28.3 Hz, 1H), 7.15 (dd, *J*_1_ = 1.5 Hz, *J*_2_ = 11.9 Hz, 1H), 7.07 (ddd, *J*_1_ = 1.7 Hz, *J*_2_ = 8.1 Hz, *J*_3_ = 19.2 Hz, 1H), 4.64 (m, 1H), 3.69–3.59 (m, 4H), 3.43–3.33 (m, 2H), 2.74 (m, 1H), 2.65–2.62 (m, 2H), 2.58 (m, 1H), 1.83 (m, 1H), 1.71 (m, 1H), 1.24 (dd, *J*_1_ = 6.0 Hz, *J*_2_ = 13.5 Hz, 6H).

#### (4-(4-Bromo-2-hydroxybenzyl)-1,4-diazepan-1-yl)(5-chloropyridin-3-yl)methanone (**32**)

Prepared according to general procedure **A2** (24 mg, 71%) from 4-bromo-2-hydroxybenzaldehyde. LC–MS (basic): *t*_r_ = 0.96 min; [M + H]^+^ = 424.13. ^1^H NMR (400 MHz, DMSO): δ 8.69 (dd, *J*_1_ = 2.4 Hz, *J*_2_ = 5.6 Hz, 1H), 8.56 (dd, *J*_1_ = 1.6 Hz, *J*_2_ = 6.8 Hz, 1H), 8.01 (dt, *J*_1_ = 2.0 Hz, *J*_2_ = 12.5 Hz, 1H), 7.07 (dd, *J*_1_ = 7.8 Hz, *J*_2_ = 21.7 Hz, 1H), 6.96–6.90 (m, 2H), 3.76–3.61 (m, 4H), 3.44–3.33 (m, 2H), 2.80 (m, 1H), 2.72–2.62 (m, 3H), 1.85 (m, 1H), 1.73 (m, 1H).

#### Methyl 5-Bromo-2-((4-(5-chloronicotinoyl)-1,4-diazepan-1-yl)methyl)benzoate (**33**)

Prepared according to general procedure **A1** (11 mg, 30%) from methyl 5-bromo-2-formylbenzoate. LC–MS (basic): *t*_r_ = 1.07 min; [M + H]^+^ = 466.23. ^1^H NMR (400 MHz, DMSO): δ 8.69 (dd, *J*_1_ = 2.3 Hz, *J*_2_ = 10.6 Hz, 1H), 8.55 (dd, *J*_1_ = 1.4 Hz, *J*_2_ = 12.6 Hz, 1H), 7.99 (dt, *J*_1_ = 1.9 Hz, *J*_2_ = 24.4 Hz, 1H), 7.76–7.65 (m, 2H), 7.39 (m, 1H), 3.86–3.71 (overlapped m, 2H), 3.81 (d, *J* = 11.3 Hz, 3H), 3.64–3.55 (m, 2H), 3.39–3.23 (m, 2H), 2.69 (m, 1H), 2.62–2.58 (m, 3H), 1.76 (m, 1H), 1.61 (m, 1H).

#### (4-(4-Bromo-2,6-dimethylbenzyl)-1,4-diazepan-1-yl)(5-chloropyridin-3-yl)methanone (**34**)

Prepared according to general procedure **A1** (26 mg, 59%) from 4-bromo-2,6-dimethylbenzaldehyde. LC–MS (basic): *t*_r_ = 1.22 min; [M + H]^+^ = 436.09. ^1^H NMR (400 MHz, DMSO): δ 8.67 (dd, *J*_1_ = 2.4 Hz, *J*_2_ = 10.3 Hz, 1H), 8.51 (dd, *J*_1_ = 1.7 Hz, *J*_2_ = 21.7 Hz, 1H), 7.92 (dt, *J*_1_ = 2.0 Hz, *J*_2_ = 49.5 Hz, 1H), 7.21 (s, 1H), 7.18 (s, 1H), 3.64–3.47 (m, 4H), 3.36–3.27 (m, 2H), 2.66 (m, 1H), 2.59 (m, 1H), 2.53–2.48 (overlapped m, 2H), 2.34 (s, 3H), 2.29 (s, 3H), 1.77 (m, 1H), 1.65 (m, 1H).

#### (4-(1-(2-Chlorophenyl)ethyl)-1,4-diazepan-1-yl)(5-chloropyridin-3-yl)methanone (**35**)

A suspension of **2** (40 mg, 0.1 mmol, 1 equiv), 1-(1-bromo-ethyl)-2-chloro-benzene (0.2 mmol, 2 equiv), and K_2_CO_3_ (42 mg, 0.3 mmol, 3 equiv) in DMF (2 mL) was stirred at 70 °C overnight. The mixture was filtered and was directly purified by prep. HPLC under basic conditions to afford the compound **35** as a white solid (11 mg, 30%). LC–MS (basic): *t*_r_ = 1.08 min; [M + H]^+^ = 378.10. ^1^H NMR (400 MHz, DMSO): δ 8.69 (t, *J* = 2.9 Hz, 1H), 8.53 (dd, *J*_1_ = 1.5 Hz, *J*_2_ = 4.6 Hz, 1H), 7.97 (dt, *J*_1_ = 2.1 Hz, *J*_2_ = 11.5 Hz, 1H), 7.52 (m, 1H), 7.43–7.21 (m, 3H), 4.15 (m, 1H), 3.68–3.59 (m, 2H), 3.36–3.21 (m, 2H), 2.84–2.60 (m, 4H), 1.77–1.52 (m, 2H), 1.24 (dd, *J*_1_ = 6.7 Hz, *J*_2_ = 30.6 Hz, 3H).

### Preparation of Final Products **36** and **38** (Figure 5)

#### Methyl 1,4-Dibenzyl-1,4-diazepane-2-carboxylate (**41**)

To a solution of 1,3-diaminopropane (1.92 mL, 22.7 mmol, 1 equiv) in MeOH (150 mL) was added benzaldehyde (4.63 mL, 45.4 mmol, 2 equiv). The mixture was stirred at rt for 2 h, then was cooled to 0 °C. NaBH_4_ (2.63 g, 60 mmol, 3 equiv) was added portionwise, and the reaction mixture was stirred at rt overnight. After an LC–MS control, the mixture was carefully diluted with H_2_O, and volatiles were removed under reduced pressure. The mixture was partitioned between DCM and aq. sat. NaHCO_3_ solution. The org. layer was dried over MgSO_4_, and the solvent was removed under reduced pressure. The crude was purified by prep. HPLC under basic conditions to afford *N*^1^,*N*^3^-dibenzylpropane-1,3-diamine as a light-yellow oil. (5.61 g, 97%). LC–MS (basic): *t*_r_ = 0.99 min; [M + H]^+^ = 255.20. ^1^H NMR (500 MHz, DMSO): δ 7.32–7.26 (m, 8H), 7.23–7.17 (m, 2H), 3.66 (s, 4H), 3.46–3.15 (overlapped m, 2H), 2.56–2.48 (m, 4H), 1.53 (quint, *J* = 6.8 Hz, 2H).

*N*^1^,*N*^3^-Dibenzylpropane-1,3-diamine (5.61 g, 22.0 mmol, 1 equiv) and K_3_PO_4_ (10.5 g, 48.4 mmol, 2.2 equiv) were suspended in MeCN (55 mL). Methyl 2,3-dibromopropionate (3.16 mL, 24.2 mmol, 1.1 equiv) was added, and the mixture was stirred at 55 °C overnight. The resulting mixture was cooled to rt and diluted with H_2_O. The solution was extracted with *t*BME and the combined org. layers were dried over MgSO_4_ and filtered. The solvent was removed under reduced pressure. The crude was purified by FC (hept—30% EtOAc gradient) to afford the compound **41** as a yellow oil. (1.38 g, 18%). LC–MS (basic): *t*_r_ = 1.28 min; [M + H]^+^ = 339.15. ^1^H NMR (400 MHz, CDCl_3_): δ 7.41–7.22 (m, 10H), 3.96–3.82 (m, 2H), 3.77–3.70 (m, 2H), 3.64 (s, 3H), 3.64–3.58 (overlapped m, 1H), 3.30 (m, 1H), 3.11 (m, 1H), 3.00 (m, 1H), 2.82–2.69 (m, 2H), 2.62 (m, 1H), 1.84 (m, 1H), 1.68 (m, 1H).

#### 1-(*tert*-Butyl) 3-Methyl 1,4-Diazepane-1,3-dicarboxylate (**42**)

##### General Procedure **B** (Hydrogenolysis)

To a degassed solution of **41** (1.38 g, 3.47 mmol, 1 equiv) in MeOH (30 mL) was added 10% wt. Pd/C (370 mg). The resulting suspension was placed under H_2_ (1 bar) and was stirred at rt for 1 h 30. The mixture was filtered through Celite, and the filtrate was concentrated under reduced pressure to obtain the compound **42** as a light-yellow oil. The crude was used in the next step without further purification. (0.55 g, 100%). LC–MS (basic): *t*_r_ = 0.32 min; [M + H]^+^ = 159.12.

#### 1-(*tert*-Butyl) 3-Methyl 1,4-Diazepane-2-carboxylate (**43**)

##### General Procedure **C** (Boc Protection)

To a solution of **42** (549 mg, 3.47 mmol, 1 equiv) and NEt_3_ (1.5 mL, 10.4 mmol, 3 equiv) in DCM (50 mL) was added Boc_2_O (765 mg, 3.47 mmol, 1 equiv). The solution was stirred at rt for 1 h 30. The mixture was diluted with H_2_O, and the org. layer was filtered through a phase separator cartridge. The aq. layer was extracted twice with DCM, and the combined org. layers were dried. The solvent was removed under reduced pressure to give the compound **43** as a light-yellow oil which was used in the next step without further purification. (1.1 g, quant.). LC–MS (basic): *t*_r_ = 0.73 min; [M + H]^+^ = 259.15. ^1^H NMR (500 MHz, DMSO): δ 3.80 (m, 1H), 3.64 (overlapped m, 1H), 3.63 (s, 3H), 3.58–3.43 (m, 2H), 3.28 (m, 1H), 3.15 (m, 1H), 2.99 (m, 1H), 1.70–1.53 (m, 2H), 1.40 (m, 9H).

#### Methyl 1,4-Dibenzyl-1,4-diazepane-5-carboxylate (**44**)

*N*,*N*′-Dibenzylethylenediamine (6.01 g, 20 mmol, 1 equiv) and K_3_PO_4_ (9.53 g, 44 mmol, 2.2 equiv) were suspended in MeCN (50 mL). Methyl 2,4-dibromobutyrate (5.90 g, 22 mmol, 1.1 equiv) was added, and the mixture was stirred at 55 °C overnight. The resulting mixture was cooled to rt and diluted with H_2_O. The solution was extracted with *t*BME, and the combined org. layers were dried over MgSO_4_ and filtered. The solvent was removed under reduced pressure. The crude was purified by FC (hept—20% EtOAc gradient) to afford the compound **44** as a light-yellow oil. (4.05 g, 60%). LC–MS (basic): *t*_r_ = 1.22 min; [M + H]^+^ = 339.30. ^1^H NMR (400 MHz, DMSO): δ 7.35–7.19 (m, 10H), 3.76 (s, 2H), 3.62 (s, 3H), 3.58–3.53 (overlapped m, 1H), 3.53 (s, 2H), 3.08 (dd, *J* = 9.0 Hz, *J* = 14.0 Hz, 1H), 2.70 (m, 1H), 2.61–2.45 (overlapped m, 3H), 2.37 (m, 1H), 2.09 (m, 1H), 1.96 (m, 1H).

#### Methyl 4-Benzyl-1,4-diazepane-5-carboxylate (**45**)

To a solution of **44** (4.05 g, 11.8 mmol, 1 equiv) in DCE (20 mL) was added dropwise 1-chloroethyl chloroformate (1.43 mL, 13 mmol, 1.1 equiv). The reaction mixture was stirred at 85 °C for 1 h. After an LC–MS control, the solvent was removed under reduced pressure and the residue was dissolved in MeOH (40 mL). The solution was stirred at 65 °C for 1 further h. The mixture was cooled to rt, and the solvent was removed under reduced pressure to afford the compound **45** as a light orange solid which was used for the next step without purification. (3.38 g, quant.). LC–MS (basic): *t*_r_ = 0.85 min; [M + H]^+^ = 249.21. ^1^H NMR (500 MHz, DMSO): δ 7.35–7.29 (m, 4H), 7.23 (m, 1H), 3.83–3.74 (m, 2H), 3.62 (s, 3H), 3.59 (dd, *J*_1_ = 5.9 Hz, *J*_2_ = 9.2 Hz, 1H), 2.98 (m, 1H), 2.88–2.79 (m, 2H), 2.66 (m, 1H), 2.60–2.55 (m, 2H), 2.07 (m, 1H), 1.86 (m, 1H).

#### 1-(*tert*-Butyl) 5-Methyl 4-Benzyl-1,4-diazepane-1,5-dicarboxylate (**46**)

The crude amine **45** (3.38 g, 11.3 mmol, 1 equiv) was reacted following general procedure **C**. After a purification via FC (hept—20% EtOAc gradient), the compound **46** was obtained as a light-yellow oil. (3.77 g, 92%). LC–MS (basic): *t*_r_ = 1.16 min; [M + H]^+^ = 349.25. ^1^H NMR (500 MHz, DMSO): δ 7.32 (d, *J* = 4.2 Hz, 4H), 7.24 (m, 1H), 3.84 (m, 1H), 3.78 (m, 1H), 3.69 (t, *J* = 5.9 Hz, 1H), 3.66–3.62 (m, 3H), 3.53–3.34 (m, 3H), 3.21 (m, 1H), 2.96 (m, 1H), 2.67 (m, 1H), 2.07–2.04 (m, 2H), 1.45–1.35 (m, 9H).

#### 1-(*tert*-Butyl) 5-Methyl 1,4-Diazepane-1,5-dicarboxylate (**47**)

The benzyl protected amine **46** (3.77 g, 10.8 mmol, 1 equiv) was reacted following general procedure **B**. The compound **47** was obtained as a colorless oil. (2.85 g, 100%). LC–MS (basic): *t*_r_ = 0.71 min; [M + H]^+^ = 259.20. ^1^H NMR (500 MHz, DMSO): δ 3.63 (br s, 3H), 3.47–3.36 (m, 3H), 3.30 (m, 1H), 3.12 (m, 1H), 2.98 (m, 1H), 2.60–2.50 (overlapped m, 1H), 2.07 (m, 1H), 1.65 (m, 1H), 1.40 (s, 9H).

#### 1-(*tert*-Butyl) 3-Methyl 4-(4-bromo-2-ethylbenzyl)-1,4-diazepane-1,3-dicarboxylate (**50**)

1-(*tert*-Butyl) 3-methyl 1,4-diazepane-2-carboxylate **43** (142 mg, 0.55 mmol, 1 equiv) and 4-bromo-2-ethylbenzaldehyde (129 mg, 0.61 mmol, 1.1 equiv) were reacted following general procedure **A1**. The compound **50** was obtained as a light-yellow thick oil. (0.15 g, 59%). LC–MS (basic): *t*_r_ = 1.37 min; [M + H]^+^ = 455.09. ^1^H NMR (500 MHz, DMSO): δ 7.36 (s, 1H), 7.32 (m, 1H), 7.20 (dd, *J*_1_ = 4.1 Hz, *J*_2_ = 8.1 Hz, 1H), 3.81–3.66 (m, 4H), 3.62 (d, *J* = 26.5 Hz, 4H), 3.56–3.38 (m, 2H), 3.12 (m, 1H), 2.74–2.58 (m, 3H), 1.65–1.36 (overlapped m, 2H), 1.40 (d, *J* = 24.1 Hz, 9H), 1.15–1.08 (m, 3H). Some signals are doubled due to conformers.

#### 1-(*tert*-Butyl) 5-Methyl 4-(4-bromo-2-ethylbenzyl)-1,4-diazepane-1,5-dicarboxylate (**51**)

1-(*tert*-Butyl) 5-methyl 1,4-diazepane-1,5-dicarboxylate **47** (2.79 g, 10.8 mmol, 1 equiv) and 4-bromo-2-ethylbenzaldehyde (2.53 g, 11.9 mmol, 1.1 equiv) were reacted following general procedure **A1**. The compound **51** was obtained as a colorless thick oil. (4.00 g, 81%). LC–MS (basic): *t*_r_ = 1.38 min; [M + H]^+^ = 455.19. ^1^H NMR (500 MHz, DMSO): δ 7.37 (s, 1H), 7.33 (dd, *J*_1_ = 2.0 Hz, *J*_2_ = 8.1 Hz, 1H), 7.24 (t, *J* = 7.8 Hz, 1H), 3.85–3.78 (m, 2H), 3.72–3.60 (overlapped m, 1H), 3.64 (d, *J* = 9.9 Hz, 3H), 3.49–3.41 (m, 2H), 3.39–3.26 (overlapped m, 1H), 3.18 (m, 1H), 2.97 (m, 1H), 2.69–2.61 (m, 3H), 2.08 (m, 1H), 1.99 (m, 1H), 1.39 (d, *J* = 14.0 Hz, 9H), 1.13–1.16 (m, 3H).

#### Methyl 1-(4-Bromo-2-ethylbenzyl)-4-(5-chloronicotinoyl)-1,4-diazepane-2-carboxylate (**52**)

A solution of 1-(*tert*-butyl) 3-methyl 4-(4-bromo-2-ethylbenzyl)-1,4-diazepane-1,3-dicarboxylate **50** (147 mg, 0.32 mmol, 1 equiv) in MeOH (3 mL) was treated with 4 M HCl solution in dioxane (0.4 mL, 1.6 mmol, 5 equiv) at rt overnight. The solvent was removed under reduced pressure. The white solid residue and 5-chloropyridine-3-carboxylic acid (59 mg, 0.36 mmol, 1.1 equiv) were reacted following general procedure **D**. The compound **52** was obtained as a colorless thick oil. (0.13 g, 83%). LC–MS (basic): *t*_r_ = 1.19 min; [M + H]^+^ = 494.00. ^1^H NMR (500 MHz, DMSO): δ 8.73 (m, 1H), 8.52 (m, 1H), 7.90 (m, 1H), 7.38 (d, *J* = 1.6 Hz, 1H), 7.34 (dd, *J*_1_ = 1.8 Hz, *J*_2_ = 8.0 Hz, 1H), 7.24 (m, 1H), 4.07 (dd, *J*_1_ = 5.0 Hz, *J*_2_ = 13.8 Hz, 1H), 3.84 (dd, *J*_1_ = 5.1 Hz, *J*_2_ = 8.7 Hz, 1H), 3.78–3.72 (m, 4H), 3.55–3.68 (m, 3H), 3.47–3.40 (m, 2H), 3.24 (m, 1H), 2.87–2.67 (m, 2H), 1.77 (m, 1H), 1.50 (m, 1H), 1.12–1.16 (m, 3H). Some signals are doubled due to conformers.

#### Methyl 4-(4-Bromo-2-ethylbenzyl)-1-(5-chloronicotinoyl)-1,4-diazepane-5-carboxylate (**53**)

A solution of 1-(*tert*-Butyl) 5-methyl 4-(4-bromo-2-ethylbenzyl)-1,4-diazepane-1,5-dicarboxylate **51** (4.00 g, 8.78 mmol, 1 equiv) in MeOH (80 mL) was treated with 4 M HCl solution in dioxane (11 mL, 44 mmol, 5 equiv) at rt for 5 h. The solvent was removed under reduced pressure. The white solid residue and 5-chloropyridine-3-carboxylic acid (1.60 g, 9.66 mmol, 1.1 equiv) were reacted following general procedure **D**. The compound **53** was obtained as a colorless thick oil. (4.2 g, 97%). LC–MS (basic): *t*_r_ = 1.17 min; [M + H]^+^ = 494.17. ^1^H NMR (500 MHz, DMSO): δ 8.70 (dd, *J*_1_ = 17.0 Hz, *J*_2_ = 2.3 Hz, 1H), 8.52 (dd, *J*_1_ = 19.0 Hz, *J*_2_ = 1.5 Hz, 1H), 7.94 (dt, *J*_1_ = 30.0 Hz, *J*_2_ = 1.9 Hz, 1H), 7.39–7.20 (m, 3H), 3.86–3.72 (m, 4H), 3.67–3.58 (m, 3.5H), 3.47 (m, 0.5H), 3.29–3.38 (overlapped m, 1H), 3.23 (m, 0.5H), 3.16 (m, 0.5H), 2.99 (m, 0.5H), 2.77 (m, 0.5H), 2.70–2.58 (m, 3H), 2.21–2.02 (m, 2H), 1.15 (dt, *J*_1_ = 7.5 Hz, *J*_2_ = 19.0 Hz, 3H).

#### 1-(4-Bromo-2-ethylbenzyl)-4-(5-chloronicotinoyl)-*N*-ethyl-1,4-diazepane-2-carboxamide (**36**)

Methyl 1-(4-bromo-2-ethylbenzyl)-4-(5-chloronicotinoyl)-1,4-diazepane-2-carboxylate **52** (133 mg, 0.27 mmol, 1 equiv) was dissolved in THF (1.5 mL) and H_2_O (0.5 mL). LiOH·H_2_O (11 mg, 0.27 mmol, 1 equiv) was added, and the mixture was stirred at rt overnight. The solvent was removed under reduced pressure. The light-yellow solid residue (24 mg, 0.05 mmol, 1 equiv) and ethylamine hydrochloride (5 mg, 0.06 mmol, 1.2 equiv) were reacted following general procedure **D** to afford the compound **36** (15 mg, 57%). LC–MS (basic): *t*_r_ = 1.05 min; [M + H]^+^ = 506.98.

#### 4-(4-Bromo-2-ethylbenzyl)-1-(5-chloronicotinoyl)-*N*-ethyl-1,4-diazepane-5-carboxamide (**38**)

Methyl 4-(4-bromo-2-ethylbenzyl)-1-(5-chloronicotinoyl)-1,4-diazepane-5-carboxylate **53** (81 mg, 0.16 mmol, 1 equiv) was dissolved in THF (1.5 mL) and H_2_O (0.5 mL). LiOH·H_2_O (7 mg, 0.16 mmol, 1 equiv) was added, and the mixture was stirred at rt overnight. The solvent was removed under reduced pressure. The light-yellow solid residue (61 mg, 0.13 mmol, 1 equiv) and ethylamine hydrochloride (13 mg, 0.15 mmol, 1.2 equiv) were reacted following general procedure **D** to afford the compound **38** (41 mg, 64%). LC–MS (basic): *t*_r_ = 1.03 min; [M + H]^+^ = 506.99.

### Preparation of Final Products **37** and **39** (Figure 5)

#### 1-(*tert*-Butyl) 3-Methyl 4-(5-chloronicotinoyl)-1,4-diazepane-1,3-dicarboxylate (**54**)

1-(*tert*-Butyl) 3-methyl 1,4-diazepane-2-carboxylate **43** (517 mg, 2.00 mmol, 1 equiv) and 5-chloropyridine-3-carboxylic acid (332 mg, 2.00 mmol, 1 equiv) were reacted following general procedure **D**. The compound **54** was obtained as a yellow solid. (0.55 g, 69%). LC–MS (basic): *t*_r_ = 0.88 min; [M + H]^+^ = 398.1.

#### 1-(*tert*-Butyl) 5-Methyl 4-(5-chloronicotinoyl)-1,4-diazepane-1,5-dicarboxylate (**55**)

1-(*tert*-Butyl)5-methyl 1,4-diazepane-1,5-dicarboxylate **47** (517 mg, 2 mmol, 1 equiv) and 5-chloropyridine-3-carboxylic acid (332 mg, 2 mmol, 1 equiv) were reacted following general procedure **D**. The compound **55** was obtained as a yellow solid. (552 mg, 69%). LC–MS (basic): *t*_r_ = 0.88 min; [M + H]^+^ = 398.10.

#### Methyl 4-(4-Bromo-2-ethylbenzyl)-1-(5-chloronicotinoyl)-1,4-diazepane-2-carboxylate (**56**)

A solution of 1-(*tert*-butyl) 3-methyl 4-(5-chloronicotinoyl)-1,4-diazepane-1,3-dicarboxylate **54** (552 mg, 1.26 mmol, 1 equiv) in MeOH (10 mL) was treated with 4 M HCl solution in dioxane (1.6 mL, 6.4 mmol, 5.1 equiv) at rt overnight. The solvent was removed under reduced pressure. The beige solid residue and 4-bromo-2-ethylbenzaldehyde (295 mg, 1.39 mmol, 1.1 equiv) were reacted following general procedure **A1**. The compound **56** was obtained as a white solid (462 mg, 74%). LC–MS (basic): *t*_r_ = 1.21 min; [M + H]^+^ = 494.02.

#### Methyl 1-(4-Bromo-2-ethylbenzyl)-4-(5-chloronicotinoyl)-1,4-diazepane-5-carboxylate (**57**)

A solution of 1-(*tert*-butyl) 5-methyl 4-(5-chloronicotinoyl)-1,4-diazepane-1,5-dicarboxylate **55** (330 mg, 0.83 mmol, 1 equiv) in MeOH (7 mL) was treated with 4 M HCl solution in dioxane (1.05 mL, 4.2 mmol, 5 equiv) at rt for 4 h. The solvent was removed under reduced pressure. The yellow solid residue and 4-bromo-2-ethylbenzaldehyde (195 mg, 0.91 mmol, 1.1 equiv) were reacted following general procedure **A1**. The compound **57** was obtained as a light-yellow thick oil (0.32 g, 79%). LC–MS (basic): *t*_r_ = 1.19 min; [M + H]^+^ = 494.08.

#### 4-(4-Bromo-2-ethylbenzyl)-1-(5-chloronicotinoyl)-*N*-ethyl-1,4-diazepane-2-carboxamide (**37**)

Methyl 4-(4-bromo-2-ethylbenzyl)-1-(5-chloronicotinoyl)-1,4-diazepane-2-carboxylate **56** (462 mg, 0.93 mmol, 1 equiv) was dissolved in THF (6 mL) and H_2_O (2 mL). LiOH·H_2_O (40 mg, 0.93 mmol, 1 equiv) was added, and the mixture was stirred at rt overnight. The solvent was removed under reduced pressure. The light-yellow solid residue (74 mg, 0.15 mmol, 1 equiv) and ethylamine hydrochloride (15 mg, 0.18 mmol, 1.2 equiv) were reacted following general procedure **D** to afford the compound **37** as a white solid (65 mg, 86%). LC–MS (basic): *t*_r_ = 1.09 min; [M + H]^+^ = 506.99.

#### 1-(4-Bromo-2-ethylbenzyl)-4-(5-chloronicotinoyl)-*N*-ethyl-1,4-diazepane-5-carboxamide (**39**)

Methyl 1-(4-bromo-2-ethylbenzyl)-4-(5-chloronicotinoyl)-1,4-diazepane-5-carboxylate **57** (324 mg, 0.65 mmol, 1 equiv) was dissolved in THF (4 mL) and H_2_O (2 mL). LiOH·H_2_O (27.7 mg, 0.65 mmol, 1 equiv) was added, and the mixture was stirred at rt for 5 h. The solvent was removed under reduced pressure. Some of the white solid residue (24 mg, 0.05 mmol, 1 equiv) and ethylamine hydrochloride (5 mg, 0.06 mmol, 1.2 equiv) were reacted following general procedure **D** to afford the compound **39** as a white solid (22.3 mg, 88%). LC–MS (basic): *t*_r_ = 1.05 min; [M + H]^+^ = 507.13.

### Preparation of Final Product **40** (Figure 5)

#### Methyl 1,4-Dibenzyl-1,4-diazepane-6-carboxylate (**48**)

*N*,*N*^′^-Dibenzylethylenediamine (1.18 mL, 4.00 mmol, 1 equiv) and K_3_PO_4_ (1.91 g, 8.80 mmol, 2.2 equiv) were suspended in MeCN (10 mL). Methyl 3-bromo-2-(bromomethyl)propionate (0.64 mL, 4.40 mmol, 1.1 equiv) was added, and the mixture was stirred at 55 °C for 8 h. The resulting mixture was cooled to rt and diluted with H_2_O. The solution was extracted with *t*BME, and the combined org. layers were dried over MgSO_4_ and filtered. The solvent was removed under reduced pressure. The crude was purified by FC (hept—30% EtOAc gradient) to afford the compound **48** as a light-yellow oil. (1.46 g, quant.). LC–MS (basic): *t*_r_ = 1.26 min; [M + H]^+^ = 339.13. ^1^H NMR (400 MHz, DMSO): δ 7.34–7.21 (m, 10H), 3.69–3.60 (m, 4H), 3.46 (s, 3H), 2.80–2.97 (m, 5H), 2.54–2.61 (m, 4H).

#### Methyl 1,4-Diazepane-6-carboxylate Dihydrochloride (**49**)

Methyl 1,4-dibenzyl-1,4-diazepane-6-carboxylate 48 (1.35 g, 4.00 mmol, 1 equiv) was reacted following general procedure **B** for 3 h 30. The residue was treated with 1.25 M HCl solution in MeOH. The solvent was removed under reduced pressure to afford the compound **49** as a colorless thick oil. (0.95 g, 100%). LC–MS (basic): *t*_r_ = 0.29 min; [M + H]^+^ = 159.21.

#### Methyl 1-(5-Chloronicotinoyl)-1,4-diazepane-6-carboxylate (**58**)

Methyl 1,4-diazepane-6-carboxylate dihydrochloride **49** (462 mg, 2.00 mmol, 1 equiv) and 5-chloropyridine-3-carboxylic acid (332 mg, 2.00 mmol, 1 equiv) were reacted following general procedure **D**. The compound **58** was obtained as a colorless thick oil. (324 mg, 54%). LC–MS (basic): *t*_r_ = 0.51 min; [M + H]^+^ = 298.12. ^1^H NMR (500 MHz, DMSO): δ 8.71 (d, *J* = 2.2 Hz, 1H), 8.58 (d, *J* = 1.4 Hz, 1H), 8.04 (m, 1H), 4.21 (dd, *J*_1_ = 5.1 Hz, *J*_2_ = 13.5 Hz, 1H), 3.64–3.61 (m, 3H), 3.52–3.47 (m, 3H), 3.06–2.84 (m, 4H), 2.76–2.69 (m, 2H).

#### Methyl 1-(4-Bromo-2-ethylbenzyl)-4-(5-chloronicotinoyl)-1,4-diazepane-6-carboxylate (**59**)

Methyl 1-(5-chloronicotinoyl)-1,4-diazepane-6-carboxylate **58** (324 mg, 1.09 mmol, 1 equiv) and 4-bromo-2-ethylbenzaldehyde (255 mg, 1.20 mmol, 1.1 equiv) were reacted following general procedure **A1**. The compound **59** was obtained as a colorless thick oil (345 mg, 64%). LC–MS (basic): *t*_r_ = 1.16 min; [M + H]^+^ = 493.98.

#### Lithium 1-(4-Bromo-2-ethylbenzyl)-4-(5-chloronicotinoyl)-1,4-diazepane-6-carboxylate (**60**)

Methyl 1-(4-bromo-2-ethylbenzyl)-4-(5-chloronicotinoyl)-1,4-diazepane-6-carboxylate **59** (345 mg, 0.66 mmol, 1 equiv) was dissolved in THF (4.5 mL) and H_2_O (1.5 mL). LiOH·H_2_O (28 mg, 0.66 mmol, 1 equiv) was added, and the mixture was stirred at rt overnight. The solvent was removed under reduced pressure to afford the compound **60** as a light-yellow solid which was used in the next step without further purification. (350 mg, quant.). LC–MS (basic): *t*_r_ = 0.55 min; [M + H]^+^ = 479.99.

#### 1-(4-Bromo-2-ethylbenzyl)-4-(5-chloronicotinoyl)-*N*-ethyl-1,4-diazepane-6-carboxamide (**40**)

Lithium 1-(4-bromo-2-ethylbenzyl)-4-(5-chloronicotinoyl)-1,4-diazepane-6-carboxylate **60** (24 mg, 0.05 mmol, 1 equiv) and ethylamine hydrochloride (5 mg, 0.06 mmol, 1.2 equiv) were reacted following general procedure **D** to afford the compound **40** (11 mg, 44%). LC–MS (basic): *t*_r_ = 1.03 min; [M + H]^+^ = 506.98.

### Preparation of Final Products **61** to **71** (Table 2)

#### Step a: Lithium 4-(4-Bromo-2-ethylbenzyl)-1-(5-chloronicotinoyl)-1,4-diazepane-5-carboxylate

Methyl 4-(4-bromo-2-ethylbenzyl)-1-(5-chloronicotinoyl)-1,4-diazepane-5-carboxylate **53** (4.20 g, 8.49 mmol, 1 equiv) was dissolved in THF (60 mL) and H_2_O (20 mL). LiOH·H_2_O (360 mg, 8.49 mmol, 1 equiv) was added, and the mixture was stirred at rt for 5 h. The solvent was removed under reduced pressure to afford the intermediate lithium 4-(4-bromo-2-ethylbenzyl)-1-(5-chloronicotinoyl)-1,4-diazepane-5-carboxylate as a white solid which was used in the next step without further purification. (4.16 g, quant.). LC–MS (basic): *t*_r_ = 0.55 min; [M + H]^+^ = 480.18.

#### Step b: 4-(4-Bromo-2-ethylbenzyl)-1-(5-chloronicotinoyl)-*N*-cyclopropyl-1,4-diazepane-5-carboxamide (**61**)

Lithium 4-(4-bromo-2-ethylbenzyl)-1-(5-chloronicotinoyl)-1,4-diazepane-5-carboxylate (24 mg, 0.05 mmol, 1 equiv) and cyclopropylamine (3.4 mg, 0.06 mmol, 1.2 equiv) were reacted following general procedure **D** to afford the compound **61** (21.3 mg, 82%). LC–MS (basic): *t*_r_ = 1.03 min; [M + H]^+^ = 518.98.

#### 4-(4-Bromo-2-ethylbenzyl)-1-(5-chloronicotinoyl)-*N*-isobutyl-1,4-diazepane-5-carboxamide (**62**)

Lithium 4-(4-bromo-2-ethylbenzyl)-1-(5-chloronicotinoyl)-1,4-diazepane-5-carboxylate (24 mg, 0.05 mmol, 1 equiv) and isobutylamine (4.4 mg, 0.06 mmol, 1.2 equiv) were reacted following general procedure **D** to afford the compound **62** (21 mg, 78%). LC–MS (basic): *t*_r_ = 1.14 min; [M + H]^+^ = 535.01.

#### 4-(4-Bromo-2-ethylbenzyl)-1-(5-chloronicotinoyl)-*N*-(cyclohexylmethyl)-1,4-diazepane-5-carboxamide (**63**)

Lithium 4-(4-bromo-2-ethylbenzyl)-1-(5-chloronicotinoyl)-1,4-diazepane-5-carboxylate (24 mg, 0.05 mmol, 1 equiv) and cyclohexanemethylamine (6.8 mg, 0.06 mmol, 1.2 equiv) were reacted following general procedure **D** to afford the compound **63** (22.8 mg, 79%). LC–MS (basic): *t*_r_ = 1.26 min; [M + H]^+^ = 575.06.

#### 4-(4-Bromo-2-ethylbenzyl)-1-(5-chloronicotinoyl)-*N*-benzyl-1,4-diazepane-5-carboxamide (**64**)

Lithium 4-(4-bromo-2-ethylbenzyl)-1-(5-chloronicotinoyl)-1,4-diazepane-5-carboxylate (24 mg, 0.05 mmol, 1 equiv) and benzylamine (6.4 mg, 0.06 mmol, 1.2 equiv) were reacted following general procedure **D** to afford the compound **64** (22.6 mg, 79%). LC–MS (basic): *t*_r_ = 1.14 min; [M + H]^+^ = 569.01.

#### 4-(4-Bromo-2-ethylbenzyl)-1-(5-chloronicotinoyl)-*N*-phenyl-1,4-diazepane-5-carboxamide (**65**)

Lithium 4-(4-bromo-2-ethylbenzyl)-1-(5-chloronicotinoyl)-1,4-diazepane-5-carboxylate (24 mg, 0.05 mmol, 1 equiv) and aniline (5.6 mg, 0.06 mmol, 1.2 equiv) were reacted following general procedure **D** to afford the compound **65** (20.8 mg, 75%). LC–MS (basic): *t*_r_ = 1.18 min; [M + H]^+^ = 555.20.

#### 4-(4-Bromo-2-ethylbenzyl)-1-(5-chloronicotinoyl)-*N*-ethyl-*N*-methyl-1,4-diazepane-5-carboxamide (**66**)

Lithium 4-(4-bromo-2-ethylbenzyl)-1-(5-chloronicotinoyl)-1,4-diazepane-5-carboxylate (24 mg, 0.05 mmol, 1 equiv) and *N*-ethylmethylamine (3.6 mg, 0.06 mmol, 1.2 equiv) were reacted following general procedure **D** to afford the compound **66** (17.5 mg, 67%). LC–MS (basic): *t*_r_ = 1.10 min; [M + H]^+^ = 521.19.

#### 4-(4-Bromo-2-ethylbenzyl)-1-(5-chloronicotinoyl)-*N*,*N*-dimethyl-1,4-diazepane-5-carboxamide (**67**)

Lithium 4-(4-bromo-2-ethylbenzyl)-1-(5-chloronicotinoyl)-1,4-diazepane-5-carboxylate (24 mg, 0.05 mmol, 1 equiv) and dimethylamine hydrochloride (5.0 mg, 0.06 mmol, 1.2 equiv) were reacted following general procedure **D** to afford the compound **67** (17.8 mg, 70%). LC–MS (basic): *t*_r_ = 1.05 min; [M + H]^+^ = 507.17.

#### (4-(4-Bromo-2-ethylbenzyl)-1-(5-chloronicotinoyl)-1,4-diazepan-5-yl)(pyrrolidin-1-yl)methanone (**68**)

Lithium 4-(4-bromo-2-ethylbenzyl)-1-(5-chloronicotinoyl)-1,4-diazepane-5-carboxylate (24 mg, 0.05 mmol, 1 equiv) and pyrrolidine (4.3 mg, 0.06 mmol, 1.2 equiv) were reacted following general procedure **D** to afford the compound **68** (20.3 mg, 76%). LC–MS (basic): *t*_r_ = 1.09 min; [M + H]^+^ = 532.99.

#### (4-(4-Bromo-2-ethylbenzyl)-1-(5-chloronicotinoyl)-1,4-diazepan-5-yl)(piperidin-1-yl)methanone (**69**)

Lithium 4-(4-bromo-2-ethylbenzyl)-1-(5-chloronicotinoyl)-1,4-diazepane-5-carboxylate (24 mg, 0.05 mmol, 1 equiv) and piperidine (5.3 mg, 0.06 mmol, 1.2 equiv) were reacted following general procedure **D** to afford the compound **69** (17.8 mg, 65%). LC–MS (basic): *t*_r_ = 1.19 min; [M + H]^+^ = 547.23.

#### 4-(4-Bromo-2-ethylbenzyl)-1-(5-chloronicotinoyl)-*N*-(cyclohexylmethyl)-*N*-methyl-1,4-diazepane-5-carboxamide (**70**)

Lithium 4-(4-bromo-2-ethylbenzyl)-1-(5-chloronicotinoyl)-1,4-diazepane-5-carboxylate (24 mg, 0.05 mmol, 1 equiv) and (cyclohexylmethyl)(methyl)amine (7.6 mg, 0.06 mmol, 1.2 equiv) were reacted following general procedure **D** to afford the compound **70** (23 mg, 78%). LC–MS (basic): *t*_r_ = 1.34 min; [M + H]^+^ = 589.08.

#### 4-(4-Bromo-2-ethylbenzyl)-1-(5-chloronicotinoyl)-*N*,*N*-diethyl-1,4-diazepane-5-carboxamide (**71**)

Lithium 4-(4-bromo-2-ethylbenzyl)-1-(5-chloronicotinoyl)-1,4-diazepane-5-carboxylate (24 mg, 0.05 mmol, 1 equiv) and diethylamine hydrochloride (6.6 mg, 0.06 mmol, 1.2 equiv) were reacted following general procedure **D** to afford the compound **68** (20.8 mg, 78%). LC–MS (basic): *t*_r_ = 1.16 min; [M + H]^+^ = 535.21.

### Preparation of Final Products **76** to **111** (Figure 8)

#### Lithium 4-(4-Bromo-2-ethylbenzyl)-1-(*tert*-butoxycarbonyl)-1,4-diazepane-5-carboxylic Acid (**73**)

1-(*tert*-Butyl) 5-methyl 4-(4-bromo-2-ethylbenzyl)-1,4-diazepane-1,5-dicarboxylate **51** (1.14 g, 2.51 mmol, 1 equiv) was dissolved in THF (15 mL) and H_2_O (5 mL). LiOH·H_2_O (106 mg, 2.51 mmol, 1 equiv) was added, and the mixture was stirred at rt overnight. The solvent was removed under reduced pressure to afford the compound **73** as a light-yellow solid which was used in the next step without further purification. (1.12 g, quant.). LC–MS (basic): *t*_r_ = 0.64 min; [M + H]^+^ = 441.19. ^1^H NMR (400 MHz, DMSO) δ 7.34–7.23 (m, 3H), 3.81 (qd, 2H), 3.50–3.36 (m, 2H), 3.31–3.15 (m, 4H), 2.98 (d, *J* = 5.6 Hz, 1H), 2.71–2.62 (m, 2H), 2.00–1.86 (m, 1H), 1.79–1.59 (m, 1H), 1.37 (d, *J* = 16.8 Hz, 9H), 1.12 (td, *J* = 7.5, 2.9 Hz, 3H).

#### *tert*-Butyl 4-(4-Bromo-2-ethylbenzyl)-5-(pyrrolidine-1-carbonyl)-1,4-diazepane-1-carboxylate (**74**)

Lithium 4-(4-bromo-2-ethylbenzyl)-1-(*tert*-butoxycarbonyl)-1,4-diazepane-5-carboxylic acid **73** (1.12 g, 2.51 mmol, 1 equiv) and pyrrolidine (216 mg, 3.01 mmol, 1.2 equiv) were reacted following general procedure **D** to afford the compound **74** as a yellow thick oil (1.08 g, 87%). LC–MS (basic): *t*_r_ = 1.30 min; [M + H]^+^ = 494.31. ^1^H NMR (500 MHz, DMSO): δ 7.37–7.30 (m, 2H), 7.23–7.17 (m, 1H), 3.75 (dd, *J* = 14.0, 8.7 Hz, 1H), 3.65–3.53 (m, 3H), 3.53–3.35 (m, 4H), 3.31–3.25 (m, 2H), 3.18 (dt, *J* = 12.4, 7.0 Hz, 1H), 2.84 (d, *J* = 14.4 Hz, 1H), 2.76–2.67 (m, 1H), 2.61–2.53 (m, 2H), 2.18 (d, *J* = 35.1 Hz, 1H), 1.93–1.71 (m, 5H), 1.42 (d, *J* = 11.4 Hz, 9H), 1.09 (dt, *J* = 10.0, 7.4 Hz, 3H). Some signals are doubled due to conformers.

#### (4-(4-Bromo-2-ethylbenzyl)-1,4-diazepan-5-yl)(pyrrolidin-1-yl)methanone Hydrochloride (**75**)

*tert*-Butyl 4-(4-bromo-2-ethylbenzyl)-5-(pyrrolidine-1-carbonyl)-1,4-diazepane-1-carboxylate **74** (1.08 g, 2.18 mmol, 1 equiv) was dissolved in MeOH (15 mL), and 4 M HCl solution in dioxane (3 mL, 12 mmol, 5.5 equiv) was added. The reaction mixture was stirred at rt for 3 h. The solvent was removed under reduced pressure, and the compound **75** was obtained as a beige solid. (1.08 g, quant.). LC–MS (basic): *t*_r_ = 1.24 min; [M + H]^+^ = 394.17. ^1^H NMR (500 MHz, DMSO): δ 9.10 (d, *J* = 142.7 Hz, 2H), 7.50–7.29 (m, 3H), 4.18–3.80 (m, 3H), 3.79–3.67 (m, 2H), 3.32–3.02 (m, 8H), 2.70 (dtd, *J* = 10.5, 7.4, 4.6 Hz, 2H), 2.25 (s, 1H), 2.11 (s, 1H), 1.74 (dddt, *J* = 44.9, 25.8, 12.4, 6.1 Hz, 4H), 1.14 (t, *J* = 7.5 Hz, 3H). Some signals are doubled due to conformers.

#### (4-(4-Bromo-2-ethylbenzyl)-1-(5-cyclopropylnicotinoyl)-1,4-diazepan-5-yl)(pyrrolidin-1-yl)methanone (**76**)

(4-(4-Bromo-2-ethylbenzyl)-1,4-diazepan-5-yl)(pyrrolidin-1-yl)methanone hydrochloride **75** (21.5 mg, 0.05 mmol, 1 equiv) and 5-cyclopropylnicotinic acid (9.8 mg, 0.06 mmol, 1.2 equiv) were reacted following general procedure **D** to afford the compound **76** (23.9 mg, 88%). LC-HRMS: *t*_r_ = 1.005 min; [M + H]^+^ = 539.2018.

#### (4-(4-Bromo-2-ethylbenzyl)-1-(5-(dimethylamino)nicotinoyl)-1,4-diazepan-5-yl)(pyrrolidin-1-yl)methanone (**77**)

(4-(4-Bromo-2-ethylbenzyl)-1,4-diazepan-5-yl)(pyrrolidin-1-yl)methanone hydrochloride **75** (21.5 mg, 0.05 mmol, 1 equiv) and 5-(dimethylamino)pyridine-3-carboxylic acid (10.5 mg, 0.06 mmol, 1.2 equiv) were reacted following general procedure **D** to afford the compound **77** (24.7 mg, 91%). LC-HRMS: *t*_r_ = 0.811 min; [M + H]^+^ = 542.2136.

#### (4-(4-Bromo-2-ethylbenzyl)-1-(5-ethynylnicotinoyl)-1,4-diazepan-5-yl)(pyrrolidin-1-yl)methanone (**78**)

(4-(4-Bromo-2-ethylbenzyl)-1,4-diazepan-5-yl)(pyrrolidin-1-yl)methanone hydrochloride **75** (21.5 mg, 0.05 mmol, 1 equiv) and 5-ethynylnicotinic acid (8.8 mg, 0.06 mmol, 1.2 equiv) were reacted following general procedure **D** to afford the compound **78** (15.7 mg, 60%). LC-HRMS: *t*_r_ = 1.007 min; [M + H]^+^ = 523.1709.

#### (4-(4-Bromo-2-ethylbenzyl)-1-(5-hydroxynicotinoyl)-1,4-diazepan-5-yl)(pyrrolidin-1-yl)methanone (**79**)

(4-(4-Bromo-2-ethylbenzyl)-1,4-diazepan-5-yl)(pyrrolidin-1-yl)methanone hydrochloride **75** (21.5 mg, 0.05 mmol, 1 equiv) and 5-hydroxynicotinic acid (8.3 mg, 0.06 mmol, 1.2 equiv) were reacted following general procedure **D** to afford the compound **79** (20.1 mg, 78%). LC-HRMS: *t*_r_ = 0.835 min; [M + H]^+^ = 515.1661.

#### (4-(4-Bromo-2-ethylbenzyl)-1-(5-cyclobutoxynicotinoyl)-1,4-diazepan-5-yl)(pyrrolidin-1-yl)methanone (**80**)

(4-(4-Bromo-2-ethylbenzyl)-1,4-diazepan-5-yl)(pyrrolidin-1-yl)methanone hydrochloride **75** (21.5 mg, 0.05 mmol, 1 equiv) and 5-cyclobutoxynicotinic acid (11.6 mg, 0.06 mmol, 1.2 equiv) were reacted following general procedure **D** to afford the compound **80** (20.1 mg, 71%). LC-HRMS: *t*_r_ = 1.118 min; [M + H]^+^ = 569.2136.

#### *tert*-Butyl (5-(4-(4-Bromo-2-ethylbenzyl)-5-(pyrrolidine-1-carbonyl)-1,4-diazepane-1-carbonyl)pyridin-3-yl)carbamate (**81**)

(4-(4-Bromo-2-ethylbenzyl)-1,4-diazepan-5-yl)(pyrrolidin-1-yl)methanone hydrochloride **75** (21.5 mg, 0.05 mmol, 1 equiv) and 5-((*tert*-butoxycarbonyl)amino)nicotinic acid (14.3 mg, 0.06 mmol, 1.2 equiv) were reacted following general procedure **D** to afford the compound **81** (16.3 mg, 63%). LC-HRMS: *t*_r_ = 1.091 min; [M + H]^+^ = 614.2346.

#### 1-(5-(4-(4-Bromo-2-ethylbenzyl)-5-(pyrrolidine-1-carbonyl)-1,4-diazepane-1-carbonyl)pyridin-3-yl)pyrrolidin-2-one (**82**)

(4-(4-Bromo-2-ethylbenzyl)-1,4-diazepan-5-yl)(pyrrolidin-1-yl)methanone hydrochloride **75** (21.5 mg, 0.05 mmol, 1 equiv) and 5-(2-oxopyrrolidin-1-yl)nicotinic acid (12.4 mg, 0.06 mmol, 1.2 equiv) were reacted following general procedure **D** to afford the compound **82** (4.7 mg, 16%). LC-HRMS: *t*_r_ = 0.901 min; [M + H]^+^ = 582.2086.

#### 5-(4-(4-Bromo-2-ethylbenzyl)-5-(pyrrolidine-1-carbonyl)-1,4-diazepane-1-carbonyl)-*N*,*N*-dimethylnicotinamide (**83**)

(4-(4-Bromo-2-ethylbenzyl)-1,4-diazepan-5-yl)(pyrrolidin-1-yl)methanone hydrochloride **75** (21.5 mg, 0.05 mmol, 1 equiv) and 5-(dimethylcarbamoyl)nicotinic acid (11.7 mg, 0.06 mmol, 1.2 equiv) were reacted following general procedure **D** to afford the compound **83** (20.1 mg, 70%). LC-HRMS: *t*_r_ = 0.861 min; [M + H]^+^ = 570.2083.

#### 5-(4-(4-Bromo-2-ethylbenzyl)-5-(pyrrolidine-1-carbonyl)-1,4-diazepane-1-carbonyl)pyridine-3-sulfonamide (**84**)

(4-(4-Bromo-2-ethylbenzyl)-1,4-diazepan-5-yl)(pyrrolidin-1-yl)methanone hydrochloride **75** (21.5 mg, 0.05 mmol, 1 equiv) and 5-sulfamoylnicotinic acid (12.1 mg, 0.06 mmol, 1.2 equiv) were reacted following general procedure **D** to afford the compound **84** (14.4 mg, 50%). LC-HRMS: *t*_r_ = 0.889 min; [M + H]^+^ = 578.1436.

#### (4-(4-Bromo-2-ethylbenzyl)-1-(5-phenylnicotinoyl)-1,4-diazepan-5-yl)(pyrrolidin-1-yl)methanone (**85**)

(4-(4-Bromo-2-ethylbenzyl)-1,4-diazepan-5-yl)(pyrrolidin-1-yl)methanone hydrochloride **75** (21.5 mg, 0.05 mmol, 1 equiv) and 5-phenylnicotinic acid (12.0 mg, 0.06 mmol, 1.2 equiv) were reacted following general procedure **D** to afford the compound **85** (20.1 mg, 70%). LC-HRMS: *t*_r_ = 1.131 min; [M + H]^+^ = 575.2020.

#### (4-(4-Bromo-2-ethylbenzyl)-1-(5-(*m*-tolyl)nicotinoyl)-1,4-diazepan-5-yl)(pyrrolidin-1-yl)methanone (**86**)

(4-(4-Bromo-2-ethylbenzyl)-1,4-diazepan-5-yl)(pyrrolidin-1-yl)methanone hydrochloride **75** (21.5 mg, 0.05 mmol, 1 equiv) and 5-(*m*-tolyl)nicotinic acid (12.8 mg, 0.06 mmol, 1.2 equiv) were reacted following general procedure **D** to afford the compound **86** (20 mg, 68%). LC-HRMS: *t*_r_ = 1.202 min; [M + H]^+^ = 589.2183.

#### (4-(4-Bromo-2-ethylbenzyl)-1-(5-(furan-3-yl)nicotinoyl)-1,4-diazepan-5-yl)(pyrrolidin-1-yl)methanone (**87**)

(4-(4-Bromo-2-ethylbenzyl)-1,4-diazepan-5-yl)(pyrrolidin-1-yl)methanone hydrochloride **75** (21.5 mg, 0.05 mmol, 1 equiv) and 5-(furan-3-yl)nicotinic acid (11.4 mg, 0.06 mmol, 1.2 equiv) were reacted following general procedure **D** to afford the compound **87** (20.1 mg, 71%). LC-HRMS: *t*_r_ = 1.046 min; [M + H]^+^ = 565.1818.

#### (4-(4-Bromo-2-ethylbenzyl)-1-(5-(2,3-dihydrobenzofuran-5-yl)nicotinoyl)-1,4-diazepan-5-yl)(pyrrolidin-1-yl)methanone (**88**)

(4-(4-Bromo-2-ethylbenzyl)-1,4-diazepan-5-yl)(pyrrolidin-1-yl)methanone hydrochloride **75** (21.5 mg, 0.05 mmol, 1 equiv) and 5-(2,3-dihydrobenzofuran-5-yl)nicotinic acid (14.5 mg, 0.06 mmol, 1.2 equiv) were reacted following general procedure **D** to afford the compound **88** (19.9 mg, 64%). LC-HRMS: *t*_r_ = 1.117 min; [M + H]^+^ = 617.2138.

#### (4-(4-Bromo-2-ethylbenzyl)-1-(5-(1-methyl-1*H*-pyrazol-4-yl)nicotinoyl)-1,4-diazepan-5-yl)(pyrrolidin-1-yl)methanone (**89**)

(4-(4-Bromo-2-ethylbenzyl)-1,4-diazepan-5-yl)(pyrrolidin-1-yl)methanone hydrochloride **75** (21.5 mg, 0.05 mmol, 1 equiv) and 5-(1-methyl-1*H*-pyrazol-4-yl)nicotinic acid (12.2 mg, 0.06 mmol, 1.2 equiv) were reacted following general procedure **D** to afford the compound **89** (6.7 mg, 23%). LC-HRMS: *t*_r_ = 0.910 min; [M + H]^+^ = 579.2083.

#### (1-(5-(1*H*-Pyrrol-1-yl)nicotinoyl)-4-(4-bromo-2-ethylbenzyl)-1,4-diazepan-5-yl)(pyrrolidin-1-yl)methanone (**90**)

(4-(4-Bromo-2-ethylbenzyl)-1,4-diazepan-5-yl)(pyrrolidin-1-yl)methanone hydrochloride **75** (21.5 mg, 0.05 mmol, 1 equiv) and 5-(1*H*-pyrrol-1-yl)nicotinic acid (11.3 mg, 0.06 mmol, 1.2 equiv) were reacted following general procedure **D** to afford the compound **90** (20.1 mg, 71%). LC-HRMS: *t*_r_ = 1.088 min; [M + H]^+^ = 564.1974.

#### [3,4′-Bipyridin]-5-yl(4-(4-bromo-2-ethylbenzyl)-5-(pyrrolidine-1-carbonyl)-1,4-diazepan-1-yl)methanone (**91**)

(4-(4-Bromo-2-ethylbenzyl)-1,4-diazepan-5-yl)(pyrrolidin-1-yl)methanone hydrochloride **75** (21.5 mg, 0.05 mmol, 1 equiv) and [3,4′-bipyridine]-5-carboxylic acid (12 mg, 0.06 mmol, 1.2 equiv) were reacted following general procedure **D** to afford the compound **91** (20.1 mg, 70%). LC-HRMS: *t*_r_ = 0.818 min; [M + H]^+^ = 576.1983.

#### (4-(4-Bromo-2-ethylbenzyl)-1-(4-(phenylamino)nicotinoyl)-1,4-diazepan-5-yl)(pyrrolidin-1-yl)methanone (**92**)

(4-(4-Bromo-2-ethylbenzyl)-1,4-diazepan-5-yl)(pyrrolidin-1-yl)methanone hydrochloride **75** (21.5 mg, 0.05 mmol, 1 equiv) and 4-(phenylamino)nicotinic acid (12.9 mg, 0.06 mmol, 1.2 equiv) were reacted following general procedure **D** to afford the compound **92** (12.6 mg, 43%). LC-HRMS: *t*_r_ = 0.780 min; [M + H]^+^ = 590.2126.

#### (4-(4-Bromo-2-ethylbenzyl)-1-(4-phenoxynicotinoyl)-1,4-diazepan-5-yl)(pyrrolidin-1-yl)methanone (**93**)

(4-(4-Bromo-2-ethylbenzyl)-1,4-diazepan-5-yl)(pyrrolidin-1-yl)methanone hydrochloride **75** (21.5 mg, 0.05 mmol, 1 equiv) and 4-phenoxynicotinic acid (12.9 mg, 0.06 mmol, 1.2 equiv) were reacted following general procedure **D** to afford the compound **93** (16.3 mg, 55%). LC-HRMS: *t*_r_ = 1.075 min; [M + H]^+^ = 591.1969.

#### (4-(4-Bromo-2-ethylbenzyl)-1-(4-((1-methyl-1*H*-pyrazol-4-yl)amino)nicotinoyl)-1,4-diazepan-5-yl)(pyrrolidin-1-yl)methanone (**94**)

(4-(4-Bromo-2-ethylbenzyl)-1,4-diazepan-5-yl)(pyrrolidin-1-yl)methanone hydrochloride **75** (21.5 mg, 0.05 mmol, 1 equiv) and 4-((1-methyl-1*H*-pyrazol-4-yl)amino)nicotinic acid (13.1 mg, 0.06 mmol, 1.2 equiv) were reacted following general procedure **D** to afford the compound **94** (17.1 mg, 58%). LC-HRMS: *t*_r_ = 0.665 min; [M + H]^+^ = 594.2190.

#### (4-(4-Bromo-2-ethylbenzyl)-1-(4-morpholinonicotinoyl)-1,4-diazepan-5-yl)(pyrrolidin-1-yl)methanone (**95**)

(4-(4-Bromo-2-ethylbenzyl)-1,4-diazepan-5-yl)(pyrrolidin-1-yl)methanone hydrochloride **75** (21.5 mg, 0.05 mmol, 1 equiv) and 4-morpholinonicotinic acid (12.5 mg, 0.06 mmol, 1.2 equiv) were reacted following general procedure **D** to afford the compound **95** (20.1 mg, 69%). LC-HRMS: *t*_r_ = 0.689 min; [M + H]^+^ = 584.2230.

#### (4-(4-Bromo-2-ethylbenzyl)-1-(4-((furan-2-ylmethyl)amino)nicotinoyl)-1,4-diazepan-5-yl)(pyrrolidin-1-yl)methanone (**96**)

(4-(4-Bromo-2-ethylbenzyl)-1,4-diazepan-5-yl)(pyrrolidin-1-yl)methanone hydrochloride **75** (21.5 mg, 0.05 mmol, 1 equiv) and 4-((furan-2-ylmethyl)amino)nicotinic acid (13.1 mg, 0.06 mmol, 1.2 equiv) were reacted following general procedure **D** to afford the compound **96** (20.1 mg, 68%). LC-HRMS: *t*_r_ = 0.739 min; [M + H]^+^ = 594.2082.

#### (4-(4-Bromo-2-ethylbenzyl)-1-(5-methoxy-4-methylnicotinoyl)-1,4-diazepan-5-yl)(pyrrolidin-1-yl)methanone (**97**)

(4-(4-Bromo-2-ethylbenzyl)-1,4-diazepan-5-yl)(pyrrolidin-1-yl)methanone hydrochloride **75** (21.5 mg, 0.05 mmol, 1 equiv) and 5-methoxy-4-methylnicotinic acid (10 mg, 0.06 mmol, 1.2 equiv) were reacted following general procedure **D** to afford the compound **97** (25.3 mg, 93%). LC-HRMS: *t*_r_ = 0.969 min; [M + H]^+^ = 543.1970.

#### (4-(4-Bromo-2-ethylbenzyl)-1-(4,5-dimethylnicotinoyl)-1,4-diazepan-5-yl)(pyrrolidin-1-yl)methanone (**98**)

(4-(4-Bromo-2-ethylbenzyl)-1,4-diazepan-5-yl)(pyrrolidin-1-yl)methanone hydrochloride **75** (21.5 mg, 0.05 mmol, 1 equiv) and 4,5-dimethylnicotinic acid (9.1 mg, 0.06 mmol, 1.2 equiv) were reacted following general procedure **D** to afford the compound **98** (20.1 mg, 76%). LC-HRMS: *t*_r_ = 0.886 min; [M + H]^+^ = 527.2026.

#### (4-(4-Bromo-2-ethylbenzyl)-1-(5-chloro-4-methylnicotinoyl)-1,4-diazepan-5-yl)(pyrrolidin-1-yl)methanone (**99**)

(4-(4-Bromo-2-ethylbenzyl)-1,4-diazepan-5-yl)(pyrrolidin-1-yl)methanone hydrochloride **75** (21.5 mg, 0.05 mmol, 1 equiv) and 5-chloro-4-methylnicotinic acid (10.3 mg, 0.06 mmol, 1.2 equiv) were reacted following general procedure **D** to afford the compound **99** (20.1 mg, 73%). LC-HRMS: *t*_r_ = 1.112 min; [M + H]^+^ = 547.1475.

#### (4-(4-Bromo-2-ethylbenzyl)-1-(6,7-dihydro-5*H*-cyclopenta[*c*]pyridine-4-carbonyl)-1,4-diazepan-5-yl)(pyrrolidin-1-yl)methanone (**100**)

(4-(4-Bromo-2-ethylbenzyl)-1,4-diazepan-5-yl)(pyrrolidin-1-yl)methanone hydrochloride **75** (21.5 mg, 0.05 mmol, 1 equiv) and 6,7-dihydro-5*H*-cyclopenta[*c*]pyridine-4-carboxylic acid (9.8 mg, 0.06 mmol, 1.2 equiv) were reacted following general procedure **D** to afford the compound **100** (20.1 mg, 73%). LC-HRMS: *t*_r_ = 0.881 min; [M + H]^+^ = 539.2021.

#### (4-(4-Bromo-2-ethylbenzyl)-1-(5,6,7,8-tetrahydroisoquinoline-4-carbonyl)-1,4-diazepan-5-yl)(pyrrolidin-1-yl)methanone (**101**)

(4-(4-Bromo-2-ethylbenzyl)-1,4-diazepan-5-yl)(pyrrolidin-1-yl)methanone hydrochloride **75** (21.5 mg, 0.05 mmol, 1 equiv) and 5,6,7,8-tetrahydroisoquinoline-4-carboxylic acid (10.6 mg, 0.06 mmol, 1.2 equiv) were reacted following general procedure **D** to afford the compound **101** (20 mg, 72%). LC-HRMS: *t*_r_ = 0.961 min; [M + H]^+^ = 553.2183.

#### (4-(4-Bromo-2-ethylbenzyl)-1-(6-fluoroisoquinoline-4-carbonyl)-1,4-diazepan-5-yl)(pyrrolidin-1-yl)methanone (**102**)

(4-(4-Bromo-2-ethylbenzyl)-1,4-diazepan-5-yl)(pyrrolidin-1-yl)methanone hydrochloride **75** (21.5 mg, 0.05 mmol, 1 equiv) and 6-fluoroisoquinoline-4-carboxylic acid (11.5 mg, 0.06 mmol, 1.2 equiv) were reacted following general procedure **D** to afford the compound **102** (20.1 mg, 71%). LC-HRMS: *t*_r_ = 1.064 min; [M + H]^+^ = 567.1773.

#### (4-(4-Bromo-2-ethylbenzyl)-1-(8-fluoroisoquinoline-4-carbonyl)-1,4-diazepan-5-yl)(pyrrolidin-1-yl)methanone (**103**)

(4-(4-Bromo-2-ethylbenzyl)-1,4-diazepan-5-yl)(pyrrolidin-1-yl)methanone hydrochloride **75** (21.5 mg, 0.05 mmol, 1 equiv) and 8-fluoroisoquinoline-4-carboxylic acid (11.5 mg, 0.06 mmol, 1.2 equiv) were reacted following general procedure **D** to afford the compound **103** (20.1 mg, 71%). LC-HRMS: *t*_r_ = 1.101 min; [M + H]^+^ = 567.1776.

#### (4-(4-Bromo-2-ethylbenzyl)-1-(5-chloroisoquinoline-4-carbonyl)-1,4-diazepan-5-yl)(pyrrolidin-1-yl)methanone (**104**)

(4-(4-Bromo-2-ethylbenzyl)-1,4-diazepan-5-yl)(pyrrolidin-1-yl)methanone hydrochloride **75** (21.5 mg, 0.05 mmol, 1 equiv) and 5-chloroisoquinoline-4-carboxylic acid (12.5 mg, 0.06 mmol, 1.2 equiv) were reacted following general procedure **D** to afford the compound **104** (20.1 mg, 69%). LC-HRMS: *t*_r_ = 1.081 min; [M + H]^+^ = 583.1479.

#### (4-(4-Bromo-2-ethylbenzyl)-1-(thieno[2,3-*c*]pyridine-4-carbonyl)-1,4-diazepan-5-yl)(pyrrolidin-1-yl)methanone (**105**)

(4-(4-Bromo-2-ethylbenzyl)-1,4-diazepan-5-yl)(pyrrolidin-1-yl)methanone hydrochloride **75** (21.5 mg, 0.05 mmol, 1 equiv) and thieno[2,3-*c*]pyridine-4-carboxylic acid (10.8 mg, 0.06 mmol, 1.2 equiv) were reacted following general procedure **D** to afford the compound **105** (20.1 mg, 72%). LC-HRMS: *t*_r_ = 0.975 min; [M + H]^+^ = 555.1425.

#### (4-(4-Bromo-2-ethylbenzyl)-1-(1*H*-pyrrolo[3,2-*c*]pyridine-7-carbonyl)-1,4-diazepan-5-yl)(pyrrolidin-1-yl)methanone (**106**)

(4-(4-Bromo-2-ethylbenzyl)-1,4-diazepan-5-yl)(pyrrolidin-1-yl)methanone hydrochloride **75** (21.5 mg, 0.05 mmol, 1 equiv) and 1*H*-pyrrolo[3,2-*c*]pyridine-7-carboxylic acid (9.7 mg, 0.06 mmol, 1.2 equiv) were reacted following general procedure **D** to afford the compound **106** (23.6 mg, 88%). LC-HRMS: *t*_r_ = 0.655 min; [M + H]^+^ = 538.1822.

#### (4-(4-Bromo-2-ethylbenzyl)-1-(1,7-naphthyridine-5-carbonyl)-1,4-diazepan-5-yl)(pyrrolidin-1-yl)methanone (**107**)

(4-(4-Bromo-2-ethylbenzyl)-1,4-diazepan-5-yl)(pyrrolidin-1-yl)methanone hydrochloride **75** (21.5 mg, 0.05 mmol, 1 equiv) and 1,7-naphthyridine-5-carboxylic acid (10.4 mg, 0.06 mmol, 1.2 equiv) were reacted following general procedure **D** to afford the compound **107** (19.9 mg, 72%). LC-HRMS: *t*_r_ = 0.944 min; [M + H]^+^ = 550.1817.

#### (4-(4-Bromo-2-ethylbenzyl)-1-(2,7-naphthyridine-4-carbonyl)-1,4-diazepan-5-yl)(pyrrolidin-1-yl)methanone (**108**)

(4-(4-Bromo-2-ethylbenzyl)-1,4-diazepan-5-yl)(pyrrolidin-1-yl)methanone hydrochloride **75** (21.5 mg, 0.05 mmol, 1 equiv) and 2,7-naphthyridine-4-carboxylic acid (10.4 mg, 0.06 mmol, 1.2 equiv) were reacted following general procedure **D** to afford the compound **108** (19.9 mg, 72%). LC-HRMS: *t*_r_ = 0.921 min; [M + H]^+^ = 550.1822.

#### (4-(4-Bromo-2-ethylbenzyl)-1-(1,6-naphthyridine-8-carbonyl)-1,4-diazepan-5-yl)(pyrrolidin-1-yl)methanone (**109**)

(4-(4-Bromo-2-ethylbenzyl)-1,4-diazepan-5-yl)(pyrrolidin-1-yl)methanone hydrochloride **75** (21.5 mg, 0.05 mmol, 1 equiv) and 1,6-naphthyridine-8-carboxylic acid (10.4 mg, 0.06 mmol, 1.2 equiv) were reacted following general procedure **D** to afford the compound **109** (20.2 mg, 73%). LC-HRMS: *t*_r_ = 0.877 min; [M + H]^+^ = 550.1821.

#### (4-(4-Bromo-2-ethylbenzyl)-1-(1*H*-pyrazolo[4,3-*c*]pyridine-7-carbonyl)-1,4-diazepan-5-yl)(pyrrolidin-1-yl)methanone (**110**)

(4-(4-Bromo-2-ethylbenzyl)-1,4-diazepan-5-yl)(pyrrolidin-1-yl)methanone hydrochloride **75** (21.5 mg, 0.05 mmol, 1 equiv) and 1*H*-pyrazolo[4,3-*c*]pyridine-7-carboxylic acid (9.8 mg, 0.06 mmol, 1.2 equiv) were reacted following general procedure **D** to afford the compound **110** (20.1 mg, 75%). LC-HRMS: *t*_r_ = 0.760 min; [M + H]^+^ = 539.1763.

#### 5-(4-(4-Bromo-2-ethylbenzyl)-5-(pyrrolidine-1-carbonyl)-1,4-diazepane-1-carbonyl)-3-methylpyrimidin-4(3*H*)-one (**111**)

(4-(4-Bromo-2-ethylbenzyl)-1,4-diazepan-5-yl)(pyrrolidin-1-yl)methanone hydrochloride **75** (21.5 mg, 0.05 mmol, 1 equiv) and 1-methyl-6-oxo-1,6-dihydropyrimidine-5-carboxylic acid (9.2 mg, 0.06 mmol, 1.2 equiv) were reacted following general procedure **D** to afford the compound **111** (20.1 mg, 76%). LC-HRMS: *t*_r_ = 0.721 min; [M + H]^+^ = 530.1761.

### Preparation of Products **116** to **118** (Scheme 6)

#### *tert*-Butyl 4-(4-Bromo-2-ethylbenzyl)-5-(ethylcarbamoyl)-1,4-diazepane-1-carboxylate (**114**)

Lithium 4-(4-bromo-2-ethylbenzyl)-1-(*tert*-butoxycarbonyl)-1,4-diazepane-5-carboxylic acid **73** (360 mg, 0.81 mmol, 1 equiv) and ethylamine hydrochloride (80.4 mg, 0.97 mmol, 1.2 equiv) were reacted following general procedure **D** to afford the compound **114** as a light-yellow solid (374 mg, 99%). LC–MS (basic): *t*_r_ = 1.19 min; [M + H]^+^ = 468.06.

#### 4-(4-Bromo-2-ethylbenzyl)-*N*-ethyl-1,4-diazepane-5-carboxamide Hydrochloride (**115**)

*tert*-Butyl 4-(4-bromo-2-ethylbenzyl)-5-(ethylcarbamoyl)-1,4-diazepane-1-carboxylate **114** (374 mg, 0.80 mmol, 1 equiv) was dissolved in MeOH (8 mL), and 4 M HCl solution in dioxane (1 mL, 4 mmol, 5 equiv) was added. The reaction mixture was stirred at rt for 4 h. The solvent was removed under reduced pressure, and the compound **115** was obtained as a white solid. (345 mg, quant.). LC–MS (basic): *t*_r_ = 0.97 min; [M + H]^+^ = 368.07.

#### 4-(4-Bromo-2-ethylbenzyl)-*N*-ethyl-1-(thieno[2,3-*c*]pyridine-4-carbonyl)-1,4-diazepane-5-carboxamide (**116**)

4-(4-Bromo-2-ethylbenzyl)-*N*-ethyl-1,4-diazepane-5-carboxamide hydrochloride **115** (81 mg, 0.2 mmol, 1 equiv) and thieno[2,3-*c*]pyridine-4-carboxylic acid (41.5 mg, 0.22 mmol, 1.1 equiv) were reacted following general procedure **D** to afford the compound **116** (90.9 mg, 86%). LC–MS (basic): *t*_r_ = 0.97 min; [M + H]^+^ = 528.88.

#### 4-(4-Bromo-2-ethylbenzyl)-*N*-ethyl-1-(1*H*-pyrrolo[3,2-*c*]pyridine-7-carbonyl)-1,4-diazepane-5-carboxamide (**117**)

4-(4-Bromo-2-ethylbenzyl)-*N*-ethyl-1,4-diazepane-5-carboxamide hydrochloride **115** (81 mg, 0.2 mmol, 1 equiv) and 1*H*-pyrrolo[3,2-*c*]pyridine-7-carboxylic acid (35.7 mg, 0.22 mmol, 1.1 equiv) were reacted following general procedure **D** to afford the compound **117** (77.7 mg, 76%). LC–MS (basic): *t*_r_ = 0.90 min; [M + H]^+^ = 512.06.

#### 4-(4-Bromo-2-ethylbenzyl)-1-(5-cyclopropylnicotinoyl)-*N*-ethyl-1,4-diazepane-5-carboxamide (**118**)

4-(4-Bromo-2-ethylbenzyl)-*N*-ethyl-1,4-diazepane-5-carboxamide hydrochloride **115** (40.5 mg, 0.1 mmol, 1 equiv) and 5-cyclopropylnicotinic acid (17.9 mg, 0.11 mmol, 1.1 equiv) were reacted following general procedure **D** to afford the compound **118** (46 mg, 90%). LC–MS (basic): *t*_r_ = 1.00 min; [M + H]^+^ = 512.98.

### Enantiomerically Pure Compounds **112**, **119**, **121**, and **123** (Scheme 6)

#### (*R*)-(4-(4-Bromo-2-ethylbenzyl)-1-(thieno[2,3-*c*]pyridine-4-carbonyl)-1,4-diazepan-5-yl)(pyrrolidin-1-yl)methanone (**112**)

(4-(4-Bromo-2-ethylbenzyl)-1-(thieno[2,3-*c*]pyridine-4-carbonyl)-1,4-diazepan-5-yl)(pyrrolidin-1-yl)methanone **105** (28.1 mg) was purified by chiral HPLC NP (ChiralPak IA, 5 μm, 30 × 250 mm; isocratic gradient of 50% acetonitrile and 50% ethanol; flow: 43 mL/min; detector wavelength: 219 nm; temperature: 25 °C) to afford the compound **112** (12.7 mg, 45%). Chiral analysis NP (ChiralPak IA, 5 μm, 4.6 × 250 mm; isocratic gradient of 50% acetonitrile and 50% ethanol; flow: 1 mL/min; detector wavelength: 280 nm; temperature: 25 °C): *t*_r_ = 5.027 min.

#### (*R*)-4-(4-Bromo-2-ethylbenzyl)-*N*-ethyl-1-(thieno[2,3-*c*]pyridine-4-carbonyl)-1,4-diazepane-5-carboxamide (**119**)

4-(4-Bromo-2-ethylbenzyl)-*N*-ethyl-1-(thieno[2,3-*c*]pyridine-4-carbonyl)-1,4-diazepane-5-carboxamide **116** (90.9 mg) was purified by chiral HPLC NP (ChiralPak IC, 5 μm, 20 × 250 mm; isocratic gradient of 50% acetonitrile and 50% ethanol; flow: 20 mL/min; detector wavelength: 228 nm; temperature: 25 °C) to afford the compound **119** (44.5 mg, 49%). Chiral analysis NP (ChiralPak IC, 5 μm, 4.6 × 250 mm; isocratic gradient of 50% acetonitrile and 50% ethanol; flow: 1 mL/min; detector wavelength: 210 nm; temperature: 25 °C): *t*_r_ = 6.044 min.

#### (*R*)-4-(4-Bromo-2-ethylbenzyl)-*N*-ethyl-1-(1*H*-pyrrolo[3,2-*c*]pyridine-7-carbonyl)-1,4-diazepane-5-carboxamide (**121**)

4-(4-Bromo-2-ethylbenzyl)-*N*-ethyl-1-(1*H*-pyrrolo[3,2-*c*]pyridine-7-carbonyl)-1,4-diazepane-5-carboxamide **117** (77.7 mg) was purified by chiral HPLC NP (ChiralPak IC, 5 μm, 20 × 250 mm; isocratic gradient of 70% acetonitrile and 30% ethanol; flow: 16 mL/min; detector wavelength: 220 nm; temperature: 25 °C) to afford the compound **121** (34.8 mg, 45%). Chiral analysis NP (ChiralPak IC, 5 μm, 4.6 × 250 mm; isocratic gradient of 70% acetonitrile and 30% ethanol; flow: 0.8 mL/min; detector wavelength: 210 nm; temperature: 25 °C): *t*_r_ = 11.844 min.

#### (*R*)-4-(4-Bromo-2-ethylbenzyl)-1-(5-cyclopropylnicotinoyl)-*N*-ethyl-1,4-diazepane-5-carboxamide (**123**)

4-(4-Bromo-2-ethylbenzyl)-1-(5-cyclopropylnicotinoyl)-*N*-ethyl-1,4-diazepane-5-carboxamide **118** (46 mg) was purified by chiral SFC (ChiralPak IH, 5 μm, 30 × 250 mm; isocratic gradient of 80% CO_2_ and 20% ethanol; flow: 160 mL/min; detector wavelength: 210 nm; BPR: 100 bar; temperature: 40 °C) to afford the compound **123** (21.6 mg, 47%). Chiral analysis SFC (ChiralPak IH, 5 μm, 4.6 × 250 mm; isocratic gradient of 80% CO_2_ and 20% ethanol; flow: 4 mL/min; detector wavelength: 210 nm; BPR: 150 bar; temperature: 25 °C): *t*_r_ = 3.00 min.

### Fluorescence Resonance Energy Transfer-Based Mpro Proteolytic Activity Assay

The enzymatic activity of the recombinant SARS-CoV-2 main protease Mpro was determined by a FRET assay using a custom synthesized peptide substrate with (7-methoxycoumarin-4-yl)acetyl [MCA] as a fluorophore and 2,4-dinitrophenyl (DNP) as a fluorescence quencher: MCA-Ala-Val-Leu-Gln-Ser-Gly-Phe-Arg-Lys(Dnp)-Lsy-NH_2_-trifluoroacetate salt (Bachem AG, Bubendorf CH). This peptide substrate amino acid sequence corresponds to the nsp4/nsp5 (Mpro) cleavage site. A substrate stock solution (10 mM) was prepared in 100% DMSO. 40 μL of a 4 μM substrate solution prepared in H_2_O/Tween-20 0.01% is added to a solution (40 μL) containing Mpro to start the enzymatic reaction. The final concentrations of the assay reaction ingredients (80 μL) are 5 nM [E] Mpro, 2 μM [S] peptide substrate (Km 3.17 μM), 1 mM DTT, 1.2% DMSO, and 0.01% Tween-20 25 mM TRIS pH 7.4. Mpro was diluted (10 nM) from aliquots stored as stock solution (512 μM, −80 °C, storage buffer) in Mpro assay buffer (50 mM TRIS pH 7.4, 1 mM EDTA, 2 mM DTT, and 0.01% Tween-20). The rate of Mpro enzymatic activity (*v*) was determined by monitoring the increase in fluorescence intensity of reactions at room temperature in black microplates (NUNc 384-well F-bottom) with an Infinite M-100 plate reader (Tecan) using 325 and 400 nm as wavelengths for excitation and emission, respectively. Test compounds were dissolved in DMSO and screened first at a 25 μM. Three-fold serial dilutions (125 μM to 6.35 nM) of small molecule test compounds are added to determine inhibitory potency. IC_50_ is determined by an in-house evaluation tool (IC_50_ studio with 4-parametric fitting).

### Virtual Library Enumeration

The virtual libraries for docking were enumerated by using the Knime analytic platform^20^ in combination with internally developed Knime nodes which are based on the Open Chem Lib Java framework.^21^

### Molecular Docking (S2 Pocket Optimization) and Compound Selection

The crystal structure of Mpro in complex with compound **1** was solved internally and used for docking. Virtual ligands were prepared by using LigPrep module, and the protein complex structure was prepared by using Protein Preparation Wizard (Schrödinger). The low-energy conformers of the ligand were performed through the OPLS4 force field. The grid box was created according to the crystal ligand within an orthorhombic box, and H-bond constraints with H163 and G143 were selected. A template (compound **1** MCS) docking [extra-precision (XP)] satisfying both H-bond constraints was carried out with the Glide module implemented in Schrödinger 2021. Among the docking poses, the best poses obtained by comparing the interaction with key residues were visually assessed and starred (1 to 3 stars). Normalized (0 to1) MMGBSA_dG_Bind score, docking score, and visualization score (the number of stars/3) were used in a consensus score ((Norm. MMGBSA_dG_Bind score + Norm. docking score + visualization score)/3) for the selection of candidate compounds for synthesis.

### Molecular Docking (Evaluation of a Third Exit Vector) and Compound Selection

The crystal structure of Mpro in complex with X (PDB code: 7L13) was obtained from RCSB PDB (https://www.rcsb.org), and the crystal structure of Mpro in complex with compound **1** was solved internally. A hybrid complex (PDB 7L13 protein and compound **1** ligand) was formed. Virtual ligands were prepared by using the LigPrep module, and the protein complex structure was prepared by using Protein Preparation Wizard (Schrödinger). The low-energy conformers of the ligand were performed through the OPLS4 force field. The grid box was created according to the crystal ligand within an orthorhombic box, and H-bond constraints with H163 and G143 were selected. A template (compound **1** MCS) docking [extra-precision (XP)] satisfying both H-bond constraints was carried out with the Glide module implemented in Schrödinger 2021. WaterMap was run in the default mode (Schrödinger 2021-2 suite) using the hybrid complex mentioned above, and WM/MM Δ-*G* bind was calculated for each docking pose. Compounds with a produced docking pose and for which the WM/MM Δ-*G* bind values were below −32 kcal/mol were considered further.

### Molecular Dynamic (MD) Simulation (Evaluation of a Third Exit Vector)

MD boxes for each of the best docking poses of each of the five isomers were created using the System Builder module, also part of the Schrödinger 2021–2 suite. Simple point charge was chosen as the solvent model. For the box shape, orthorhombic was selected with default settings. Finally, OPLS4 was chosen as force field. The constructed system model was then imported into the MD module for extended simulation studies. The ensemble class selected for the MD simulations was *NPT* (constant number of particles, pressure, and temperature). The simulation duration was set as 100 ns, and default settings were used for the remaining parameters. To allow a post MD simulation analysis of the stability of each isomer pose, the “Run interactions analysis” box was checked. The resulting protein and ligand RMSD graph allowed for the analysis of the stability of protein–ligand complexes.

### Molecular Docking (S1 Pocket Optimization) and Compound Selection

The crystal structure of Mpro in complex with compound **38a** was solved internally and used for docking. Virtual ligands were prepared by using the LigPrep module, and the protein complex structure was prepared by using Protein Preparation Wizard (Schrödinger). The low-energy conformers of the ligand were performed through the OPLS4 force field. The grid box was created according to the crystal ligand within an orthorhombic box, and H-bond constraints with H163 and G143 were selected. A template (compound **38a** MCS) docking [extra-precision (XP)] satisfying both H-bond constraints was carried out with the Glide module implemented in Schrödinger 2021. Among the docking poses, the best poses obtained by comparing the interaction with key residues and correct position of each compound residue in their corresponding protein pocket (2-ethyl-3-bromophenyl in the S2, ethylamide in S1′ and enumerated moieties in S1) were visually assessed and starred (1 to 3 stars). WaterMap was run in the default mode (Schrödinger 2021-2 suite) using the crystal structure of Mpro in complex with compound **38a**, and WM/MM Δ-*G* bind was calculated for each selected docking pose. Finally, the hydrogen-bond strength between the nitrogen of the enumerated moiety and H163 was estimated with Jazzy.^22^

## Supporting Information Available

X-ray and crystallographic data (PDF)
LC–MS spectra of final compounds (ZIP)
Compound list (CSV)

## Abbreviations

ACE-Cl: 1-chloroethyl chloroformate
AI: artificial intelligence
ANAT: arylamine *N*-acetyltransferase
Boc: *tert*-butyloxycarbonyl
DCE: 1,2-dichloroethane
DCM: dichloromethane
DEL: DNA-encoded library
DGM: deep generative model
DMF: dimethylformamide
DIPEA: *N*,*N*-diisopropylethylamine
DMSO: dimethylsulfoxide
DMTA: design make test analysis
EtOAc: ethyl acetate
FRET: fluorescence resonance energy transfer
HATU: hexafluorophosphate azabenzotriazole tetramethyl uronium
HPLC: high-performance liquid chromatography
HRMS: high-resolution mass spectrometry
HTMC: high-throughput medicinal chemistry
HTS: high-throughput screening
LC: liquid chromatography
MCS: maximum common substructure
MD: molecular dynamics
MeCN: acetonitrile
MeOH: methanol
ML: machine learning
MMGBSA: molecular mechanics general Born surface area
MS: mass spectrometry
NaBH(OAc)_3_: sodium triacetoxyborohydride
NEt_3_: triethylamine
OPLS4: optimized potentials for liquid simulations 4
PDB: Protein Data Bank
RMSD: root-mean-square deviation
RT: room temperature
SAR: structure–activity relationship
SBDD: structure-based drug design
SFC: supercritical fluid chromatography
*t*BME: *tert*-butyl methyl ether
TFA: trifluoroacetic acid
THF: tetrahydrofuran
TLC: thin-layer chromatography
WM/MM: WaterMap MMGBSA
