# Supplementary material for: Accelerating the Hit-To-Lead Optimization of a SARS-CoV-2 Mpro Inhibitor Series by Combining High-Throughput Medicinal Chemistry and Computational Simulations
Source: J Med Chem. 2025 Apr 5;68(8):8269–94. doi: 10.1021/acs.jmedchem.4c02941 (PMC12035803; doi:10.1021/acs.jmedchem.4c02941)
Supplement: Supplementary file 1 — jm4c02941_si_001.pdf [file jm4c02941_si_001.pdf]

Accelerating the Hit-to-Lead optimization of a SARS-CoV-2 Mpro inhibitor series by combining High Throughput Medicinal Chemistry and Computational Simulations

Julien Hazemann,[†,#] Thierry Kimmerlin,[†,#,*] Aengus Mac Sweeney,[‡] Geoffroy Bourquin,[‡] Roland Lange,[‡] Daniel Ritz,[‡] Sylvia Richard-Bildstein,[†] Sylvain Regeon,[†] and Paul Czodrowski[§,*]

[†]Drug Discovery Chemistry, Idorsia Pharmaceuticals Ltd., Hegenheimermattweg 91, 4123 Allschwil, Switzerland

[‡]Drug Discovery Biology, Idorsia Pharmaceuticals Ltd., Hegenheimermattweg 91, 4123 Allschwil, Switzerland

[§]Chemistry Department, Johannes Gutenberg University, Duesbergweg 10-14, 55128 Mainz, Germany

*Corresponding authors: thierry.kimmerlin@idorsia.com, czodpaul@uni-mainz.de

Contents of supporting information

Ligand-Protein complex, Supplementary Figure S1 ....S2

Protein Expression S3

Hexahistidine SUMO-3CL^{pro} S3

DNA synthesized (Genscript) and cloned into pET29a+ S3

Storage buffer (X-ray) S4

Cloning, protein production and purification of SARS-CoV-2 3CL^{pro} S4

Protein Crystallization S4

Data collection, data processing and structure refinement S5

X-ray data processing and refinement statistics, Supplementary Table S1 ....S6

Molecular Formula Strings and associated data....................S7

References....................S7

Ligand-Protein complex

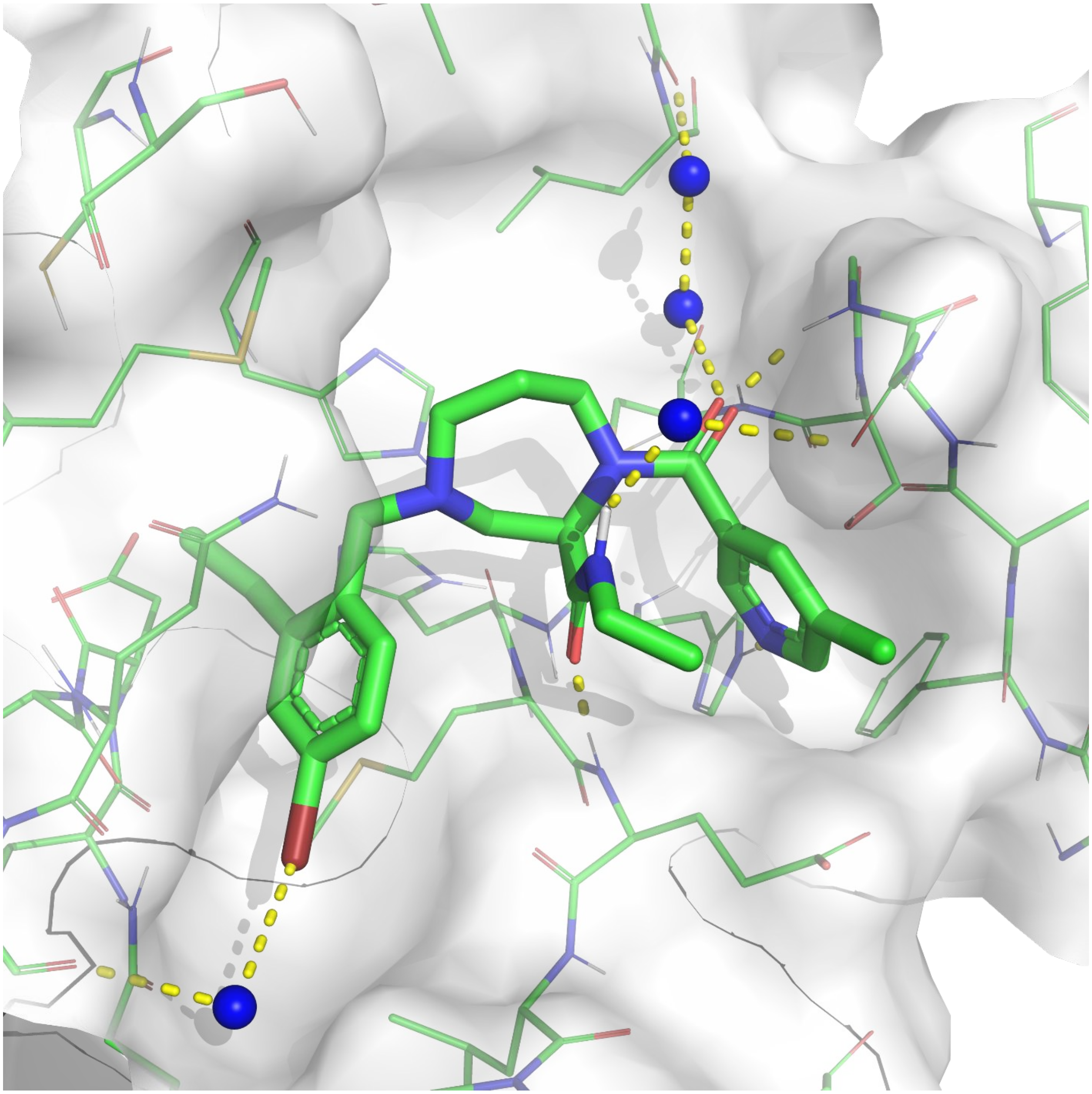

Supplementary Figure S1. Binding mode of compound 37a in Mpro (PDB code: 9HJH)

Protein Expression

SARS-CoV-2 3CL^{pro} was expressed as a Hexa-histidine-SUMO-fusion protein with amino acid sequence:

**MGSSHHHHHHGSGLVPRGSASMSDSEVDQEAKPEVKPEVKPETHINLKVSDGSSEIFFKIKKT
TPLRRLMEAFAKRQGKEMDSLRFLYDGIRIQADQTPEDLDMEDNDIIEAHREQIGGSGFRKM
AFPSGKVEGCMVQVTCGTTTLNGLWLDDVVYCPRHVICTSEDMLNPNYEDLLIRKSNHNFLV
QAGNVQLRVIGHSMQNCVLKLKVDTANPKTPKYKFVRIQPGQTFSVLACYNGSPSGVYQCAM
RPNFTIKGSFLNGSCGSVGFNIDYDCVSFCYMHHMELPTGVHAGTDLEGNFYGPFVDRQTAQ
AAGTDTTITVNVLAWLYAAVINGDRWFLNRFTTTLNDFNLVAMKYNYEPLTQDHVDILGPL
SAQTGIAVLDMCASLKELLQNGMNGRTILGSALLEDEFTPFDVVRQCSGVTFQ**

Black : Hexa-His tag and linker ; Blue: SUMO ; Red: 3CL^{pro}

Hexahistidine SUMO-3CL^{pro}

425 aas; Mol Wt 47194.9, Isoelectric Pt (pI) 5.80

Amino acid sequence “Backtranseq”; *E. coli* codon usage high

https://www.ebi.ac.uk/Tools/st/emboss_backtranseq/

DNA synthesized (Genscript) and cloned into pET29a+

CATATGGGTTCTTCTCACCACCACCACCACCACGGTTCTGGTCTGGTTCCGCGTGGTTCTGCGTC
TATGTCTGACTCTGAAGTTGACCAGGAAGCGAAACCGGAAGTTAAACCGGAAGTTAAACCGGAA
ACCCACATCAACCTGAAAGTTTCTGACGGTTCTTCTGAAATCTTCTTCAAAATCAAAAAAACCAC
CCCGCTGCGTCGTCTGATGGAAGCGTTCGCGAAACGTCAGGGTAAAGAAATGGACTCTCTGCGTT
TCCTGTACGACGGTATCCGTATCCAGGCGGACCAGACCCCGGAAGACCTGGACATGGAAGACAAC
GACATCATCGAAGCGCACCGTGAACAGATCGGTGGTTCTGGTTTCCGTAAAATGGCGTTCCCGTC
TGGTAAAGTTGAAGGTTGCATGGTTCAGGTTACCTGCGGTACCACCACCCTGAACGGTCTGTGGC
TGGACGACGTTGTTTACTGCCCGCGTCACGTTATCTGCACCTCTGAAGACATGCTGAACCCGAAC
TACGAAGACCTGCTGATCCGTAAATCTAACCACAACTTCCTGGTTCAGGCGGGTAACGTTCAGCT
GCGTGTTATCGGTCACTCTATGCAGAACTGCGTTCTGAAACTGAAAGTTGACACCGCGAACCCGA
AAACCCCGAAATACAAATTCGTTCGTATCCAGCCGGGTCAGACCTTCTCTGTTCTGGCGTGCTAC
AACGGTTCTCCGTCTGGTGTTTACCAGTGCGCGATGCGTCCGAACTTCACCATCAAAGGTTCTTT
CCTGAACGGTTCTTGCGGTTCTGTTGGTTTCAACATCGACTACGACTGCGTTTCTTTCTGCTACA
TGCACCACATGGAACTGCCGACCGGTGTTCACGCGGGTACCGACCTGGAAGGTAACTTCTACGGT
CCGTTCGTTGACCGTCAGACCGCGCAGGCGGCGGGTACCGACACCACCATCACCGTTAACGTTCT
GGCGTGGCTGTACGCGGCGGTTATCAACGGTGACCGTTGGTTCCTGAACCGTTTCACCACCACCC
TGAACGACTTCAACCTGGTTGCGATGAAATACAACTACGAACCGCTGACCCAGGACCACGTTGAC
ATCCTGGGTCCGCTGTCTGCGCAGACCGGTATCGCGGTTCTGGACATGTGCGCGTCTCTGAAAGA
ACTGCTGCAGAACGGTATGAACGGTCGTACCATCCTGGGTTCTGCGCTGCTGGAAGACGAATTCA
CCCCGTTCGACGTTGTTCGTCAGTGCTCTGGTGTTACCTTCCAGTAATAG**GGATCC**

Storage buffer (X-ray)

20 mM Tris-HCl pH 7.8, 150 mM NaCl, 1 mM TCEP, 1 mM EDTA

Cloning, protein production and purification of SARS-CoV-2 $3CL^{pro}$

DNA encoding a recombinant fusion protein (supplementary information) composed of N-terminal hexa-histidine tagged SUMO and $3CL^{pro}$ (NC_045512.2, Nsp5, YP_009742612, Wuhan-Hu-1) was codon optimized for expression in *E. coli* and synthesized (GenScript), based on the published $3CL^{pro}$ expression and crystal structure (Jin *et al.*, 2020). The synthetic DNA was cloned into pET29a (+) using the NdeI and BamHI restriction sites (GenScript) and transformed into BL21(DE3) cells. The protein was expressed overnight (Luria broth medium, 25 µg/ml Kanamycin) at 18ºC after inducing with 0.5 mM isopropyl-β-D-thiogalactoside (IPTG) at an OD_{600} of approximately 0.7. Overnight cultures were collected by centrifugation and the recovered cell paste was stored at -70ºC. Twelve grams of cell paste was resuspended in 20 mM Tris-HCl at pH 7.8, 150 mM NaCl, 5 mM imidazole and treated with lysozyme (1mg/ml; 30 min) and Benzonase (2500 Units, 10 mM $MgCl_2$; 15 min, room temperature). Bacterial cells were lysed by high pressure homogenization (29008 p.s.i. or 200 MPa, Microfluidics MP110P, DIXC H10Z) and centrifuged for 30 minutes at 16000 r.p.m. (Fiberlite F21-8×50y, maximum r.c.f. 30,392 *g*). The hexa-histidine SUMO-$3CL^{pro}$ fusion protein was purified by immobilized metal affinity chromatography (IMAC) with a HisTrap column (5 ml, Cytiva) connected to a FPLC AKTA Purifier 100 system. Histidine tagged fusion protein was eluted at a flowrate of 2 ml/min with a linear gradient of increasing imidazole concentration (from 0 to 100% elution buffer over 20 column volumes; elution buffer: 20 mM Tris-HCl pH 7.8, 150 mM NaCl, 500 mM imidazole). Eluate fractions containing the target protein were combined and concentrated (Amicon, 10 kDa cutoff). The fusion protein was treated with SUMO protease (Sigma-Aldrich SAE0067, 5 U/mg target protein) to liberate $3CL^{pro}$ with authentic N- and C-termini (Ser1 and Gln306, respectively). The mixture of cleavage products was dialyzed overnight at 4ºC using a Slide-A-Lyzer cassette (10 kDa cutoff, Thermo Scientific) in 4 l dialysis buffer (20 mM Tris-HCl, 150 mM NaCl). The histidine tagged SUMO protein was separated from non-tagged authentic $3CL^{pro}$ present in the dialysate by immobilized-metal affinity chromatography (IMAC), collecting $3CL^{pro}$ in the flow through. $3CL^{pro}$ was further purified by size exclusion chromatography (HiLoad 26/600 Superdex 200) with storage buffer (20 mM Tris-HCl, 150 mM NaCl, 1 mM TCEP, 1 mM EDTA). The elution volume of $3CL^{pro}$ indicated a dimer as the oligomeric state. $3CL^{pro}$ (97% purity by LC-MS analysis) was concentrated (Amicon, 10 kDa cutoff) to a final protein concentration of 26 mg/ml (770 µM) and stored at -70ºC.

Protein Crystallization

Aliquots of purified $3CL^{pro}$ at 26 mg/ml in storage buffer were thawed on ice and incubated with inhibitor for three hours at 20ºC at an inhibitor concentration of 10 mM (10% final DMSO concentration). Vapor diffusion crystallization trials were performed at 20 ºC using sitting drops containing 300 nl each of protein and precipitant solution (Intelli-Plate 96-2, Art Robbins). Co-crystals of Cpd-37a were grown using 25% w/v PEG3350, 100 mM HEPES pH 7.0. Co-crystals of Cpd-38a were grown using 10% w/v PEG 4000, 20% v/v glycerol, 30

mM each of sodium fluoride, sodium bromide and sodium iodide, 100 mM MES/imidazole pH 6.5 (Morpheus® condition B3). Co-crystals of Cpd-119 were grown using 100 mM MES/imidazole pH 6.5, 12.5% (w/v) PEG1000, 12.5% (w/v) PEG3350 and 12.5% (v/v) MPD (Morpheus® condition A4) as precipitant.

Data collection, data processing and structure refinement

Crystals were mounted in nylon loops and stored in liquid nitrogen. Data collection was carried out at 100K at beamline ID23-1 of the European Synchrotron Radiation Facility (ESRF), Grenoble, France. The data were processed and scaled using autoPROC[1] and XSCALE[2]. Automated molecular replacement was carried out using Dimple[3] with the 3CL^{pro} structure as template. *Coot*[4] was used for model building. Refmac[5] was used for refinement of the structures. Data collection and refinement statistics are reported in **Table S1**. Ligand restraints were generated using Grade2.[6] In structure 9HJH, an unmodelled elongated electron density peak was observed close to the non-crystallographic 2-fold axis and the side chain of Lys5 of both protein chains.

X-ray data processing and refinement statistics

		Cpd-37a	Cpd-38a	Cpd-119
Data Processing				
Source		ESRF ID23-1	ESRF ID23-1	ESRF ID23-1
Space Group		$P2_12_12_1$	$P2_12_12_1$	$P2_12_12_1$
Wavelength (Å)		0.89	0.89	0.89
Cell dimensions	a, b, c (Å)	68.0, 101.5, 104.4	68.0, 99.7, 104.1	67.8, 100.2, 104.3
Observed reflections		2185989	1648592	1983773
Unique reflections		223287	183140	192867
Resolution (highest shell) (Å)		56.8-1.197 (1.218-1.197)	57.0-1.277 (1.299-1.277)	56.9-1.250 (1.271-1.250)
Rpim (%)		4.90 (36.7)	4.80 (100.1)	3.6 (68.8)
Rmerge (%)		10.1 (72.8)	13.6 (277.0)	11.1 (212.5)
Mean I/σ(I)		11.3 (2.3)	8.2 (0.7)	8.4 (0.9)
Completeness (%)		99.9 (98.2)	99.8 (98.1)	99.6 (53.2)
Redundancy		9.9 (9.3)	9.0 (8.2)	10.3 (10.2)
$CC_{0.5}$		0.996 (0.874)	0.997 (0.371)	0.996 (0.532)
Structure Refinement				
Resolution (highest shell) (Å)		56.9-1.20 (1.23-1.20)	41.4-1.28 (1.31-1.28)	56.9-1.25 (1.28-1.25)
R_{work} (highest shell) (%)		15.0 (21.9)	16.4 (36.9)	16.0 (34.4)
R_{free} (highest shell) (%)		15.9 (21.5)	19.2 (36.5)	17.6 (33.0)
B-factors (Å^2) (number of non-H atoms)	Protein	13.9 (4826)	16.2 (4758)	20.2 (4796)
	Ligands	13.9 (78) Cpd-37a	16.8 (62) Cpd-38a<br>18.5 (4) DMSO	18.5 (66) Cpd-119
	Water	27.8 (688)	29.3 (644)	33.8 (604)
Rms deviation	Bond lengths (Å)	0.008	0.008	0.005
	Bond angles (˚)	1.54	1.56	1.38
Ramachandran plot (%)	Favoured	98.3	97.8	98.0
	Allowed	1.5	2.0	1.8
	Disallowed	0.2 (Tyr154, chain B)	0.2 (Tyr154, chain B)	0.2 (Tyr154, chain B)
PBD entry ID		9HJH	9HAJ	9HAK

Supplementary Table S1. Data collection and refinement statistics for Mpro in complex with Cpd-37a, Cpd-38a and Cpd-119.

Molecular Formula Strings and associated data

A separate CSV file is available.

References

1. Vonrhein, C, Flensburg, C, Keller, P, et al. (2011). Acta Cryst. D67, 293–302
2. Kabsch, W (2010). Acta Cryst. D66, 125–132
3. Wojdyr, M., Keegan, R., Winter, G., Ashton, A. (2013) DIMPLE - a pipeline for the rapid generation of difference maps from protein crystals with putatively bound ligands. Acta Cryst. A69: s299
4. Emsley, P, Lohkamp, B, Scott, WG, Cowtan, K. (2010). Acta Cryst. D66, 486–501
5. Murshudov, GN, Skubák, P, Lebedev, AA, et al. (2011). Acta Cryst. D67, 355–367
6. Smart, OS, Womack, TO, Sharff, A, et al. (2011). Grade2, version 1.4.0. Cambridge: Global Phasing
