# Supplementary material for: Accelerating the Hit-To-Lead Optimization of a SARS-CoV-2 Mpro Inhibitor Series by Combining High-Throughput Medicinal Chemistry and Computational Simulations
Source: J Med Chem. 2025 Apr 5;68(8):8269–94. doi: 10.1021/acs.jmedchem.4c02941 (PMC12035803; doi:10.1021/acs.jmedchem.4c02941)
Supplement: Supplementary file 2 — jm4c02941_si_002.zip [file jm4c02941_si_002.zip › cpd 91.pdf]

idorsia **HTS QC Analysis Report**

Sample Name: LCMSH384-20241023-1_M05
ACT No: IDOR-1149-2621
Instrument:
ELN Nr: EXT-1082-3617
Acquisition time: 10/23/2024 3:55 PM

Expression	Result
Auto-Comments	
Auto-Summary	GOOD
Purity	98.2000
Target Mass	578.1966
Target RT	0.8180

UV[212-216] UV214 [7.1E06]

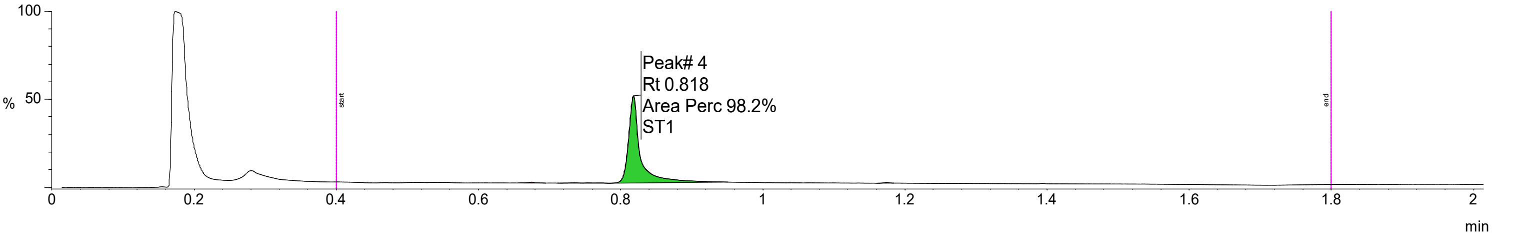

cid=6 576.1969 (576.1469-576.2469) w=0.1000 T1-EIC(+)[M+H] [33436.7]

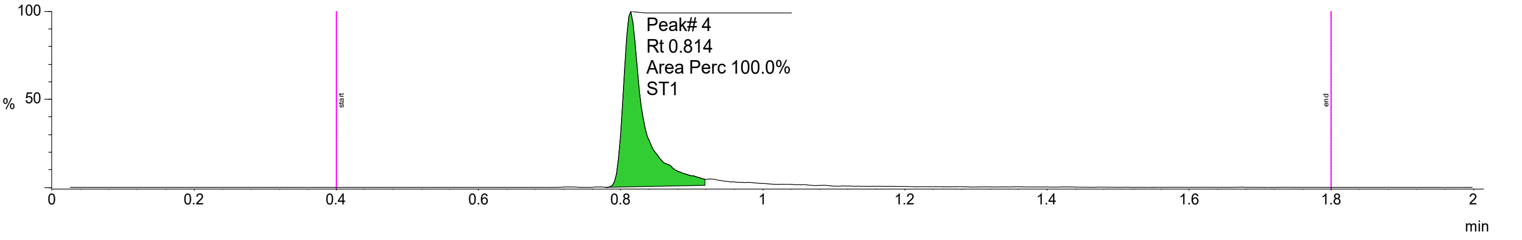

Peak 1@0.675 min MS(+) ES **2905.2 cnts**

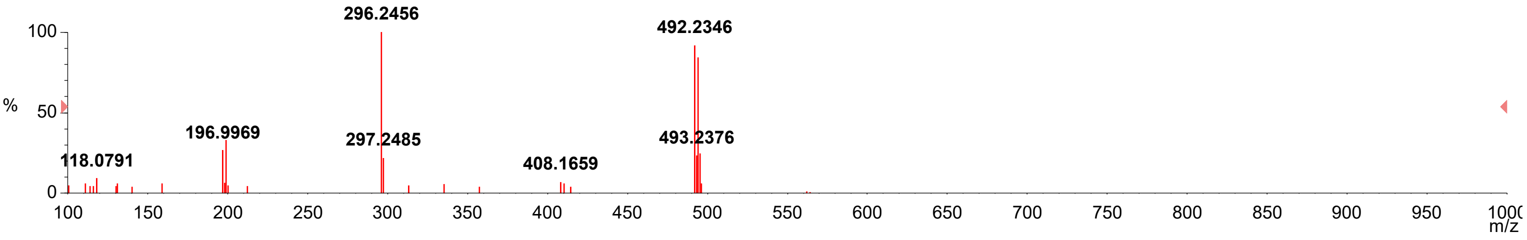

Peak 2@0.735 min MS(+) ES **884.5 cnts**

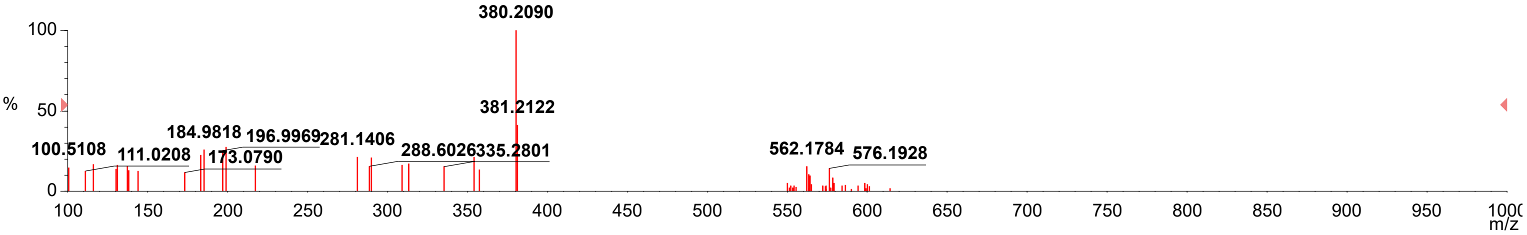

Peak 4@0.814 min MS(+) ES **59481.7 cnts**

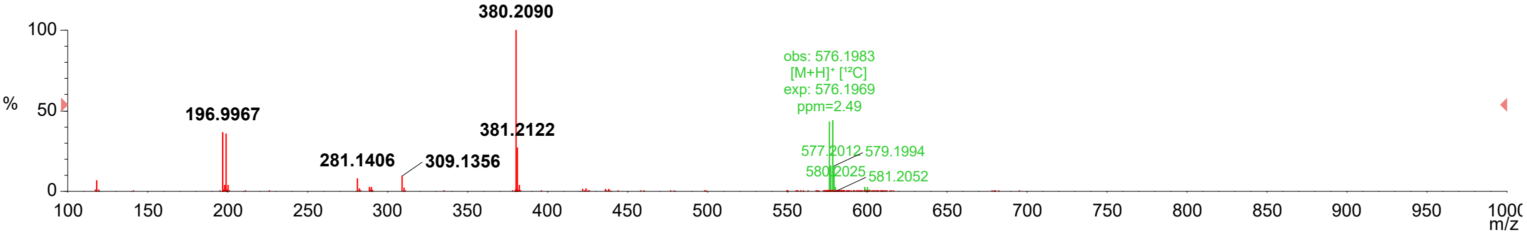

HTS QC Analysis Report

Sample Name: LCMSH384-20241023-1_M05
ACT No: IDOR-1149-2621
ELN Nr: EXT-1082-3617
Instrument:
Acquisition time: 10/23/2024 3:55 PM

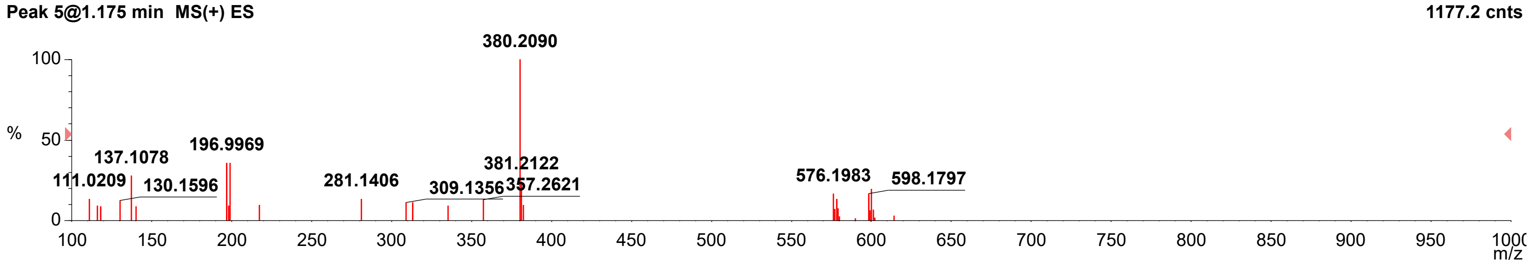

idorsia **HTS QC Analysis Report**

Sample Name: LCMSH384-20241023-1_M05 **ACT No:** IDOR-1149-2621 **ELN Nr:** EXT-1082-3617
Instrument: **Acquisition time:** 10/23/2024 3:55 PM

```
ACE Experimental Record


Inlet Method File: d:\masslynx projects\hts.pro\acqudb\acid_fa

---------------------   Run method parameters   ----------------

-- PUMP --

Waters Acquity SDS
 Run Time: 2.00 min
 Comment: 
 Solvent Selection A: A1
 Solvent Selection B: B1
 Low Pressure Limit: 0.000 bar
 High Pressure Limit: 1034.200 bar
 Solvent Name A: H20 + 0.05% FA
 Solvent Name B: ACN + 0.05%FA
 Switch 1: No Change
 Switch 2: No Change
 Switch 3: No Change
 Seal Wash: 5.0 min
 Chart Out 1: System Pressure
 Chart Out 2: %B
 System Pressure Data Channel: Yes
 Flow Rate Data Channel: No
 %A Data Channel: No
 %B Data Channel: Yes
 Primary A Pressure Data Channel: No
 Accumulator A Pressure Data Channel: No
 Primary B Pressure Data Channel: No
 Accumulator B Pressure Data Channel: No
 Degasser Pressure Data Channel: No
 [Gradient Table]
  Time(min) Flow Rate %A %B Curve
 1. Initial 1.000 98.0 2.0 Initial
 2. 1.50 1.000 5.0 95.0 6
 3. 1.90 1.000 5.0 95.0 6
 4. 1.95 1.000 98.0 2.0 6
 Run Events: Yes
 Gradient Start (Relative to Injection): 0 uL
 2D Repeat: No

-- END PUMP --

-- DETECTOR --

Waters Acquity PDA

 Run Time: 2.00 min
 PDA Detector Type: UPLC LG 500 nm
 Lamp: On
 Sampling Rate: 20 points/sec
 Filter Time Constant: 0.1000 sec
 Exposure Time: Auto msec
 Interpolate 2nd order filter Region: No
 Use UV Blocking Filter: No
3D Channel...
 Range: 190  - 400
 Resolution: 1.2 nm
Analog 1...
 2D Channel: 8
 Full Scale Range: 2.000 units
 Full Scale Voltage: 2000 mV
 Voltage Offset: 0 mV
Analog 2...
 2D Channel: 8
 Full Scale Range: 2.000 units
 Full Scale Voltage: 2000 mV
 Voltage Offset: 0 mV
 Initial Switch 1: No Change


-- END DETECTOR --

-- DETECTOR --
```

```
Waters Acquity CM

 Target Column Temperature: 60.0 C
 Temperature Alarm Band: 5.0 C
 Shutdown all columns: No
 Column Valve Position: Column 1
 Equilibration Time: 1.0 min
 Active Preheater: Enabled
 External Valve 1: No Change
 External Valve 2: No Change
 External Valve 3: No Change
 Comment:
 Column Temperature Data Channel: No
 Preheater Temperature Data Channel: No


-- END DETECTOR --

-- AUTOSAMPLER --

Waters Acquity AutoSampler
 Run Time: 2.00 min
 Comment:
 Load Ahead: Enabled
 Injection Mode: Partial Loop
 LoopOffline: 0.10
 Weak Wash Solvent Name: H2O/ACN 90/10 v/v
 Weak Wash Volume: 500 uL
 Strong Wash Solvent Name: H2O/ACN 90/10 v/v
 Strong Wash Volume: 500 uL
 Target Column Temperature: Off C
 Column Temperature Alarm Band: Disabled
 Target Sample Temperature: 20.0 C
 Sample Temperature Alarm Band: Disabled
 Full Loop Overfill Factor: Automatic
 Syringe Draw Rate: Automatic
 Needle Placement: Automatic
 Pre-Aspirate Air Gap: Automatic
 Post-Aspirate Air Gap: Automatic
 Column Temperature Data Channel: No
 Ambient Temperature Data Channel: No
 Sample Temperature Data Channel: No
 Sample Organizer Temperature Data Channel: No
 Sample Pressure Data Channel: No
 PreheaterTemperatureDataEnable: false
 Switch 1: No Change
 Switch 2: No Change
 Switch 3: No Change
 Switch 4: No Change
 Chart Out: Sample Pressure
 Sample Temp Alarm: Disabled
 Column Temp Alarm: Disabled
 Run Events: Yes
 Column Valve Position: Column 1
 Column Manager Temperature: Off C
 Column Equilibration Time: 0.1 min
 Column Manager Temp Data Channel: No
 Column Manager Temp Alarm Band: 5.0 C
 Needle Overfill Flush: Automatic
 NoInjection: false
 Active Preheater: Use Console Configuration


Sample Run Injection Parameter

Injection Volume (ul)   -   0.20
-- END AUTOSAMPLER --

----------------------------  oOo  -----------------------------


End of experimental record.------------------- Waters Acquity SM Postrun Report  ---------------



 Software Version: 1.65.3163
```

idorsia **HTS QC Analysis Report**

Sample Name: LCMSH384-20241023-1_M05 **ACT No:** IDOR-1149-2621 **ELN Nr:** EXT-1082-3617
Instrument: **Acquisition time:** 10/23/2024 3:55 PM

```
 Firmware Version: 1.65.375 (Mar 26 2015)

 Checksum: 0x42c0e8cb

 Serial Number: D09UPA925M

 Sample Syringe Size: 100.0

 Sample Loop Size: 2.0

 Needle Size: 15.0

 Minimum Sample Temperature: 20.0

 Maximum Sample Temperature: 20.0

 Average Sample Temperature: 20.0

 Minimum Column Temperature: 0.0

 Maximum Column Temperature: 0.0

 Average Column Temperature: 0.0

 Measured Loop Volume: 0.000

 Measured Loop Volume No Pressure: 0.000

----------------------------  oOo  -----------------------------


------------------- Waters Acquity SDS Postrun Report  ---------------



 IcsVersion: 1.65.2001

 FirmwareVersion: 1.65.273 (Feb 27 2015)

 Checksum: 0x3462a3f3

 SerialNumber: K08UPB301M

 Minimum System Pressure: 379.6

 Maximum System Pressure: 767.1

 Average System Pressure: 598.1

 Minimum Degasser Pressure: 0.0

 Maximum Degasser Pressure: 0.0

 Average Degasser Pressure: 0.0

----------------------------  oOo  -----------------------------
------------------- Generic Instrument Postrun Report  ---------------

 Software Version: 1.65.1503

 Firmware Version: 1.65.6227 (May 07 2015)

 Checksum: 0xf7835e17

 Serial Number: A09UPD396M

 Lamp On/Off Event: No

 Lamp Life: 166.00 hours

 Lamp Serial Number: 000349378
```

HTS QC Analysis Report

Sample Name: LCMSH384-20241023-1_M05 **ACT No:** IDOR-1149-2621 **ELN Nr:** EXT-1082-3617
Instrument: **Acquisition time:** 10/23/2024 3:55 PM

Exposure Time: 25.000 msec

Lambda1: 186.984

Lambda512: 502.356

---------------------------- oOo -----------------------------

------------------- Waters Acquity CM Postrun Report ---------------

Software Version: 1.65.2263

Firmware Version: 1.40.74 (Oct 27 2011)

Checksum: 0x8e9d1dc0

Serial Number: G07UPM471M

Valve Position: 1

ColumnType: ACQUITY UPLC□ CSH□ C18 1.7□m

Column Serial Number: 01753135515176

Column Part Number: 186005296

Total Injections on Column: 1516

Minimum Column Temperature: 60.0 C

Maximum Column Temperature: 60.0 C

Average Column Temperature: 60.0 C

---------------------------- oOo -----------------------------
