# Supplementary material for: Accelerating the Hit-To-Lead Optimization of a SARS-CoV-2 Mpro Inhibitor Series by Combining High-Throughput Medicinal Chemistry and Computational Simulations
Source: J Med Chem. 2025 Apr 5;68(8):8269–94. doi: 10.1021/acs.jmedchem.4c02941 (PMC12035803; doi:10.1021/acs.jmedchem.4c02941)
Supplement: Supplementary file 2 — jm4c02941_si_002.zip [file jm4c02941_si_002.zip › cpd 104.pdf]

idorsia HTS QC Analysis Report

Sample Name: LCMSH384-20241023-1_E07 **ACT No:** IDOR-1149-2622 **Instrument:** UPLC_SYNAPT1 **ELN Nr:** EXT-1082-3618 **Acquisition time:** 10/23/2024 2:53 PM

Expression	Result
Auto-Comments	Possible Rotamers
Auto-Summary	GOOD
Purity	99.9000
Target Mass	585.1459
Target RT	1.0810

UV[212-216] UV214 [7.1E06]

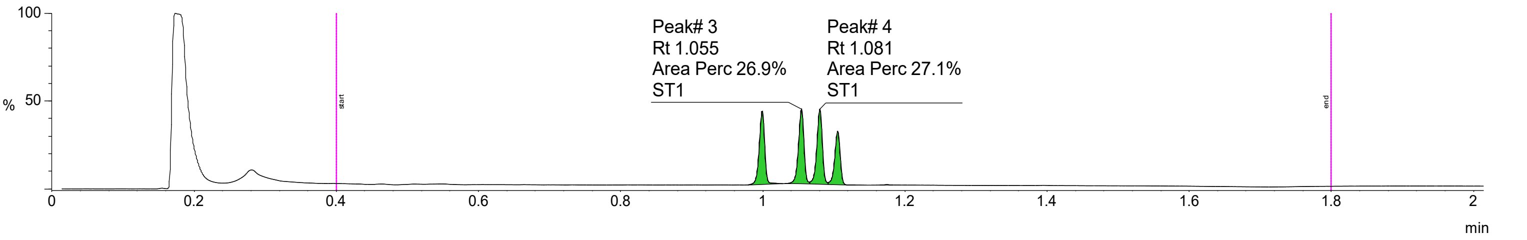

cid=6 583.1470 (583.0970-583.1970) w=0.1000 T1-EIC(+)[M+H] [37313.6]

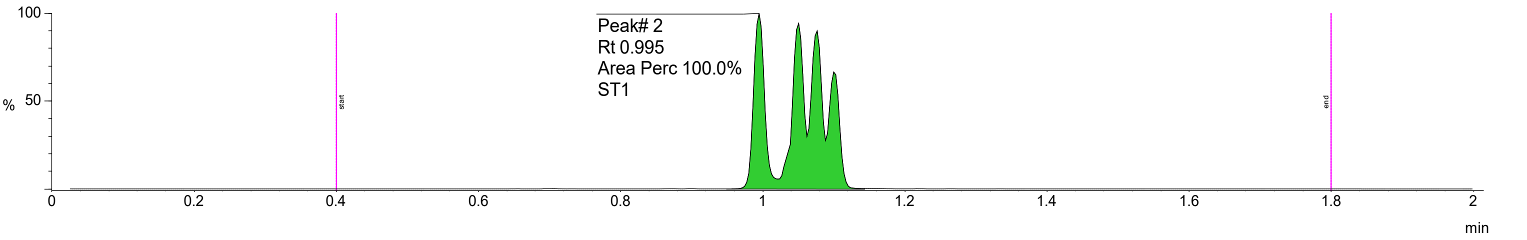

Peak 2@0.995 min MS(+) ES **39489.0 cnts**

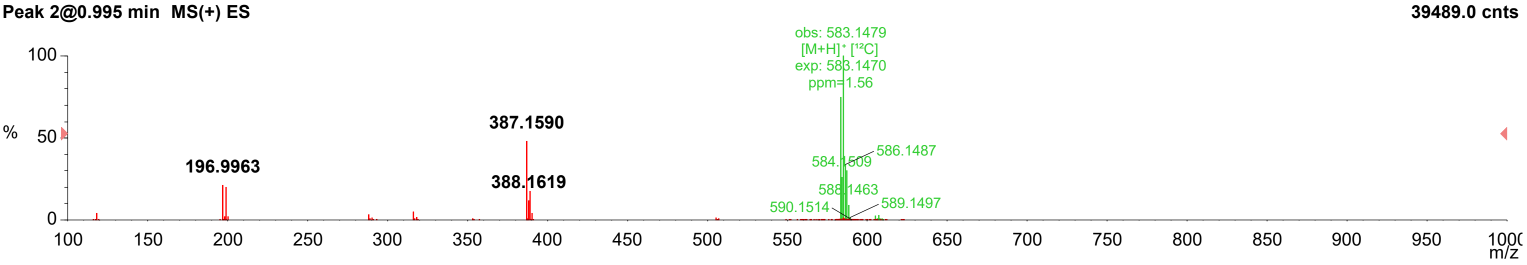

Peak 3@1.055 min MS(+) ES **42964.8 cnts**

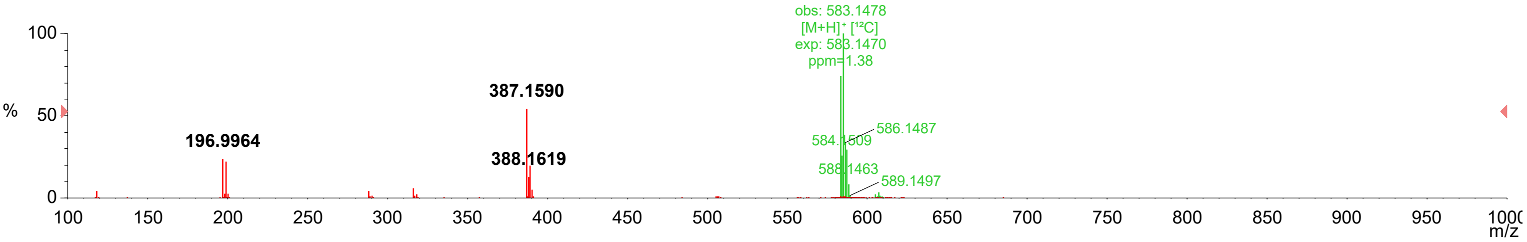

Peak 4@1.081 min MS(+) ES **39182.4 cnts**

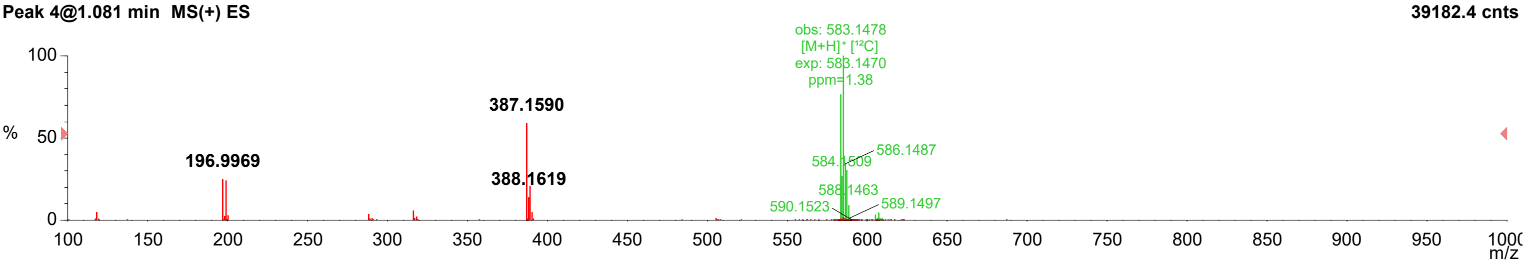

HTS QC Analysis Report

Sample Name: LCMSH384-20241023-1_E07 **ACT No:** IDOR-1149-2622 **ELN Nr:** EXT-1082-3618
Instrument: UPLC_SYNAPT1 **Acquisition time:** 10/23/2024 2:53 PM

Peak 5@1.106 min MS(+) ES **26412.2 cnts**

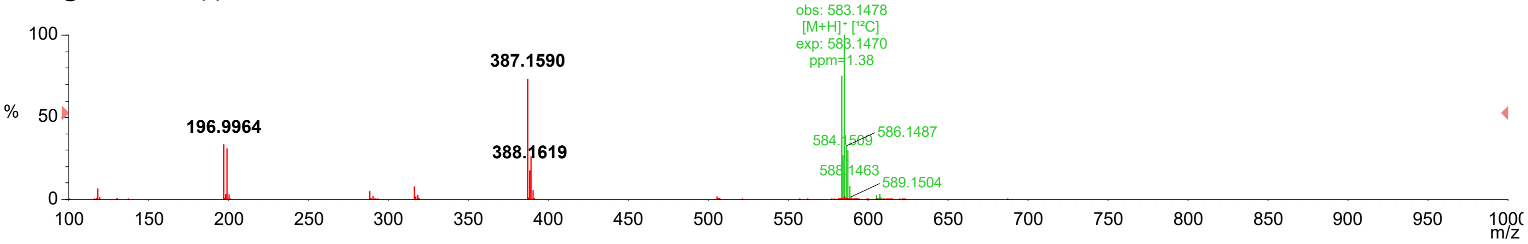

HTS QC Analysis Report

Sample Name: LCMSH384-20241023-1_E07 **ACT No:** IDOR-1149-2622 **ELN Nr:** EXT-1082-3618
Instrument: UPLC_SYNAPT1 **Acquisition time:** 10/23/2024 2:53 PM

```
ACE Experimental Record


Inlet Method File: d:\masslynx projects\hts.pro\acqudb\acid_fa

---------------------   Run method parameters   ----------------

-- PUMP --

Waters Acquity SDS
 Run Time: 2.00 min
 Comment:
 Solvent Selection A: A1
 Solvent Selection B: B1
 Low Pressure Limit: 0.000 bar
 High Pressure Limit: 1034.200 bar
 Solvent Name A: H20 + 0.05% FA
 Solvent Name B: ACN + 0.05%FA
 Switch 1: No Change
 Switch 2: No Change
 Switch 3: No Change
 Seal Wash: 5.0 min
 Chart Out 1: System Pressure
 Chart Out 2: %B
 System Pressure Data Channel: Yes
 Flow Rate Data Channel: No
 %A Data Channel: No
 %B Data Channel: Yes
 Primary A Pressure Data Channel: No
 Accumulator A Pressure Data Channel: No
 Primary B Pressure Data Channel: No
 Accumulator B Pressure Data Channel: No
 Degasser Pressure Data Channel: No
 [Gradient Table]
  Time(min) Flow Rate %A %B Curve
 1. Initial 1.000 98.0 2.0 Initial
 2. 1.50 1.000 5.0 95.0 6
 3. 1.90 1.000 5.0 95.0 6
 4. 1.95 1.000 98.0 2.0 6
 Run Events: Yes
 Gradient Start (Relative to Injection): 0 uL
 2D Repeat: No

-- END PUMP --

-- DETECTOR --

Waters Acquity PDA

 Run Time: 2.00 min
 PDA Detector Type: UPLC LG 500 nm
 Lamp: On
 Sampling Rate: 20 points/sec
 Filter Time Constant: 0.1000 sec
 Exposure Time: Auto msec
 Interpolate 2nd order filter Region: No
 Use UV Blocking Filter: No
3D Channel...
 Range: 190  - 400
 Resolution: 1.2 nm
Analog 1...
 2D Channel: 8
 Full Scale Range: 2.000 units
 Full Scale Voltage: 2000 mV
 Voltage Offset: 0 mV
Analog 2...
 2D Channel: 8
 Full Scale Range: 2.000 units
 Full Scale Voltage: 2000 mV
 Voltage Offset: 0 mV
 Initial Switch 1: No Change


-- END DETECTOR --

-- DETECTOR --

Waters Acquity CM
```

HTS QC Analysis Report

Sample Name: LCMSH384-20241023-1_E07 **ACT No:** IDOR-1149-2622 **ELN Nr:** EXT-1082-3618
Instrument: UPLC_SYNAPT1 **Acquisition time:** 10/23/2024 2:53 PM

```
 Target Column Temperature: 60.0 C
 Temperature Alarm Band: 5.0 C
 Shutdown all columns: No
 Column Valve Position: Column 1
 Equilibration Time: 1.0 min
 Active Preheater: Enabled
 External Valve 1: No Change
 External Valve 2: No Change
 External Valve 3: No Change
 Comment:
 Column Temperature Data Channel: No
 Preheater Temperature Data Channel: No


-- END DETECTOR --

-- AUTOSAMPLER --

Waters Acquity AutoSampler
 Run Time: 2.00 min
 Comment:
 Load Ahead: Enabled
 Injection Mode: Partial Loop
 LoopOffline: 0.10
 Weak Wash Solvent Name: H2O/ACN 90/10 v/v
 Weak Wash Volume: 500 uL
 Strong Wash Solvent Name: H2O/ACN 90/10 v/v
 Strong Wash Volume: 500 uL
 Target Column Temperature: Off C
 Column Temperature Alarm Band: Disabled
 Target Sample Temperature: 20.0 C
 Sample Temperature Alarm Band: Disabled
 Full Loop Overfill Factor: Automatic
 Syringe Draw Rate: Automatic
 Needle Placement: Automatic
 Pre-Aspirate Air Gap: Automatic
 Post-Aspirate Air Gap: Automatic
 Column Temperature Data Channel: No
 Ambient Temperature Data Channel: No
 Sample Temperature Data Channel: No
 Sample Organizer Temperature Data Channel: No
 Sample Pressure Data Channel: No
 PreheaterTemperatureDataEnable: false
 Switch 1: No Change
 Switch 2: No Change
 Switch 3: No Change
 Switch 4: No Change
 Chart Out: Sample Pressure
 Sample Temp Alarm: Disabled
 Column Temp Alarm: Disabled
 Run Events: Yes
 Column Valve Position: Column 1
 Column Manager Temperature: Off C
 Column Equilibration Time: 0.1 min
 Column Manager Temp Data Channel: No
 Column Manager Temp Alarm Band: 5.0 C
 Needle Overfill Flush: Automatic
 NoInjection: false
 Active Preheater: Use Console Configuration


Sample Run Injection Parameter

Injection Volume (ul)   -   0.20
-- END AUTOSAMPLER --

----------------------------  oOo  -----------------------------


End of experimental record.

------------------- Waters Acquity SDS Postrun Report  ---------------



 IcsVersion: 1.65.2001
```

HTS QC Analysis Report

Sample Name: LCMSH384-20241023-1_E07 **ACT No:** IDOR-1149-2622 **ELN Nr:** EXT-1082-3618
Instrument: UPLC_SYNAPT1 **Acquisition time:** 10/23/2024 2:53 PM

FirmwareVersion: 1.65.273 (Feb 27 2015)

Checksum: 0x3462a3f3

SerialNumber: K08UPB301M

Minimum System Pressure: 377.7

Maximum System Pressure: 768.1

Average System Pressure: 598.8

Minimum Degasser Pressure: 0.0

Maximum Degasser Pressure: 0.0

Average Degasser Pressure: 0.0

---------------------------- oOo -----------------------------

------------------- Waters Acquity SM Postrun Report ---------------

Software Version: 1.65.3163

Firmware Version: 1.65.375 (Mar 26 2015)

Checksum: 0x42c0e8cb

Serial Number: D09UPA925M

Sample Syringe Size: 100.0

Sample Loop Size: 2.0

Needle Size: 15.0

Minimum Sample Temperature: 20.0

Maximum Sample Temperature: 20.0

Average Sample Temperature: 20.0

Minimum Column Temperature: 0.0

Maximum Column Temperature: 0.0

Average Column Temperature: 0.0

Measured Loop Volume: 0.000

Measured Loop Volume No Pressure: 0.000

---------------------------- oOo -----------------------------

------------------- Generic Instrument Postrun Report ---------------

Software Version: 1.65.1503

Firmware Version: 1.65.6227 (May 07 2015)

Checksum: 0xf7835e17

Serial Number: A09UPD396M

Lamp On/Off Event: No

Lamp Life: 164.00 hours

Lamp Serial Number: 000349378

Exposure Time: 25.000 msec

Lambda1: 186.984

HTS QC Analysis Report

Sample Name: LCMSH384-20241023-1_E07 **ACT No:** IDOR-1149-2622 **Instrument:** UPLC_SYNAPT1 **ELN Nr:** EXT-1082-3618 **Acquisition time:** 10/23/2024 2:53 PM

Lambda512: 502.356

---------------------------- oOo -----------------------------

------------------- Waters Acquity CM Postrun Report ---------------

Software Version: 1.65.2263

Firmware Version: 1.40.74 (Oct 27 2011)

Checksum: 0x8e9d1dc0

Serial Number: G07UPM471M

Valve Position: 1

ColumnType: ACQUITY UPLC□ CSH□ C18 1.7□m

Column Serial Number: 01753135515176

Column Part Number: 186005296

Total Injections on Column: 1499

Minimum Column Temperature: 60.0 C

Maximum Column Temperature: 60.0 C

Average Column Temperature: 60.0 C

---------------------------- oOo -----------------------------
