# Supplementary figures and images for: Accelerating the Hit-To-Lead Optimization of a SARS-CoV-2 Mpro Inhibitor Series by Combining High-Throughput Medicinal Chemistry and Computational Simulations
Source: J Med Chem. 2025 Apr 5;68(8):8269–94. doi: 10.1021/acs.jmedchem.4c02941 (PMC12035803; doi:10.1021/acs.jmedchem.4c02941)

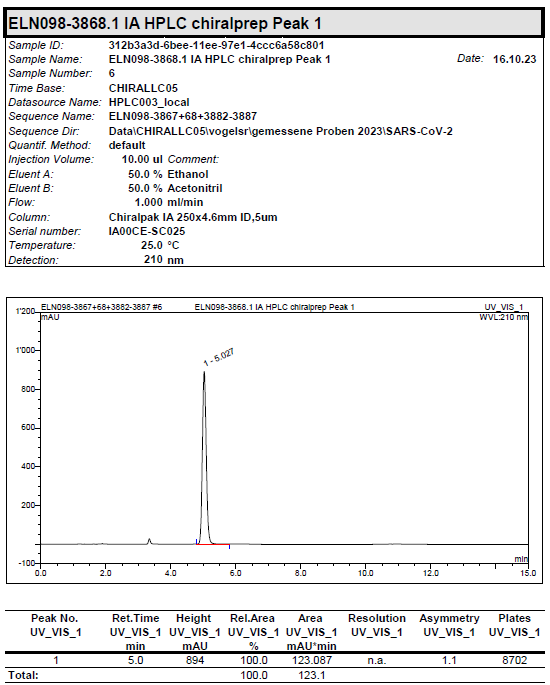

Supplement: Supplementary file 2 — jm4c02941_si_002.zip [file jm4c02941_si_002.zip › cpd 112.png]

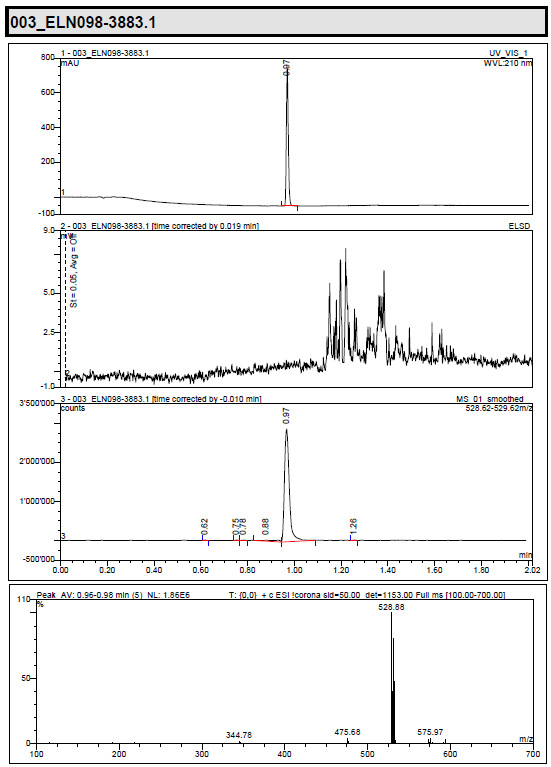

Supplement: Supplementary file 2 — jm4c02941_si_002.zip [file jm4c02941_si_002.zip › cpd 116.png]

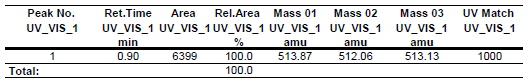

Supplement: Supplementary file 2 — jm4c02941_si_002.zip [file jm4c02941_si_002.zip › cpd 117 mass.png]

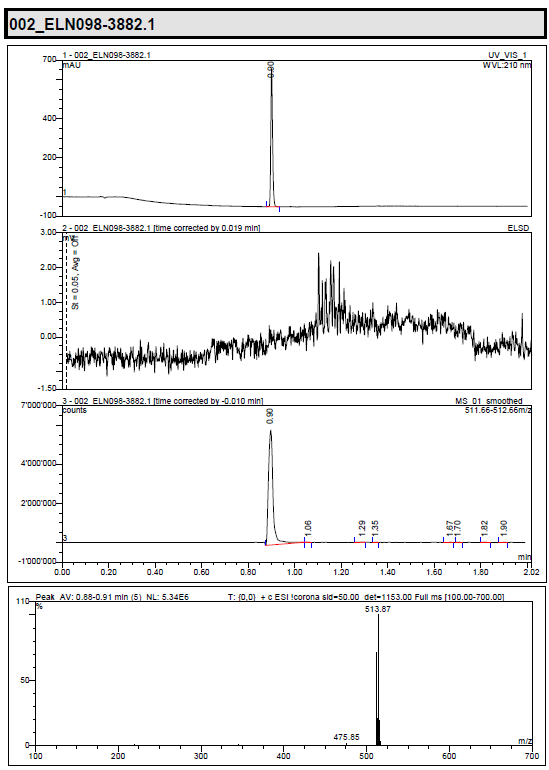

Supplement: Supplementary file 2 — jm4c02941_si_002.zip [file jm4c02941_si_002.zip › cpd 117.png]

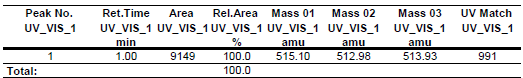

Supplement: Supplementary file 2 — jm4c02941_si_002.zip [file jm4c02941_si_002.zip › cpd 118 mass.png]

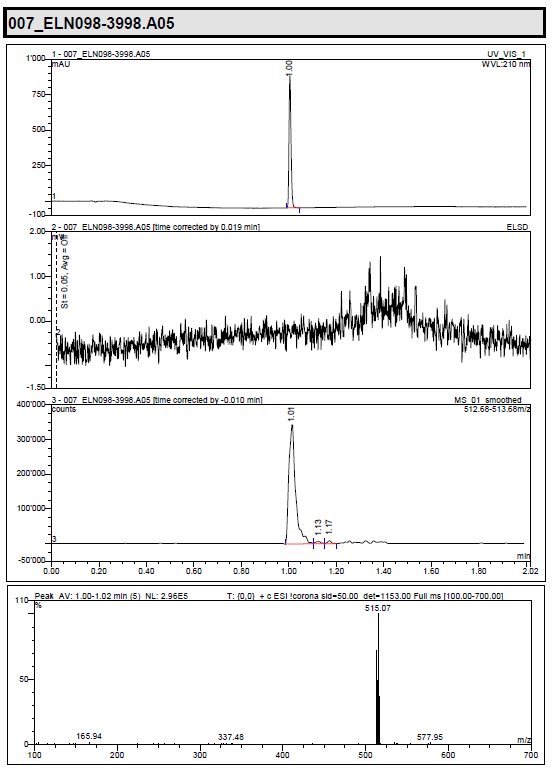

Supplement: Supplementary file 2 — jm4c02941_si_002.zip [file jm4c02941_si_002.zip › cpd 118.png]

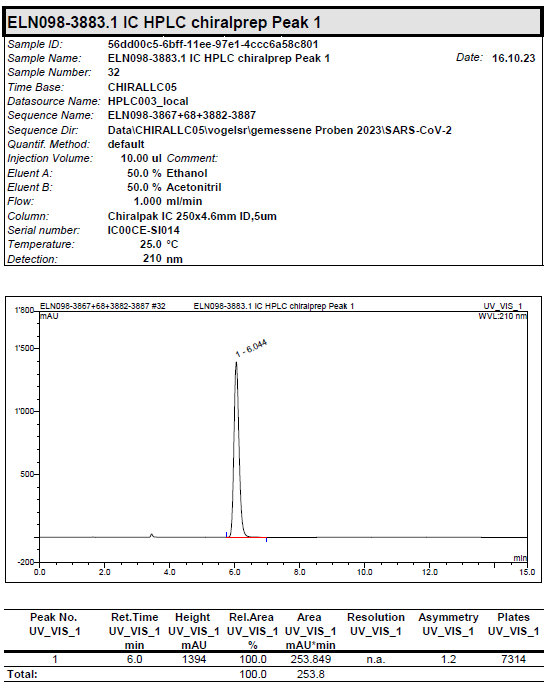

Supplement: Supplementary file 2 — jm4c02941_si_002.zip [file jm4c02941_si_002.zip › cpd 119.png]

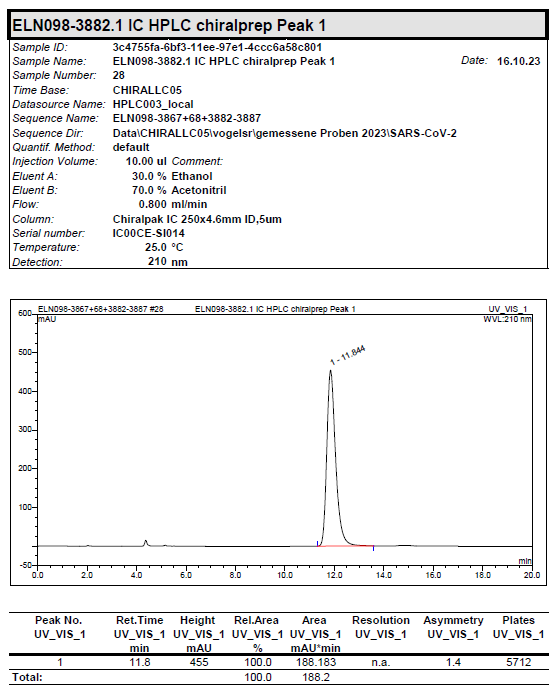

Supplement: Supplementary file 2 — jm4c02941_si_002.zip [file jm4c02941_si_002.zip › cpd 121.png]

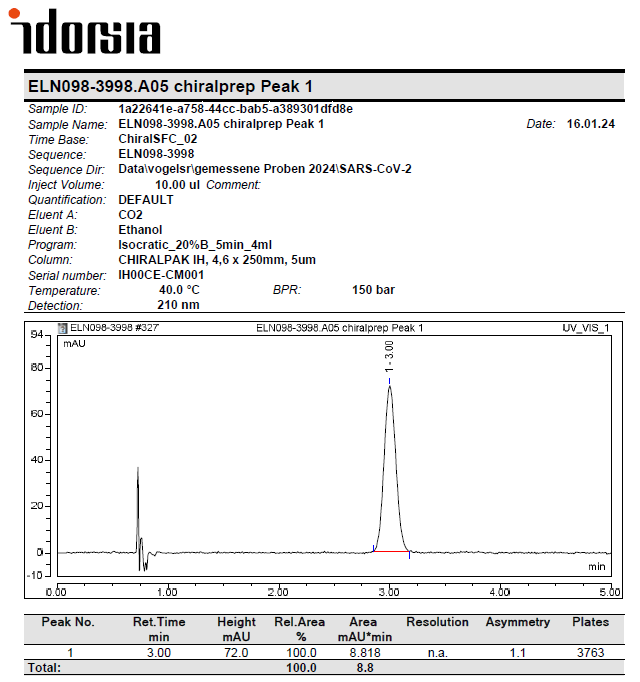

Supplement: Supplementary file 2 — jm4c02941_si_002.zip [file jm4c02941_si_002.zip › cpd 123.png]

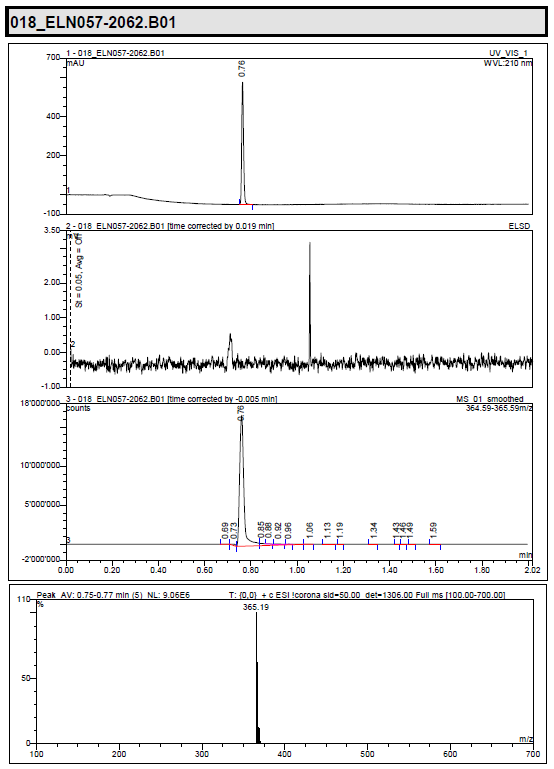

Supplement: Supplementary file 2 — jm4c02941_si_002.zip [file jm4c02941_si_002.zip › cpd 1.png]

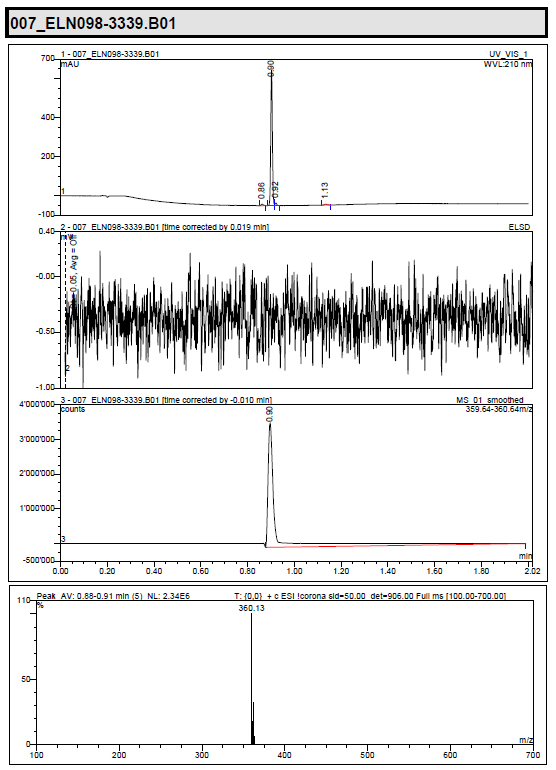

Supplement: Supplementary file 2 — jm4c02941_si_002.zip [file jm4c02941_si_002.zip › cpd 3.png]

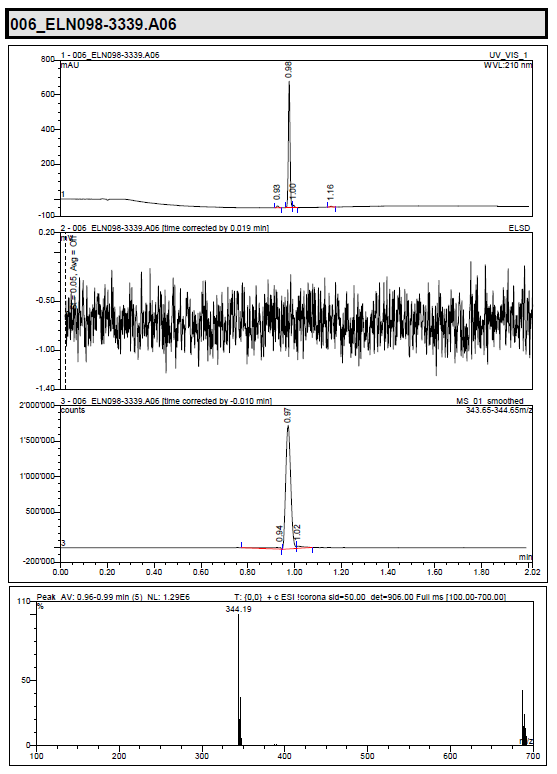

Supplement: Supplementary file 2 — jm4c02941_si_002.zip [file jm4c02941_si_002.zip › cpd 4.png]

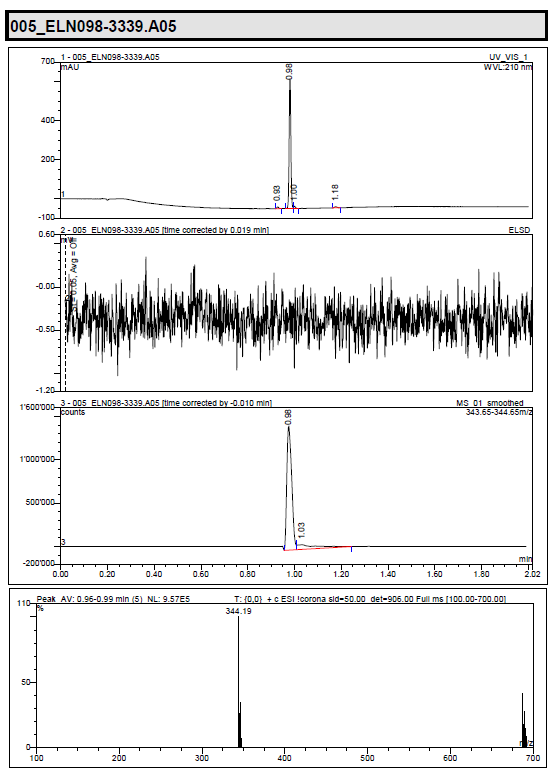

Supplement: Supplementary file 2 — jm4c02941_si_002.zip [file jm4c02941_si_002.zip › cpd 5.png]

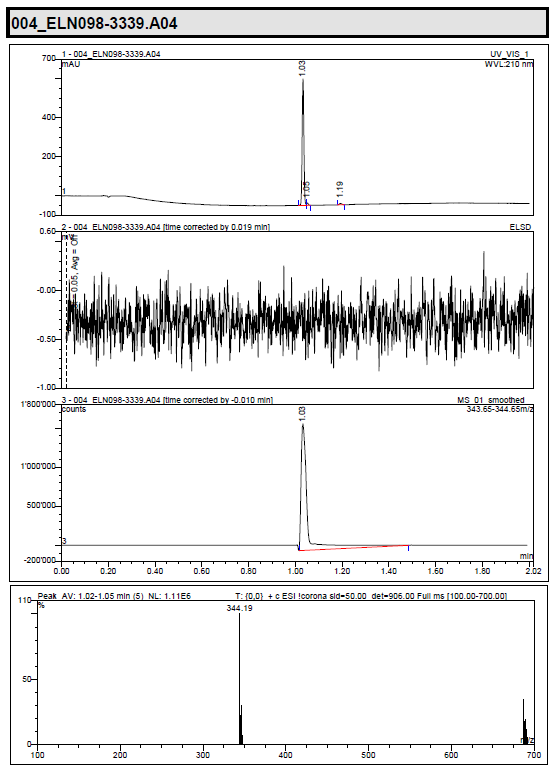

Supplement: Supplementary file 2 — jm4c02941_si_002.zip [file jm4c02941_si_002.zip › cpd 6.png]

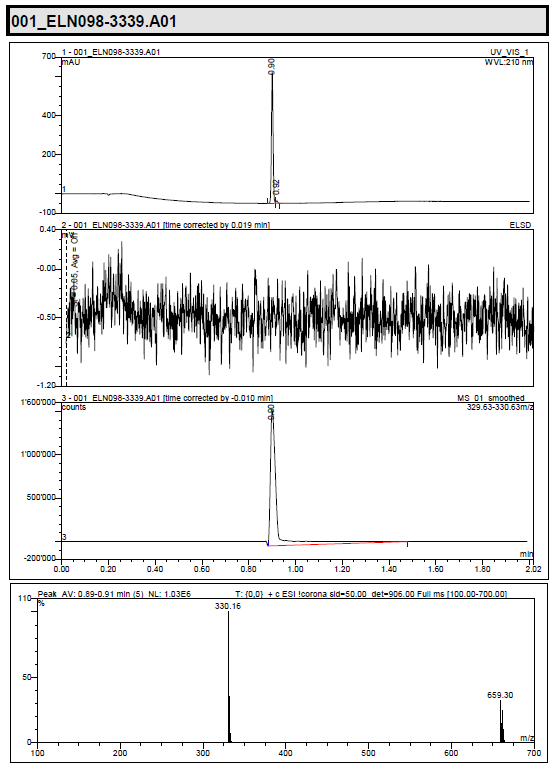

Supplement: Supplementary file 2 — jm4c02941_si_002.zip [file jm4c02941_si_002.zip › cpd 7.png]

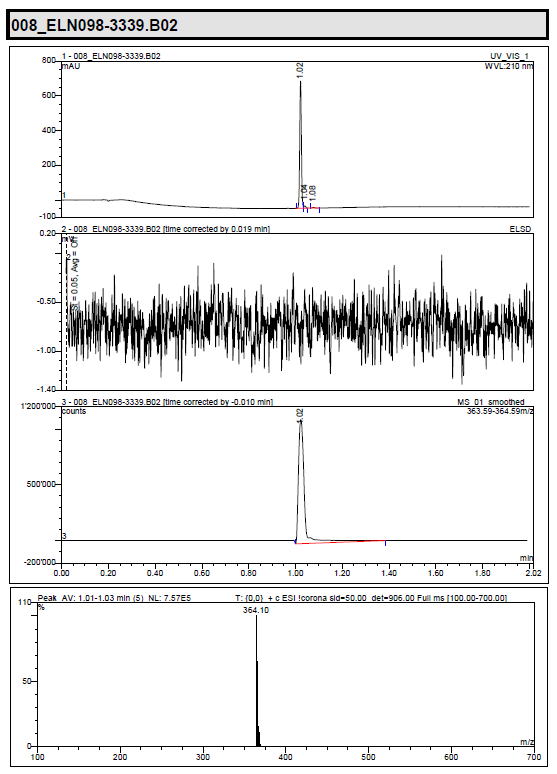

Supplement: Supplementary file 2 — jm4c02941_si_002.zip [file jm4c02941_si_002.zip › cpd 8.png]

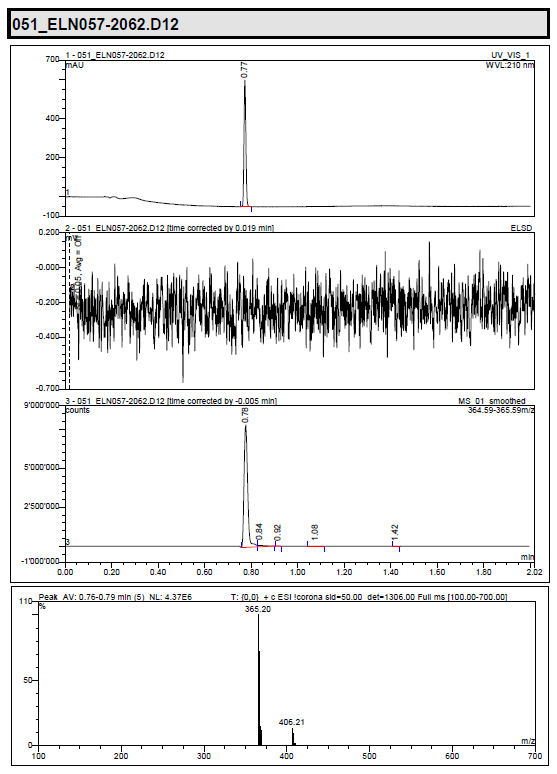

Supplement: Supplementary file 2 — jm4c02941_si_002.zip [file jm4c02941_si_002.zip › cpd 9.png]

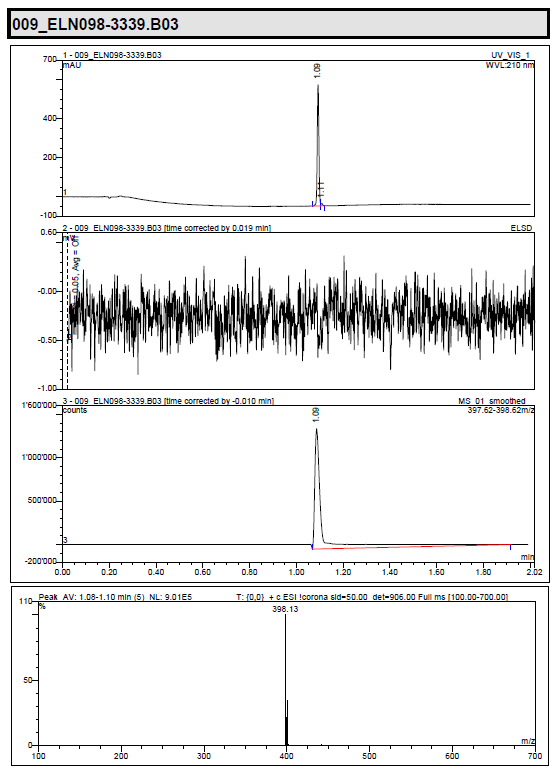

Supplement: Supplementary file 2 — jm4c02941_si_002.zip [file jm4c02941_si_002.zip › cpd 10.png]

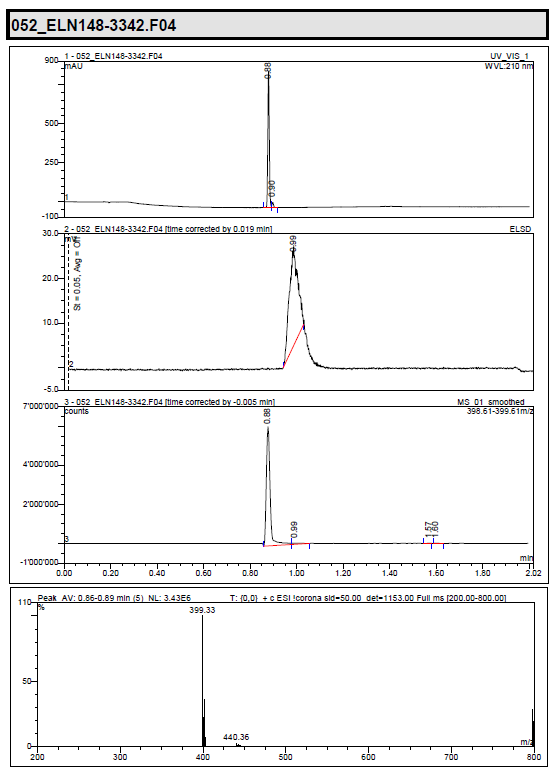

Supplement: Supplementary file 2 — jm4c02941_si_002.zip [file jm4c02941_si_002.zip › cpd 11.png]

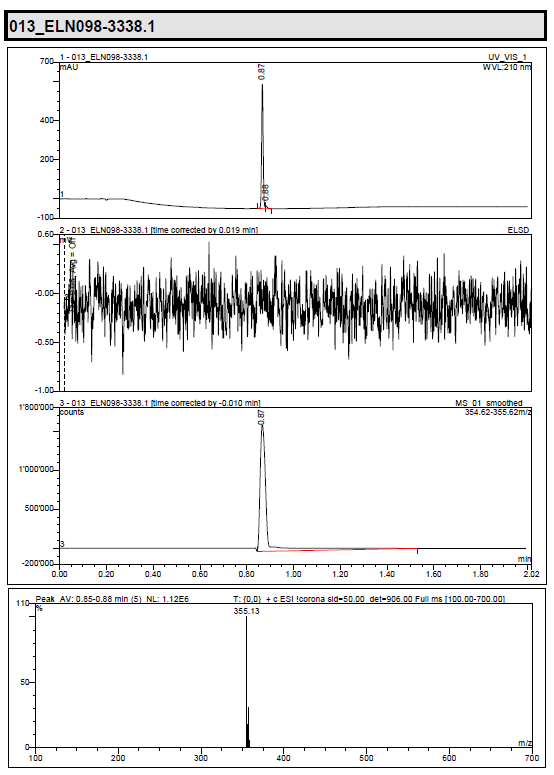

Supplement: Supplementary file 2 — jm4c02941_si_002.zip [file jm4c02941_si_002.zip › cpd 12.png]

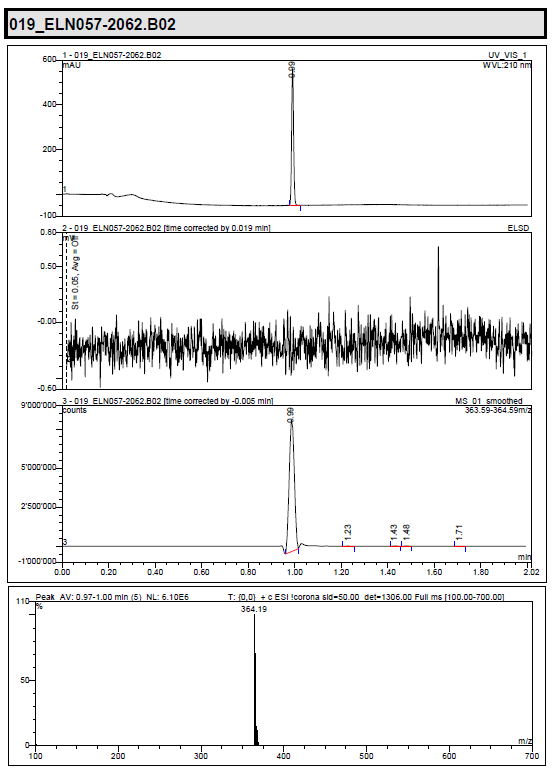

Supplement: Supplementary file 2 — jm4c02941_si_002.zip [file jm4c02941_si_002.zip › cpd 13.png]

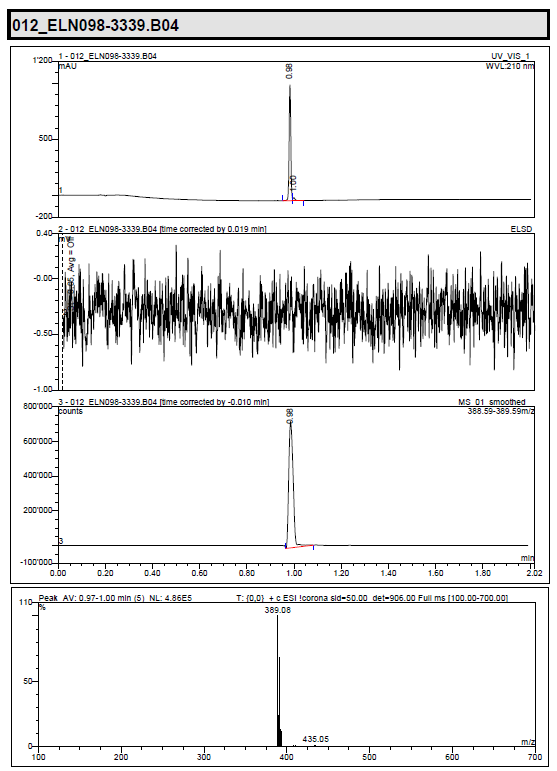

Supplement: Supplementary file 2 — jm4c02941_si_002.zip [file jm4c02941_si_002.zip › cpd 14.png]

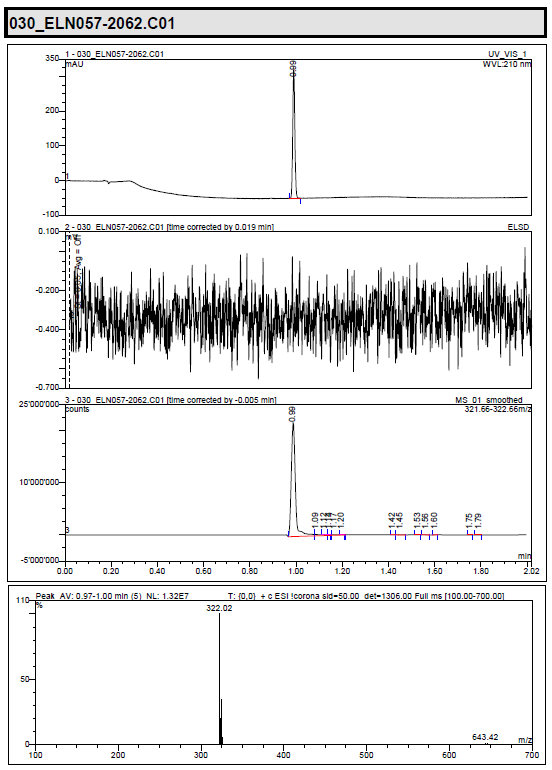

Supplement: Supplementary file 2 — jm4c02941_si_002.zip [file jm4c02941_si_002.zip › cpd 15.png]

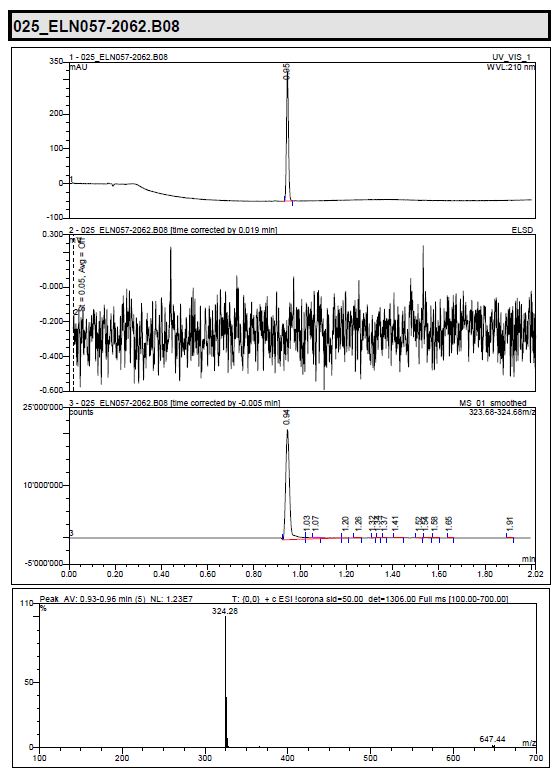

Supplement: Supplementary file 2 — jm4c02941_si_002.zip [file jm4c02941_si_002.zip › cpd 16.png]

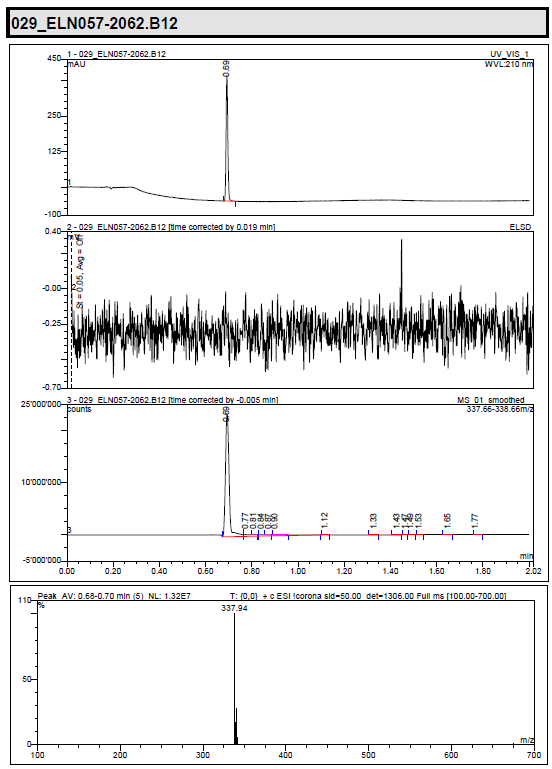

Supplement: Supplementary file 2 — jm4c02941_si_002.zip [file jm4c02941_si_002.zip › cpd 17.png]

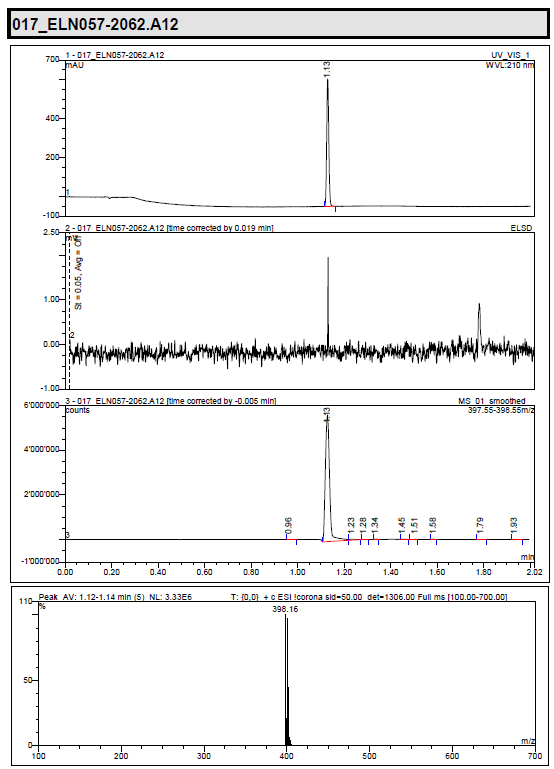

Supplement: Supplementary file 2 — jm4c02941_si_002.zip [file jm4c02941_si_002.zip › cpd 18.png]

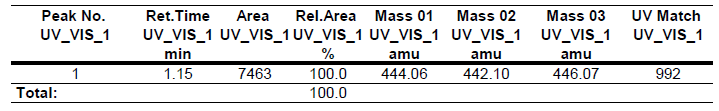

Supplement: Supplementary file 2 — jm4c02941_si_002.zip [file jm4c02941_si_002.zip › cpd 19 mass.png]

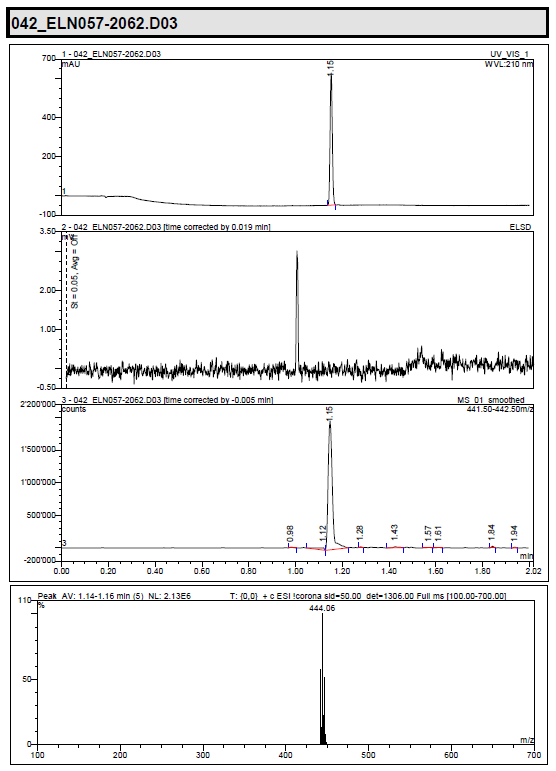

Supplement: Supplementary file 2 — jm4c02941_si_002.zip [file jm4c02941_si_002.zip › cpd 19.png]

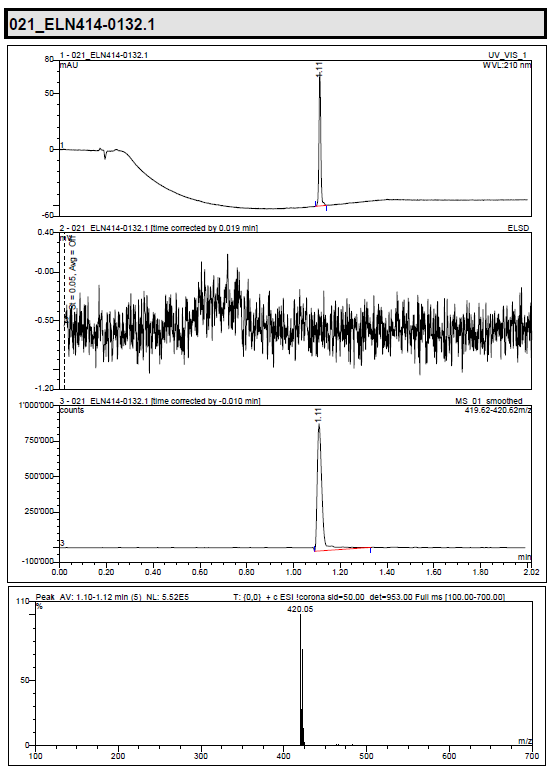

Supplement: Supplementary file 2 — jm4c02941_si_002.zip [file jm4c02941_si_002.zip › cpd 20.png]

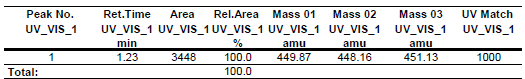

Supplement: Supplementary file 2 — jm4c02941_si_002.zip [file jm4c02941_si_002.zip › cpd 21 mass.png]

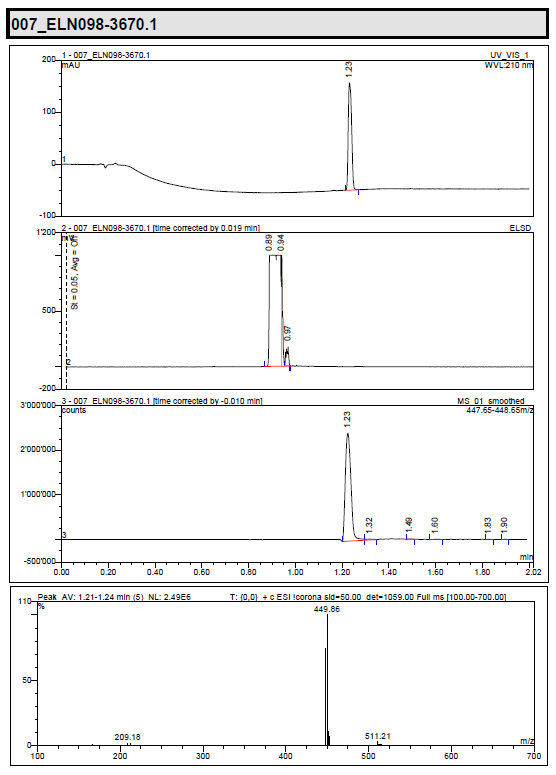

Supplement: Supplementary file 2 — jm4c02941_si_002.zip [file jm4c02941_si_002.zip › cpd 21.png]

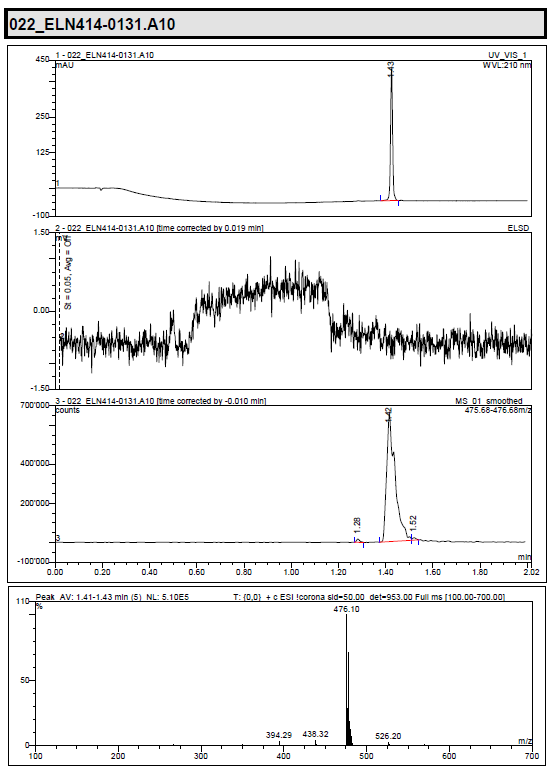

Supplement: Supplementary file 2 — jm4c02941_si_002.zip [file jm4c02941_si_002.zip › cpd 22.png]

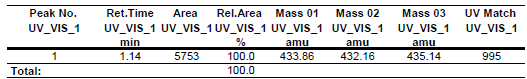

Supplement: Supplementary file 2 — jm4c02941_si_002.zip [file jm4c02941_si_002.zip › cpd 23 mass.png]

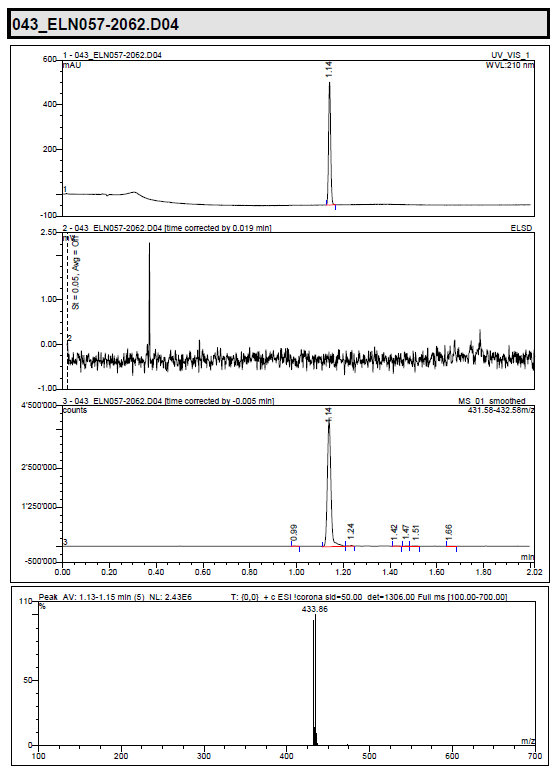

Supplement: Supplementary file 2 — jm4c02941_si_002.zip [file jm4c02941_si_002.zip › cpd 23.png]

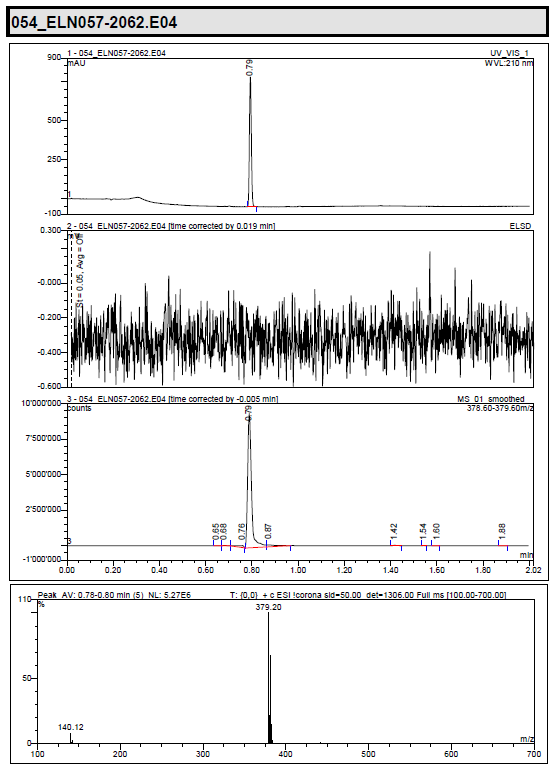

Supplement: Supplementary file 2 — jm4c02941_si_002.zip [file jm4c02941_si_002.zip › cpd 24.png]

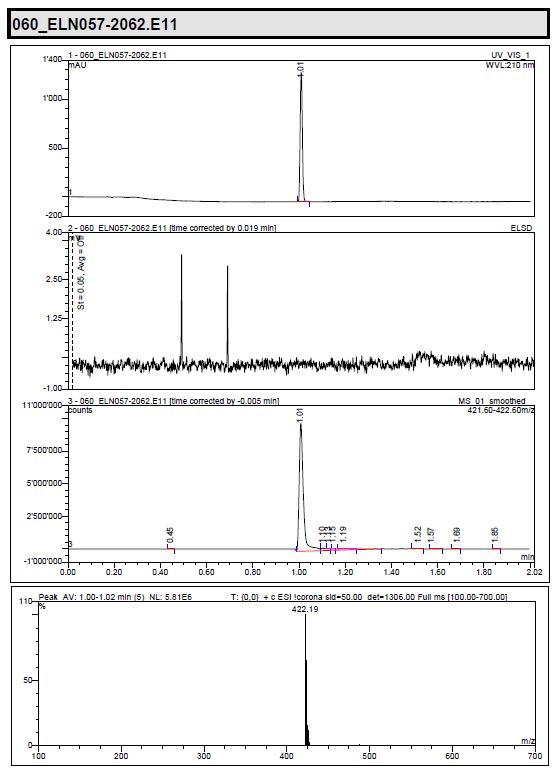

Supplement: Supplementary file 2 — jm4c02941_si_002.zip [file jm4c02941_si_002.zip › cpd 25.png]

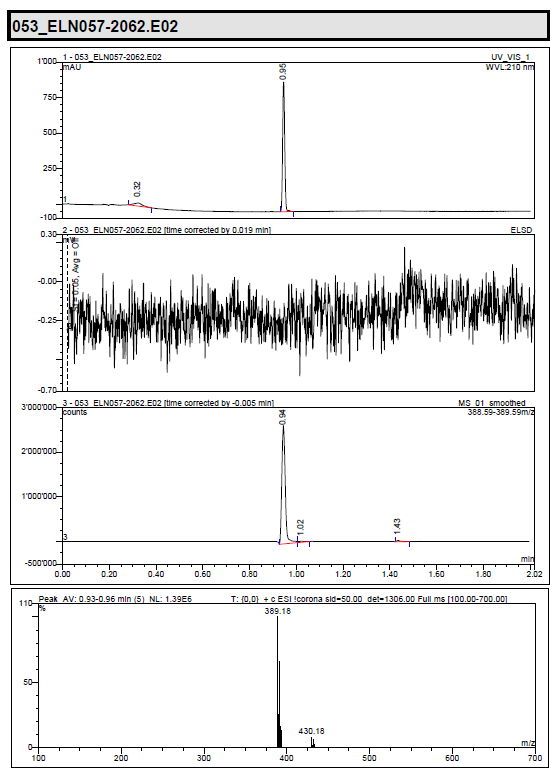

Supplement: Supplementary file 2 — jm4c02941_si_002.zip [file jm4c02941_si_002.zip › cpd 26.png]

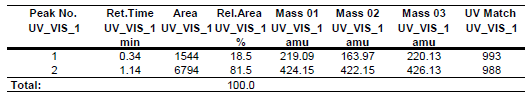

Supplement: Supplementary file 2 — jm4c02941_si_002.zip [file jm4c02941_si_002.zip › cpd 27 mass.png]

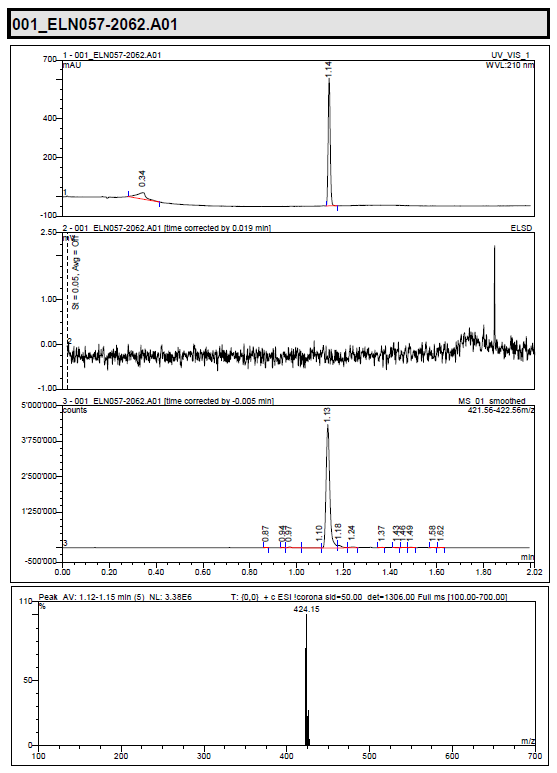

Supplement: Supplementary file 2 — jm4c02941_si_002.zip [file jm4c02941_si_002.zip › cpd 27.png]

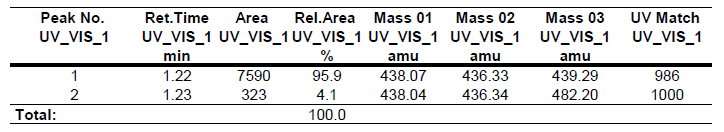

Supplement: Supplementary file 2 — jm4c02941_si_002.zip [file jm4c02941_si_002.zip › cpd 28 mass.png]

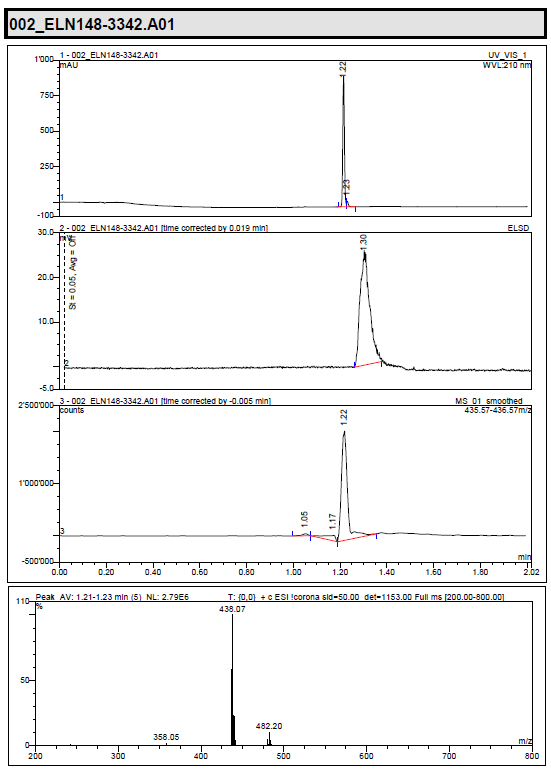

Supplement: Supplementary file 2 — jm4c02941_si_002.zip [file jm4c02941_si_002.zip › cpd 28.png]

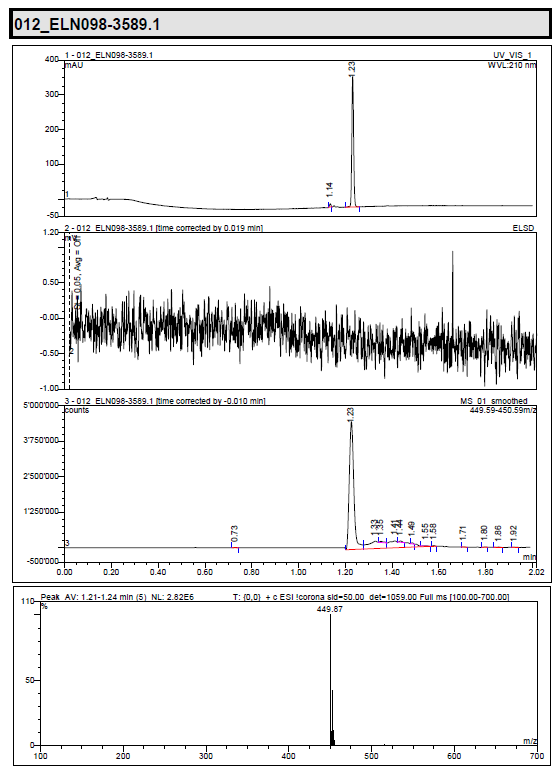

Supplement: Supplementary file 2 — jm4c02941_si_002.zip [file jm4c02941_si_002.zip › cpd 29.png]

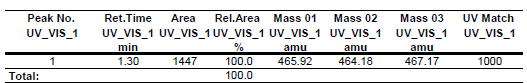

Supplement: Supplementary file 2 — jm4c02941_si_002.zip [file jm4c02941_si_002.zip › cpd 30 mass.png]

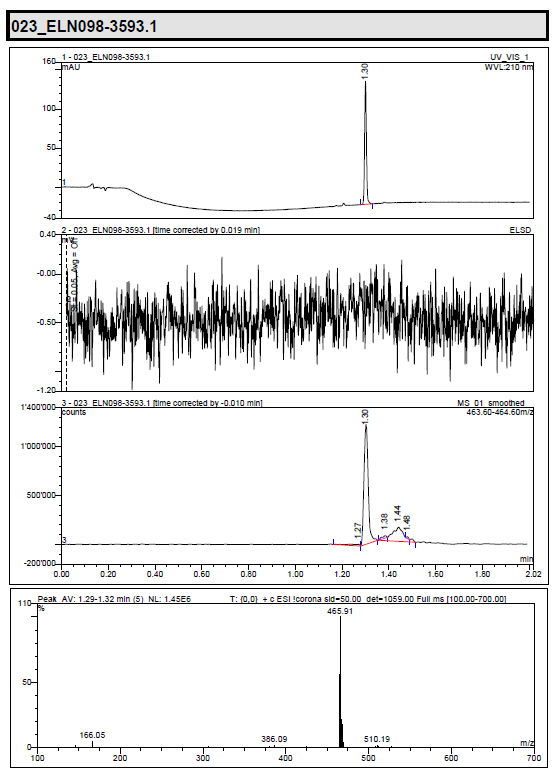

Supplement: Supplementary file 2 — jm4c02941_si_002.zip [file jm4c02941_si_002.zip › cpd 30.png]

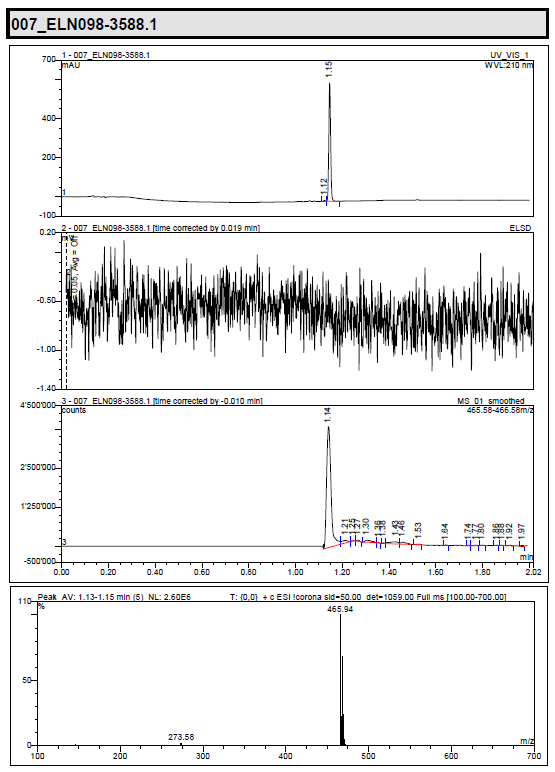

Supplement: Supplementary file 2 — jm4c02941_si_002.zip [file jm4c02941_si_002.zip › cpd 31.png]

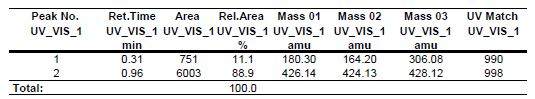

Supplement: Supplementary file 2 — jm4c02941_si_002.zip [file jm4c02941_si_002.zip › cpd 32 mass.png]

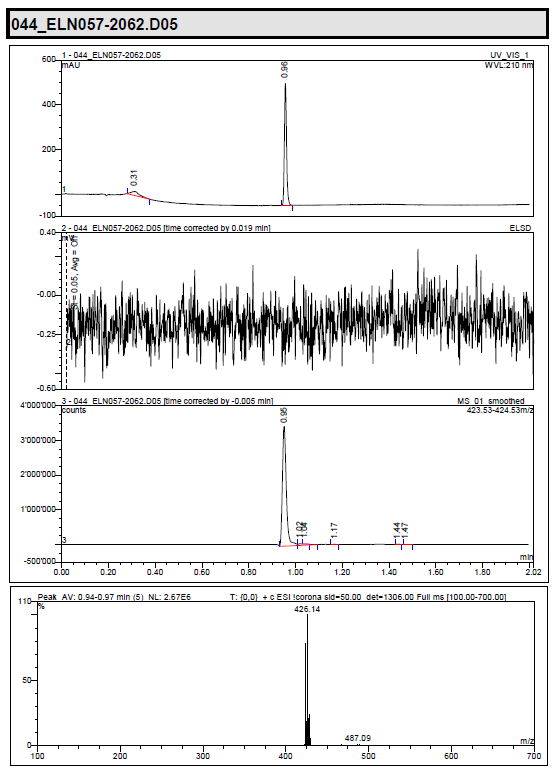

Supplement: Supplementary file 2 — jm4c02941_si_002.zip [file jm4c02941_si_002.zip › cpd 32.png]

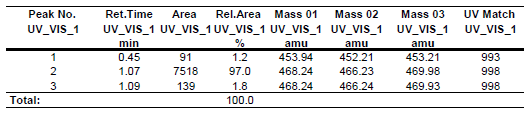

Supplement: Supplementary file 2 — jm4c02941_si_002.zip [file jm4c02941_si_002.zip › cpd 33 mass.png]

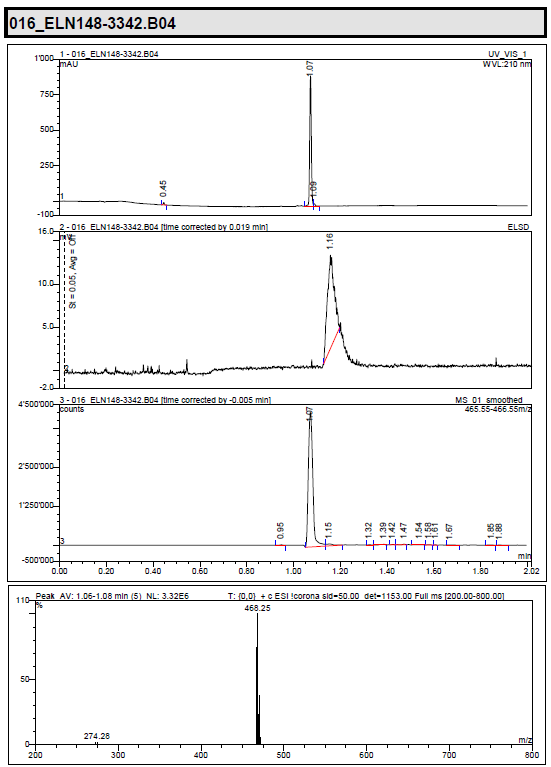

Supplement: Supplementary file 2 — jm4c02941_si_002.zip [file jm4c02941_si_002.zip › cpd 33.png]

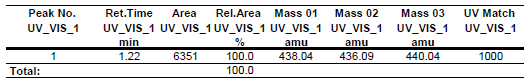

Supplement: Supplementary file 2 — jm4c02941_si_002.zip [file jm4c02941_si_002.zip › cpd 34 mass.png]

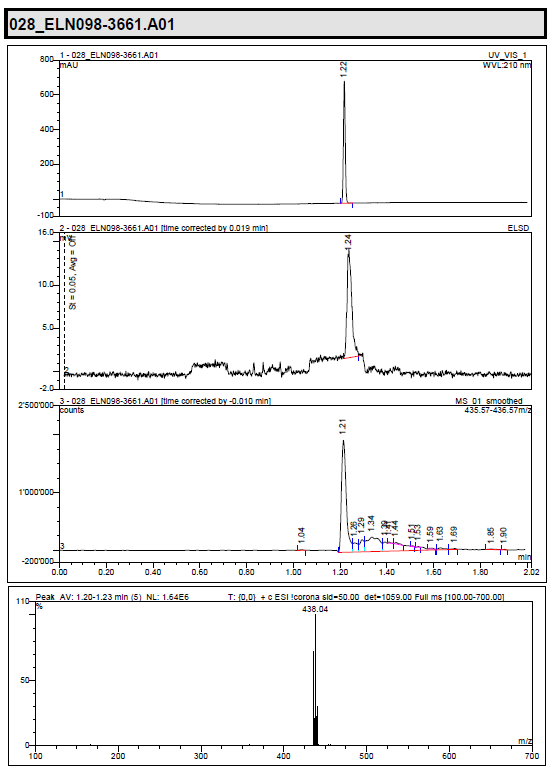

Supplement: Supplementary file 2 — jm4c02941_si_002.zip [file jm4c02941_si_002.zip › cpd 34.png]

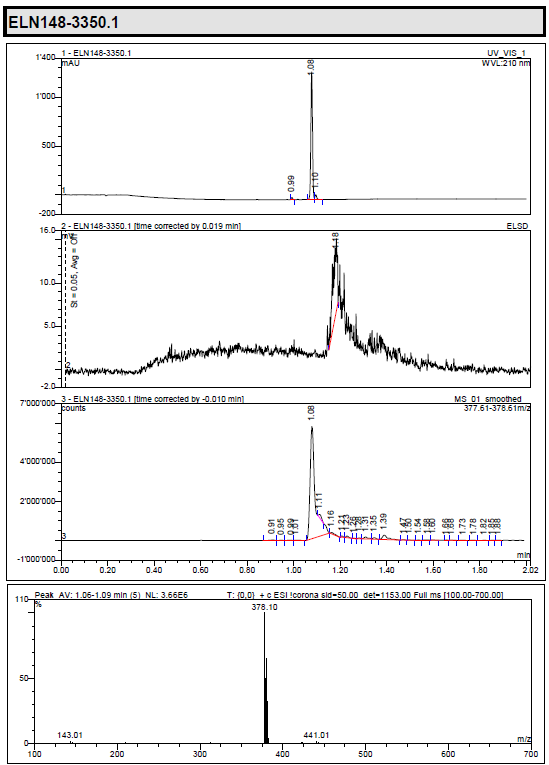

Supplement: Supplementary file 2 — jm4c02941_si_002.zip [file jm4c02941_si_002.zip › cpd 35.png]

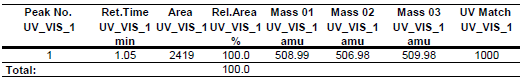

Supplement: Supplementary file 2 — jm4c02941_si_002.zip [file jm4c02941_si_002.zip › cpd 36 mass.png]

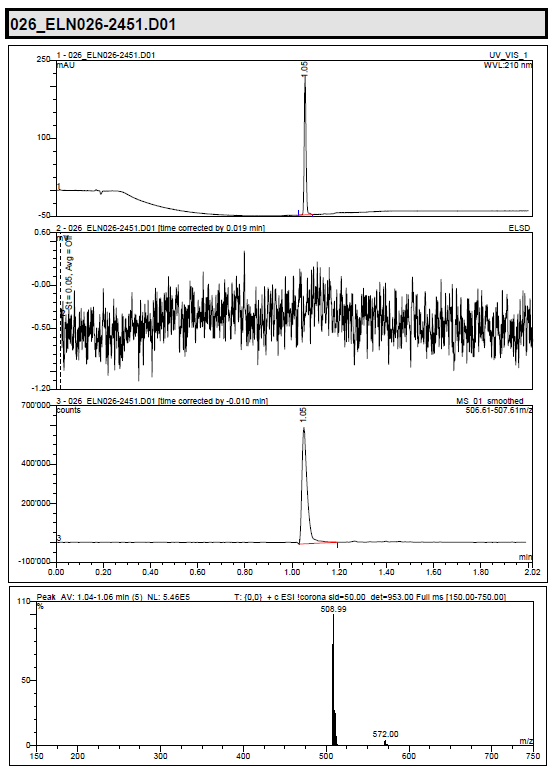

Supplement: Supplementary file 2 — jm4c02941_si_002.zip [file jm4c02941_si_002.zip › cpd 36.png]

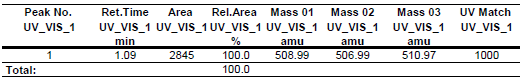

Supplement: Supplementary file 2 — jm4c02941_si_002.zip [file jm4c02941_si_002.zip › cpd 37 mass.png]

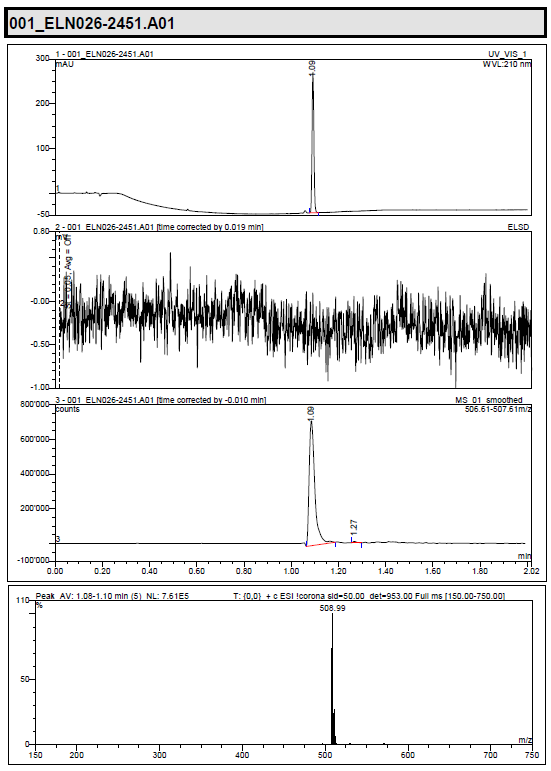

Supplement: Supplementary file 2 — jm4c02941_si_002.zip [file jm4c02941_si_002.zip › cpd 37.png]

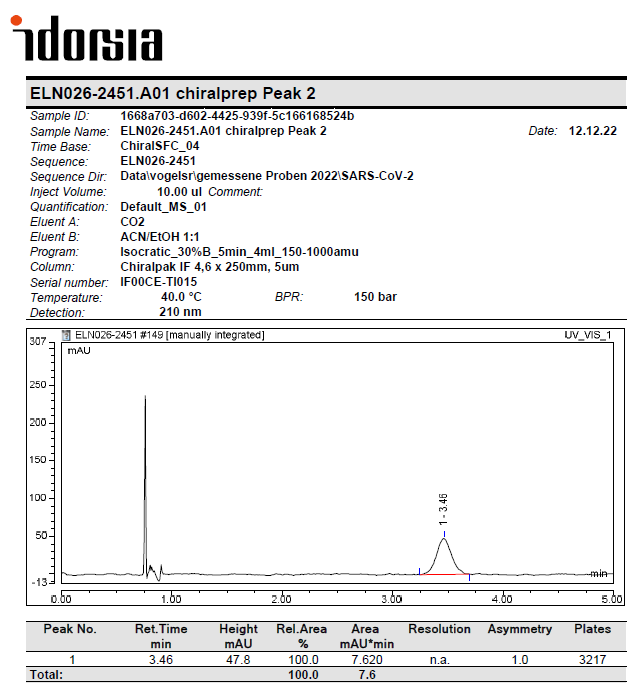

Supplement: Supplementary file 2 — jm4c02941_si_002.zip [file jm4c02941_si_002.zip › cpd 37a.png]

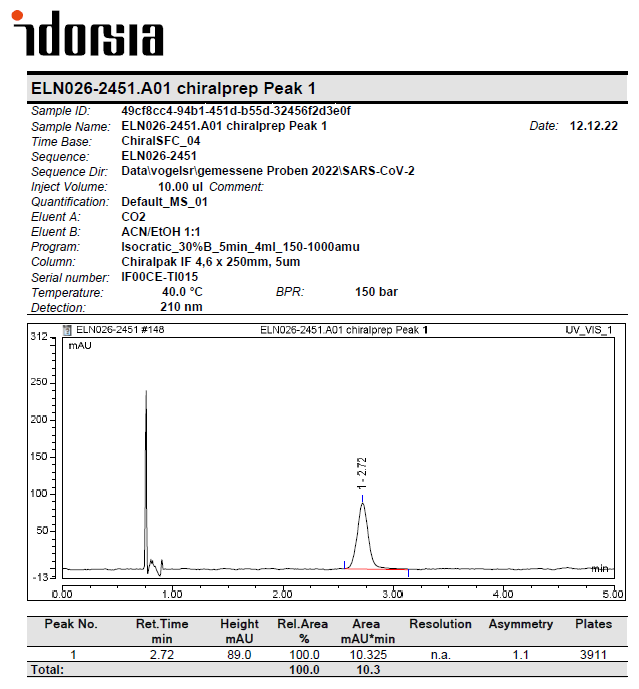

Supplement: Supplementary file 2 — jm4c02941_si_002.zip [file jm4c02941_si_002.zip › cpd 37b.png]

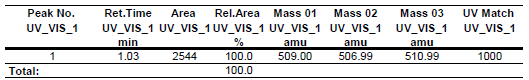

Supplement: Supplementary file 2 — jm4c02941_si_002.zip [file jm4c02941_si_002.zip › cpd 38 mass.png]

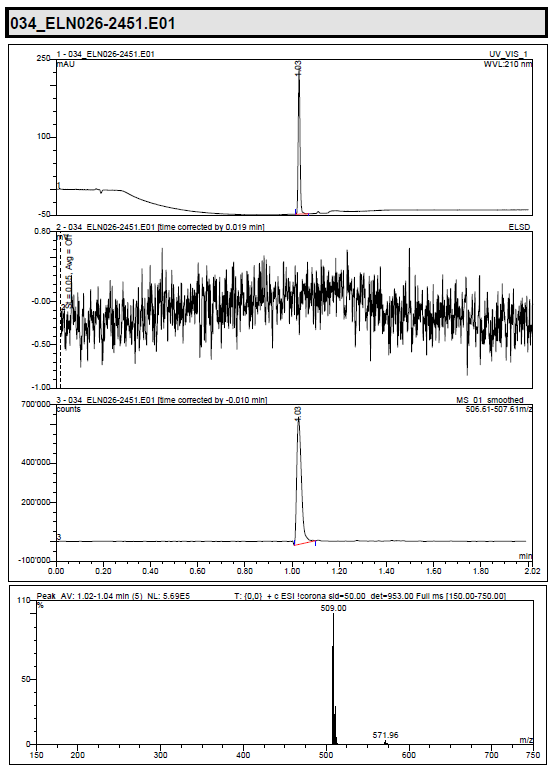

Supplement: Supplementary file 2 — jm4c02941_si_002.zip [file jm4c02941_si_002.zip › cpd 38.png]

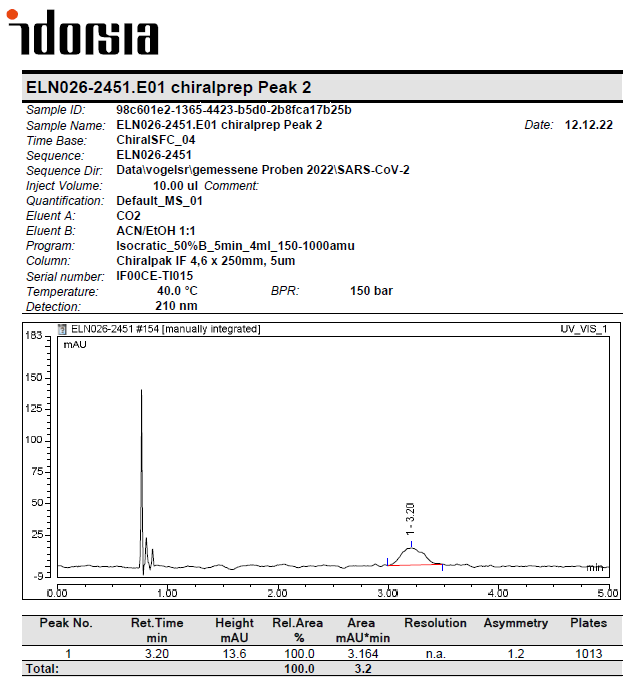

Supplement: Supplementary file 2 — jm4c02941_si_002.zip [file jm4c02941_si_002.zip › cpd 38a.png]

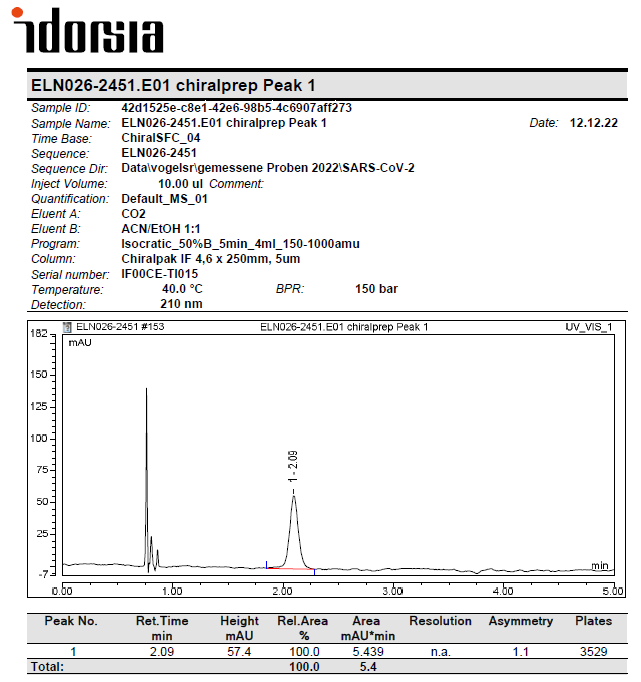

Supplement: Supplementary file 2 — jm4c02941_si_002.zip [file jm4c02941_si_002.zip › cpd 38b.png]

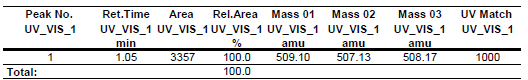

Supplement: Supplementary file 2 — jm4c02941_si_002.zip [file jm4c02941_si_002.zip › cpd 39 mass.png]

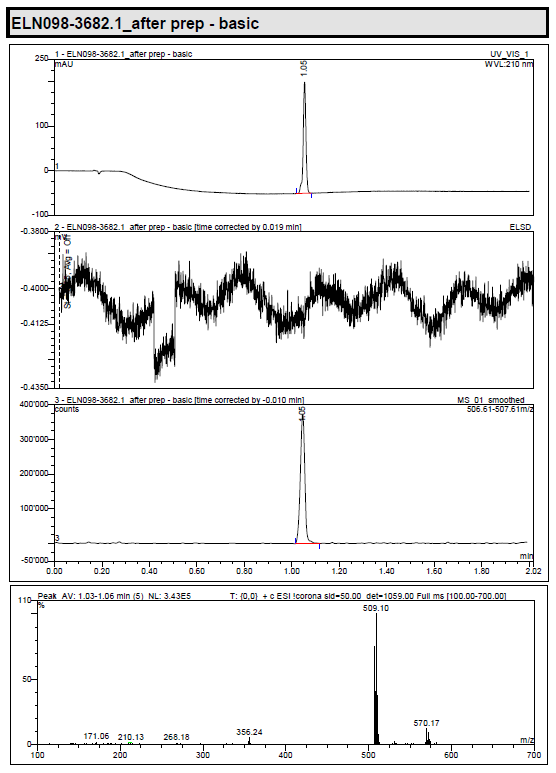

Supplement: Supplementary file 2 — jm4c02941_si_002.zip [file jm4c02941_si_002.zip › cpd 39.png]

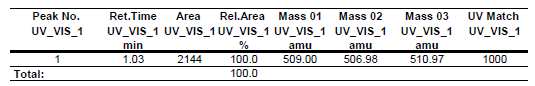

Supplement: Supplementary file 2 — jm4c02941_si_002.zip [file jm4c02941_si_002.zip › cpd 40 mass.png]

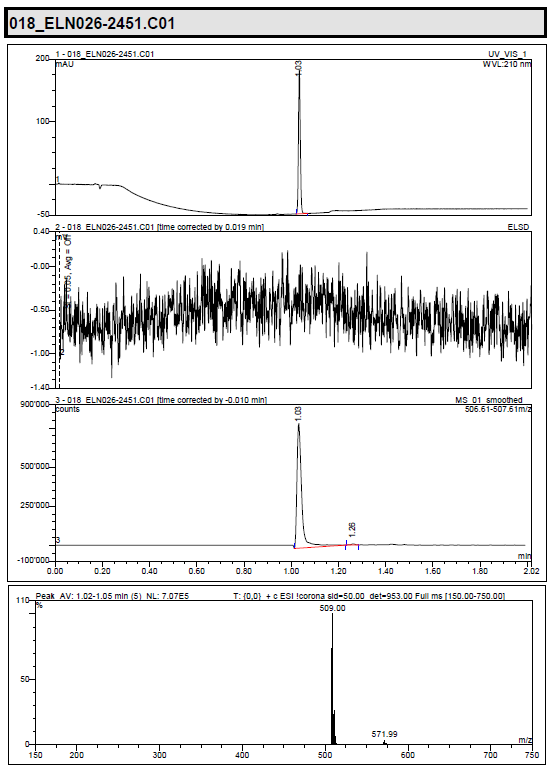

Supplement: Supplementary file 2 — jm4c02941_si_002.zip [file jm4c02941_si_002.zip › cpd 40.png]

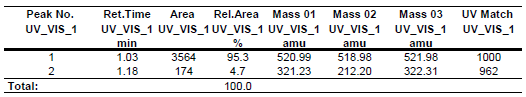

Supplement: Supplementary file 2 — jm4c02941_si_002.zip [file jm4c02941_si_002.zip › cpd 61 mass.png]

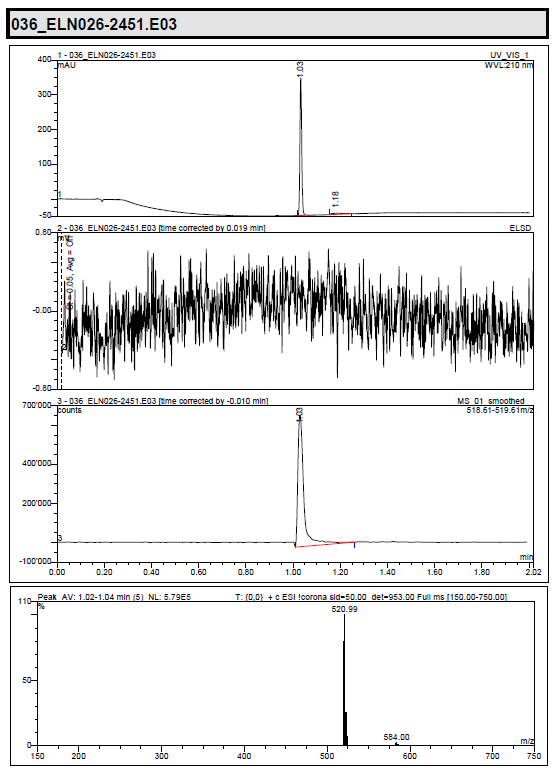

Supplement: Supplementary file 2 — jm4c02941_si_002.zip [file jm4c02941_si_002.zip › cpd 61.png]

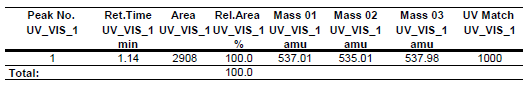

Supplement: Supplementary file 2 — jm4c02941_si_002.zip [file jm4c02941_si_002.zip › cpd 62 mass.png]

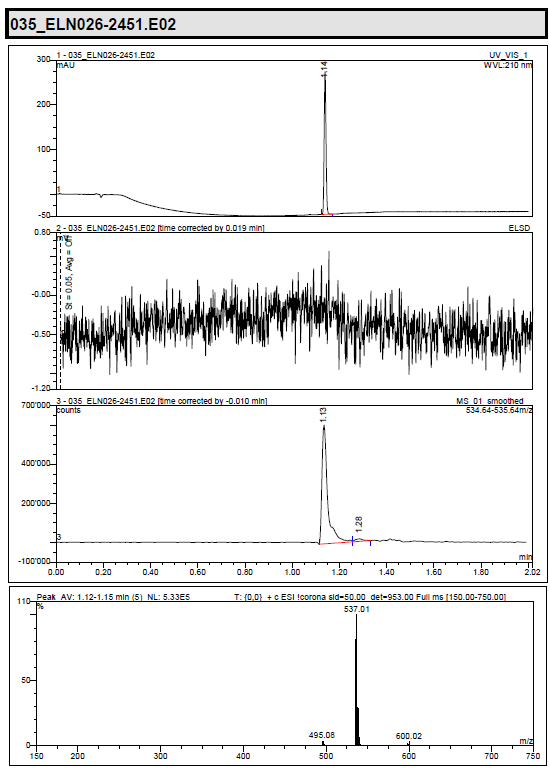

Supplement: Supplementary file 2 — jm4c02941_si_002.zip [file jm4c02941_si_002.zip › cpd 62.png]

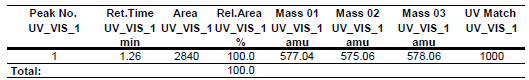

Supplement: Supplementary file 2 — jm4c02941_si_002.zip [file jm4c02941_si_002.zip › cpd 63 mass.png]

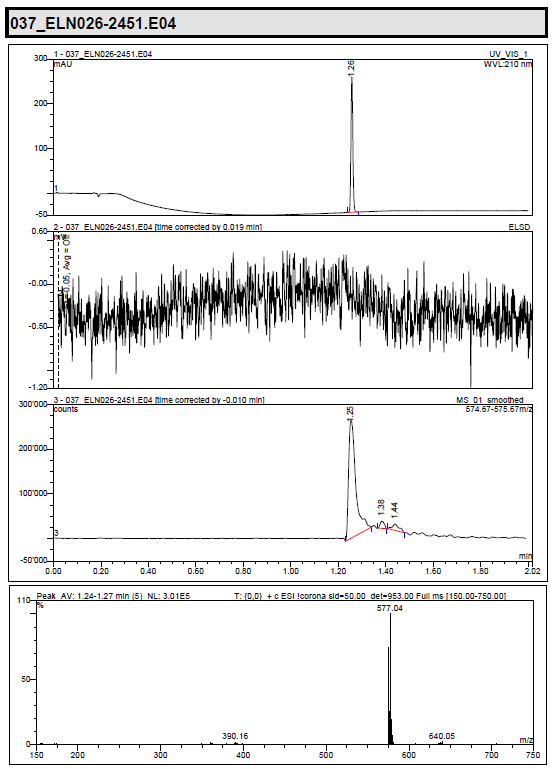

Supplement: Supplementary file 2 — jm4c02941_si_002.zip [file jm4c02941_si_002.zip › cpd 63.png]

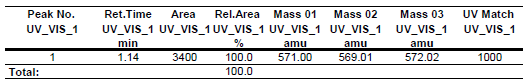

Supplement: Supplementary file 2 — jm4c02941_si_002.zip [file jm4c02941_si_002.zip › cpd 64 mass.png]

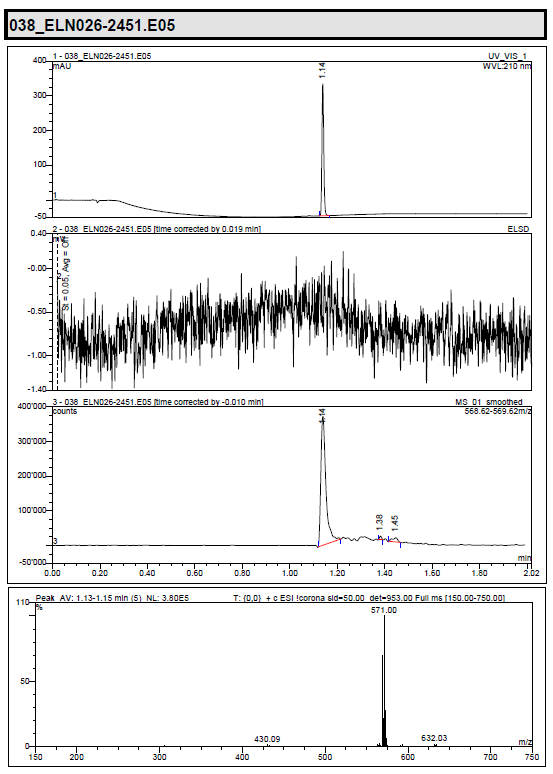

Supplement: Supplementary file 2 — jm4c02941_si_002.zip [file jm4c02941_si_002.zip › cpd 64.png]

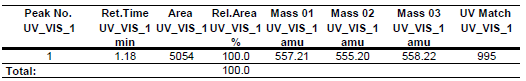

Supplement: Supplementary file 2 — jm4c02941_si_002.zip [file jm4c02941_si_002.zip › cpd 65 mass.png]

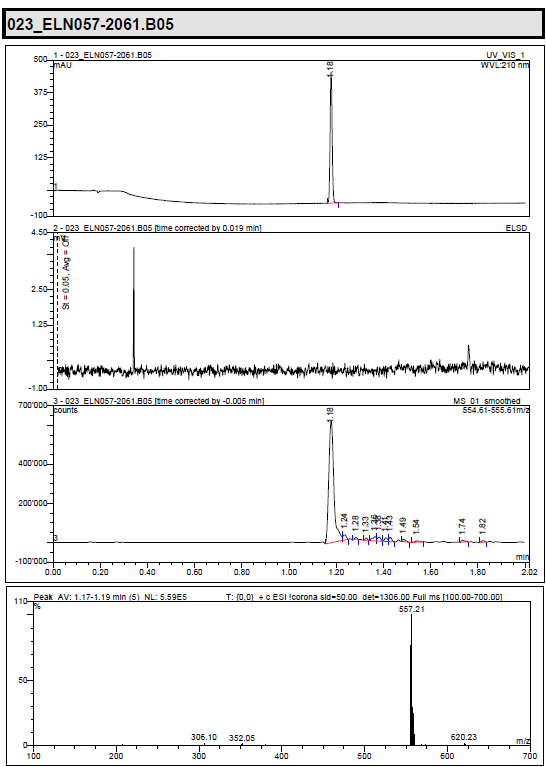

Supplement: Supplementary file 2 — jm4c02941_si_002.zip [file jm4c02941_si_002.zip › cpd 65.png]

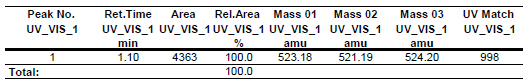

Supplement: Supplementary file 2 — jm4c02941_si_002.zip [file jm4c02941_si_002.zip › cpd 66 mass.png]

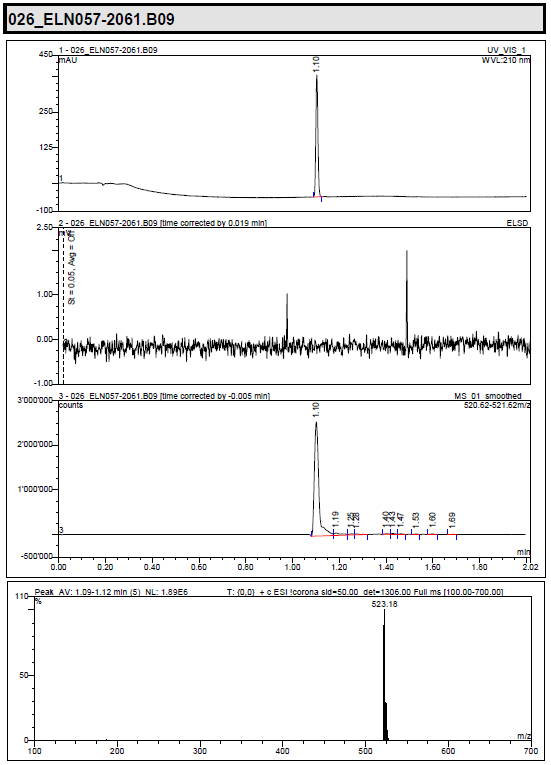

Supplement: Supplementary file 2 — jm4c02941_si_002.zip [file jm4c02941_si_002.zip › cpd 66.png]

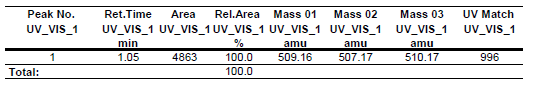

Supplement: Supplementary file 2 — jm4c02941_si_002.zip [file jm4c02941_si_002.zip › cpd 67 mass.png]

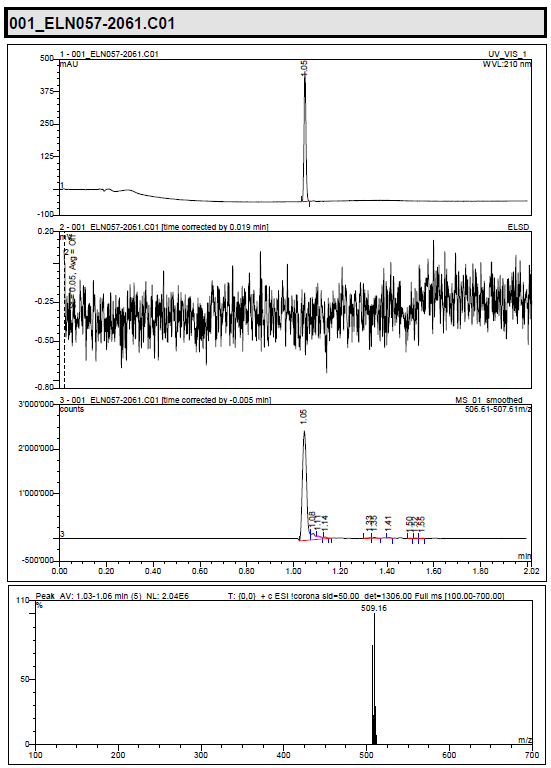

Supplement: Supplementary file 2 — jm4c02941_si_002.zip [file jm4c02941_si_002.zip › cpd 67.png]

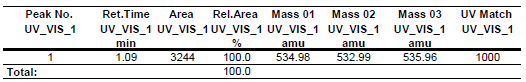

Supplement: Supplementary file 2 — jm4c02941_si_002.zip [file jm4c02941_si_002.zip › cpd 68 mass.png]

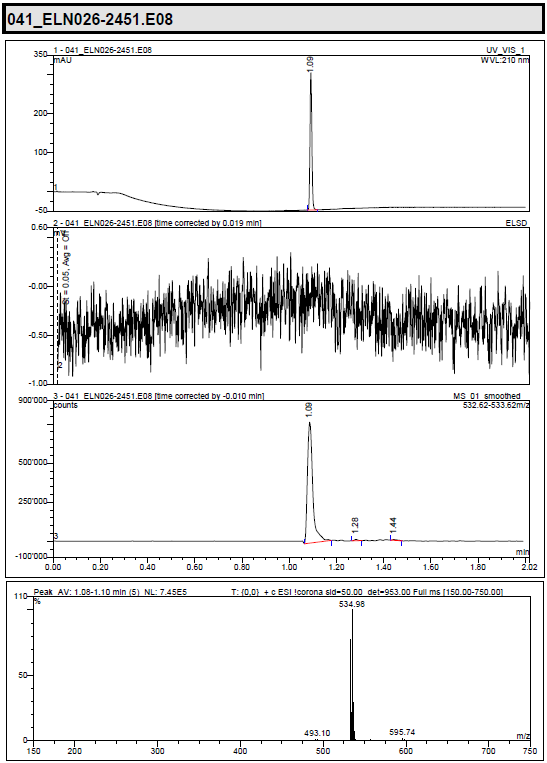

Supplement: Supplementary file 2 — jm4c02941_si_002.zip [file jm4c02941_si_002.zip › cpd 68.png]

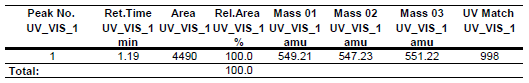

Supplement: Supplementary file 2 — jm4c02941_si_002.zip [file jm4c02941_si_002.zip › cpd 69 mass.png]

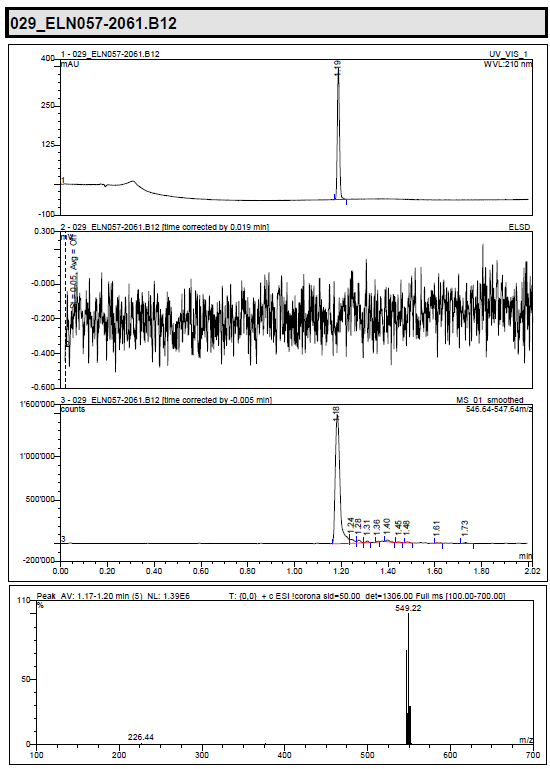

Supplement: Supplementary file 2 — jm4c02941_si_002.zip [file jm4c02941_si_002.zip › cpd 69.png]

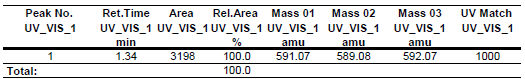

Supplement: Supplementary file 2 — jm4c02941_si_002.zip [file jm4c02941_si_002.zip › cpd 70 mass.png]

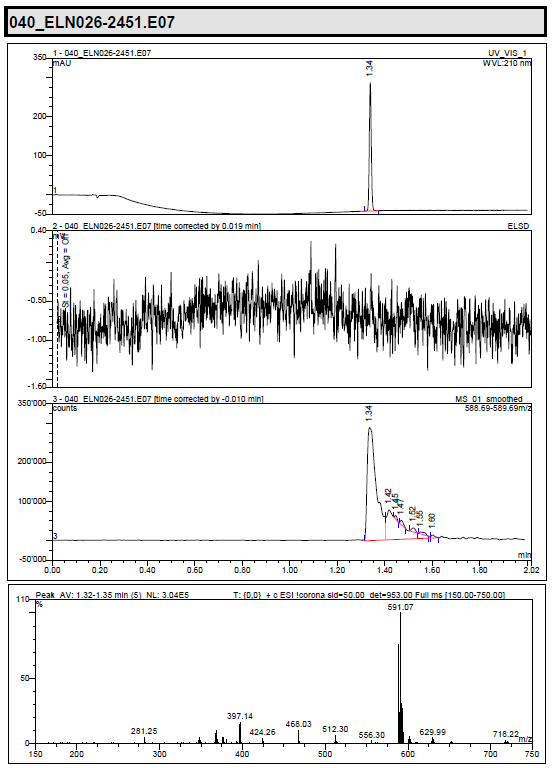

Supplement: Supplementary file 2 — jm4c02941_si_002.zip [file jm4c02941_si_002.zip › cpd 70.png]

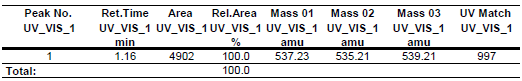

Supplement: Supplementary file 2 — jm4c02941_si_002.zip [file jm4c02941_si_002.zip › cpd 71 mass.png]

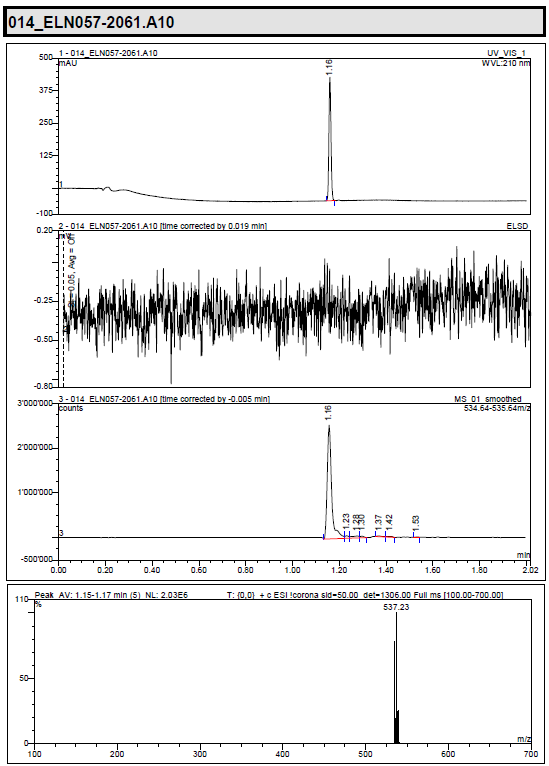

Supplement: Supplementary file 2 — jm4c02941_si_002.zip [file jm4c02941_si_002.zip › cpd 71.png]
